# Simulation-Guided Engineering Enables a Functional Switch in Selinadiene Synthase toward Hydroxylation

**DOI:** 10.1021/acscatal.4c02032

**Published:** 2024-07-09

**Authors:** Prabhakar L. Srivastava, Sam T. Johns, Angus Voice, Katharine Morley, Andrés M. Escorcia, David J. Miller, Rudolf K. Allemann, Marc W. van der Kamp

**Affiliations:** † School of Chemistry, Cardiff University, Main Building, Park Place, Cardiff CF10 3AT, U.K.; ‡ School of Biochemistry, University of Bristol, University Walk, Bristol BS8 1TD, U.K.

**Keywords:** terpenoids, MD simulation, water capture, enzyme engineering, selin-7(11)-en-4-ol

## Abstract

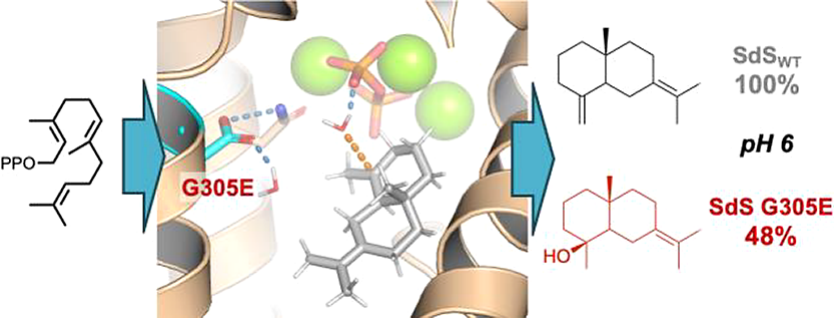

Engineering sesquiterpene synthases to form predefined alternative products is a major challenge due to their diversity in cyclization mechanisms and our limited understanding of how amino acid changes affect the steering of these mechanisms. Here, we use a combination of atomistic simulation and site-directed mutagenesis to engineer a selina-4(15),7(11)-diene synthase (SdS) such that its final reactive carbocation is quenched by trapped active site water, resulting in the formation of a complex hydroxylated sesquiterpene (selin-7(11)-en-4-ol). Initially, the SdS G305E variant produced 20% selin-7(11)-en-4-ol. As suggested by modeling of the enzyme-carbocation complex, selin-7(11)-en-4-ol production could be further improved by varying the pH, resulting in selin-7(11)-en-4-ol becoming the major product (48%) at pH 6.0. We incorporated the SdS G305E variant along with genes from the mevalonate pathway into bacterial BL21(DE3) cells and demonstrated the production of selin-7(11)-en-4-ol at a scale of 10 mg/L in batch fermentation. These results highlight opportunities for the simulation-guided engineering of terpene synthases to produce predefined complex hydroxylated sesquiterpenes.

## Introduction

Terpenoids are the most widespread and largest class of natural products and have diverse biological applications ranging from being useful in agriculture, perfume, and cosmetic industries to pharma industries.^1−6^ In nature, terpenoids are produced from common isoprenyl diphosphate precursors, which are biosynthesized via either the mevalonate (MEV) or the 1-deoxyxylulose-5-phosphate (DXP) pathway typically in minute quantities in their host systems.^7−10^ Sesquiterpene hydrocarbons are biosynthesized through the conversion of (2*E*,6*E*)-farnesyl diphosphate (**1**, FDP) in reactions catalyzed by class 1 sesquiterpene synthases.^1,11,12^ Sesquiterpene synthases catalyze some of the most challenging chemical reactions in nature, starting with Mg^2+^-dependent diphosphate cleavage of (2*E*,6*E*)-FDP to give rise to a highly reactive carbocation.^12−14^ This reactive carbocation can undergo a cascade of various intramolecular rearrangements that can involve several carbocationic and/or neutral intermediates and ultimate products are formed by deprotonation or H_2_O attack.^1,15−18^ The main challenge in engineering terpene biosynthesis is controlling the intramolecular arrangements of highly reactive carbocations and the final product distribution.^19−21^ The final carbocation quench most frequently occurs by proton loss to give hydrocarbon terpenes. Quenching by water to give specific, complex terpene alcohols requires a high level of molecular choreography in order to make water available while avoiding premature quenching of reactive intermediates since the presence of trapped water is likely ubiquitous within the closed active site conformation.^22−25^ Numerous studies have shown that (sesqui)terpene synthases are highly promiscuous in nature and slight changes in the active site pocket could lead to drastic changes in the product distribution, including the formation of hydroxylated sesquiterpenes.^25−30^ Engineering of sesquiterpene synthases to create novel and high-fidelity enzymes supports the establishment of biotechnological processes for the manufacturing of highly valuable sesquiterpenoids.^20,31−33^ However, the predictive engineering of these enzymes to make specific products remains a major challenge.

**Scheme 1:**
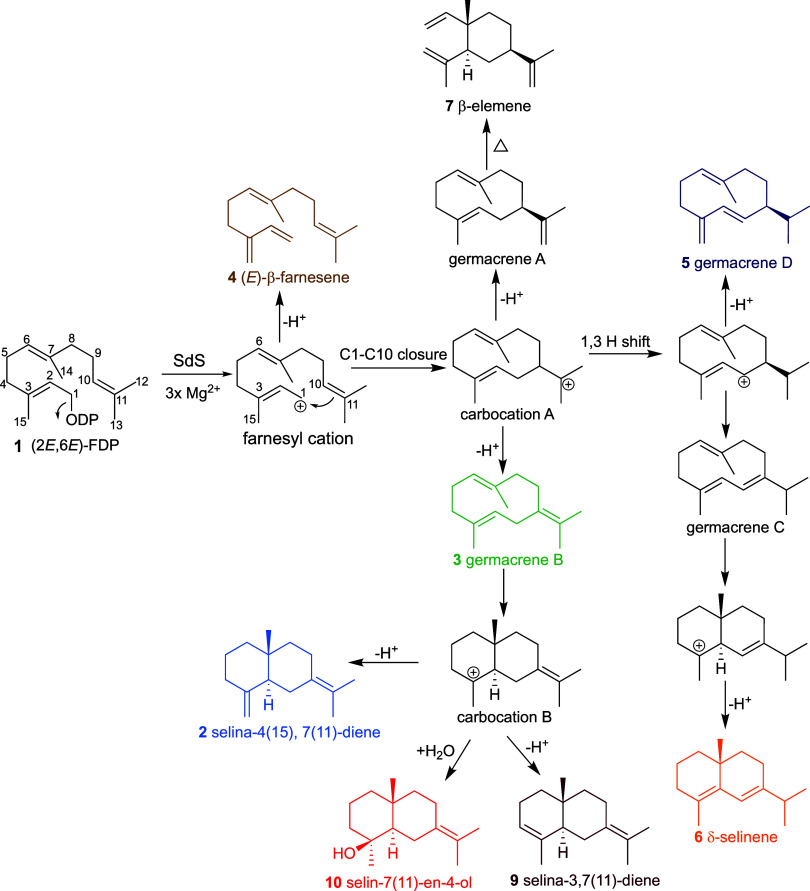
Proposed Cyclization Mechanism of Sesquiterpene Products Generated by SdS and Its Variants

Selina-4(15),7(11)-diene synthase (SdS) from *Streptomyces pristinaespiralis* ATCC 25486 catalyzes a multistep reaction that converts (2*E*,6*E*)-FDP (**1**) into cyclic selina-4(15),7(11)-diene (**2**) as a major product along with small percentage of germacrene B (**3**).^23,34^ Our recent work on avoiding water capture in hydroxylating sesquiterpene synthases has generated an understanding of how these enzymes employ water molecules to produce hydroxylated sesquiterpenes.^22^ Here, we describe our efforts to introduce water capture in a non-hydroxylating sesquiterpene synthase to from a predefined sesquiterpene alcohol by quenching the final reactive carbocation. To this end, we carried out molecular dynamics (MD) simulation-guided site-directed mutagenesis in the active site pocket of SdS. G305 in the K-helix region was identified as a key residue which, upon alteration to E305, led to the formation of sesquiterpene alcohol selin-7(11)-en-4-ol (**10**, 20%). Our (quantum mechanical/molecular mechanical) simulations then indicate how in the G305E variant water attack at carbocation B can take place to form selin-7(11)-en-4-ol, which alternatively undergoes deprotonation to form selina-4(15),7(11)-diene (as in wild-type SdS; Scheme 1). To improve the formation of selin-7(11)-en-4-ol by the G305E variant, we optimized the pH, resulting in a significant increase in the formation of selin-7(11)-en-4-ol (as the major product at ∼48% at pH 6.0) without compromising the kinetic efficiency of the enzyme. We further demonstrate that using SdS G305E in vivo, together with metabolic engineering, is a viable route to selin-7(11)-en-4-ol production. Selin-7(11)-en-4-ol has been isolated from flowering plants *Dipterocarpus cornutus*,^35^*Acritopappus prunifolium*^36^ and *Laggera pterodonta*(37) and has been synthesized chemically,^38^ but no enzymatic route is known for the biosynthesis of this molecule. Hence, our work establishes SdS G305E at pH 6.0 as the first known “selin-7(11)-en-4-ol synthase”, i.e., a sesquiterpene synthase that produces selin-7(11)-en-4-ol as its major product. The findings reported here pave the way for engineering non-hydroxylating sesquiterpene synthases to produce predefined sesquiterpene alcohols by selective changes in the active site pocket, which could then be produced at scale through metabolic engineering so that, for example, their potential biological activities can be explored.

## Results and Discussion

### Establishing the Product Profile of Selina-4(15),7(11)-diene Synthase (SdS)

Selina-4(15),7(11)-diene synthase (SdS_WT_) from *Streptomyces pristinaespiralis* is a non-hydroxylating sesquiterpene synthase which catalyzes the formation of selina-4(15),7(11)-diene (**2**) and germacrene B (**3**) using acyclic (2*E*,6*E*)-FDP (**1**) as a substrate (Scheme 1).^34^ For the engineering of SdS to utilize active site water to produce predefined hydroxylated sesquiterpene(s), codon-optimized SdS (B5HDJ6) was cloned into a pET28a expression vector and overexpressed in *E. coli* and purified to the homogeneity (see the Supporting Information for details). Incubation of SdS_WT_ with (2*E*,6*E*)-FDP resulted in the formation of **2** (86.7%) and **3** (13.3%), a similar product distribution to earlier reports.^34^ Germacrene B (**3**) is generated as a neutral intermediate by deprotonation from C10 in carbocation A. It can undergo further rearrangement to form carbocation B, which is deprotonated at C15 to form selina-4(15),7(11)-diene (Scheme 1). Kinetic parameters for SdS_WT_ were determined using 1-^3^H labeled (2*E*,6*E*)-FDP, giving *K*_M_ 0.87 ± 0.11 μM and *k*_cat_ 7.0 ± 0.02 × 10^–3^ s^–1^. This is consistent with the range of kinetic constants reported for other sesquiterpene synthases from bacteria and plants.^24,29,30,39−41^

### Selection and Simulation-Based Evaluation of Possible SdS Variants

**Figure 1:**
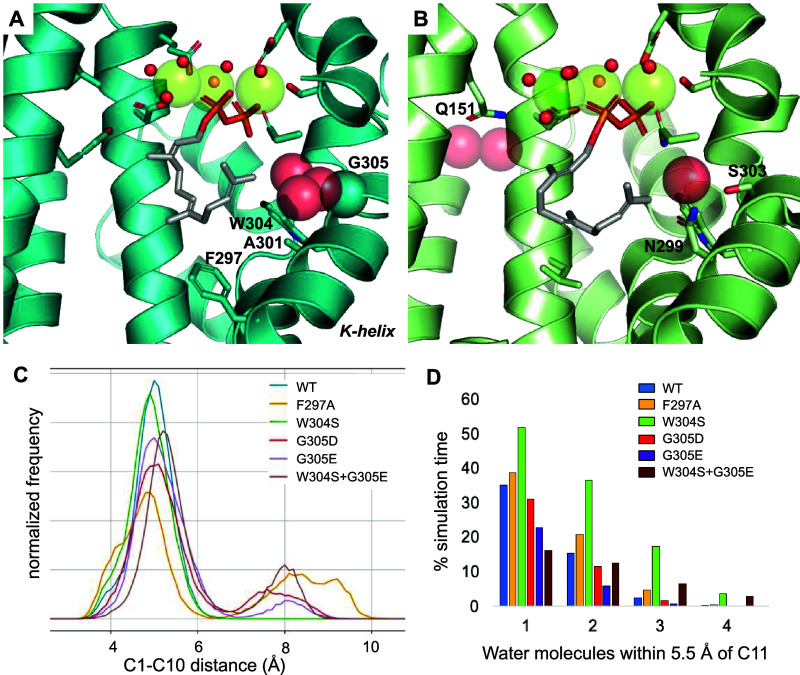
FDP conformation and water presence in non-hydroxylating sesquiterpene synthases. (A) SdS_WT_ in complex with FDP (based on PDB code 4OKZ).^34^ (B) Aristolochene synthase from *Aspergillus terreus* in complex with FDP (based on PDB code 4KUX).^43^ In panels A and B, water positions in the active site are indicated by red spheres, with large transparent spheres for those that do not coordinate to the Mg^2+^ ions (green spheres). Residues discussed in the text are labeled. (C) C1–C10 distance histograms (0.1 Å bins) obtained from 10× 30 ns MD simulations for each variant. (D) Percentage of simulation time that 1, 2, 3, or 4 water molecules are within 5.5 Å of C11.

To explore the possibility of engineering SdS to generate a sesquiterpene synthase that synthesizes a cyclized and hydroxylated product, we considered that subtle changes in the active site would be best, to avoid linear (or other noncomplex) products due to premature carbocation quenching, which can be the result of significant changes in the active site contour.^22,25,42^ We first suggest mutations based on the crystal structure of SdS in complex with the substrate analogue 2,3-dihydrofarnesyl diphosphate (DHFDP) and compare to previous results on other sesquiterpene synthases and then evaluate selected mutations using MD simulation. Interestingly, the crystal structure of SdS in complex with the substrate analogue 2,3-dihydrofarnesyl diphosphate (DHFDP) indicates that three water molecules are part of the active site contour, located between the H- and K-helices (Figure 1A). The non-hydroxylating sesquiterpene synthase producing (+)-aristolochene also has a single water molecule in the equivalent position, coordinated by the K-helix residues S303 and N299 (Figure 1B). Mutation of these residues can lead to significant formation of the linear hydroxylated products nerolidol and farnesol (47.4% for S303D, 22% for N299A + S303A).^25^ Presumably, these mutations lead to activating the water from the active site template for hydroxylation (and/or allowing for additional water to enter the active site), leading to immediate quenching of the initially formed farnesyl cation. Notably, the equivalent position to S303 in germacradien-11-ol synthase (Gd11olS) is H320, and our previous work has indicated that this residue plays a significant role in water attack at the cyclic terpene intermediate isolepidozene to form germacradien-11-ol.^22,42^ We thus hypothesized that equivalent mutations on the K-helix in SdS (A301 and G305 are equivalent to N299 and S303 in aristolochene synthase from *Aspergillus terreus*) could similarly lead to the activation of the water molecules located there, perhaps allowing cyclization to occur first. Mutating W304, also directly adjacent to this active site water cluster, may lead to opening a channel to the bulk solvent similar to the W279A mutation in δ-cadinene synthase (which leads to 89% hydroxylated product).^28^ We then used MD simulations of the FDP-complex for SdS_WT_ and selected variants at these positions (based on the structure of SdS in complex with DHFDP, PDB code 4OKZ,^34^ and the simulation protocols from our previous work;^22^ see details in the Supporting Information). These simulations can be used to indicate if mutations are likely to lead to undesired products, e.g., linear products and/or early quenching of intermediates by water. For example, our FDP-complex simulations for Gd11olS variants correctly identified two variants that formed significant amounts of linear product (W312A and H320A, forming 42 and 23% nerolidol, respectively).^22^ In addition to mutations on the K-helix (with G305D and G305E similar to S303D in aristolochene synthase and W304S possibly connecting the cluster of three active site waters with bulk solvent, similar to W279A in δ-cadinene synthase), we also simulated F297A, a mutation that may lead to an increase in linear products due to disrupting the active site contour that makes FDP fold back on itself (Figure 1A). Monitoring the C1–C10 distance (from 10 independent simulations of 30 ns for each SdS variant) indicates that cyclization is not significantly hampered by most mutations, with F297A being the possible exception, as expected (Figures 1C and S1; 26% of FDP conformations with C1–C10 distance >8 Å). To assess the influence of mutations on the water cluster observed in SdS_WT_, where changes may lead to, e.g., premature quenching of carbocations by water (leading to linear or other less complex terpene alcohols, which we intended to avoid), we monitored the proximity of water to C11 (Figure 1D). For three of the variants simulated (W304S, G305E, and W304S+G305E), this indicated some changes compared to SdS_WT_. For W304S, there is an increase in water near C11 (with at least one water molecule within 5.5 Å throughout the simulation), confirming that this mutation indeed leads to an opening to the bulk solvent. However, water proximity to C11 does not differ significantly from SdS_WT_. When G305E is introduced, two of the “active site contour” water molecules (Figure 1A) clash with the side chain of E305 and were thus removed prior to simulation. Despite this, the reduction of water near C11 is modest (Figure 1D), which, therefore, does not exclude hydroxylation. Overall, the MD simulations of the suggested K-helix variants (mutation of W304 and G305) indicate that these should allow for C1–C10 cyclization (and thus the formation of carbocation A and subsequent reaction products) and do not exclude the quenching of (cyclic) carbocations by water.

### Site-Directed Mutagenesis To Create a Hydroxylating Variant of SdS

**Figure 2:**
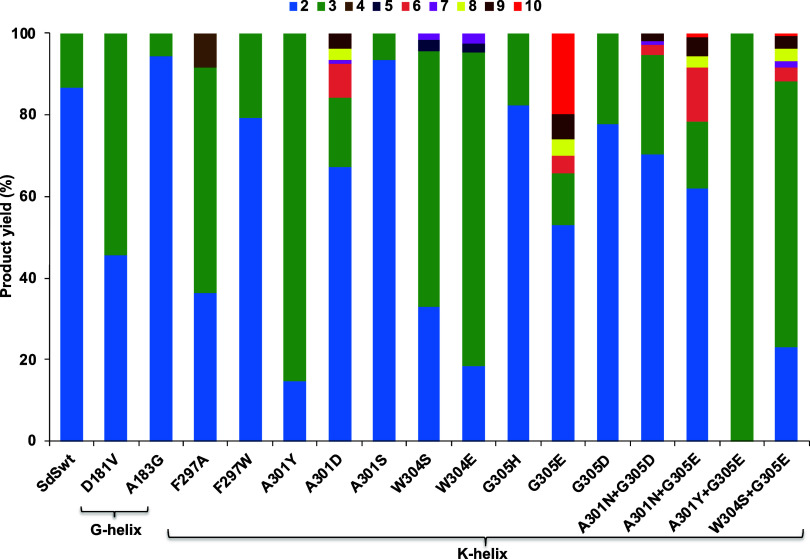
Product distributions in the pentane extracts arising from incubation of (2*E*,6*E*)-FDP (**1**) with SdS_WT_ and its variants **2:** selina-4(15),7(11)-diene, **3:** germacrene B, **4:** β-farnesene, **5:** germacrene D, **6:** δ-selinene, **7:** β-elemene, **8:** uncharacterized sesquiterpene, **9:** selina-3,7(11) diene, and **10:** selin-7(11)-en-4-ol. Total ion chromatograms for SdS_WT_ and all the variants are presented in Figures S3–S19, along with mass spectra for all 9 compounds in Figures S40–S48.

Mutation of residues in the K-helix (^301^A--^304^WG^305^) identified through comparison with previous work and our MD simulations (above) may activate water molecule(s) in close vicinity of the reaction center for quenching cyclized carbocations to produce complex hydroxylated sesquiterpenes. Site-directed mutagenesis was carried out on these selected residues aiming to increase the polarity and hence the possibility of activating water in this site (A301 to Y, S, and D; W304 to S and E; G305 to H, D, and E). The K-helix residue F297 was also investigated to observe if linear product formation might occur (as suggested by MD simulation). SdS F297A indeed led to minor formation (**4**, 8.3%) of the linear product β-farnesene (whereas SdS F297W showed a similar product distribution as SdS_WT_), confirming that F297 is involved in the substrate folding required for initial cyclization (C1–C10 ring closure). Both F297 mutations result in a significant reduction in the kinetic efficiency (through an increase in *K*_M_ as well as a decrease in *k*_cat_, see Table 1). Mutation of A301S did not significantly change the product profile (with <4-fold reduction of efficiency due to a small increase in *K*_M_, Table 1) and A301Y resulted in a switch toward germacrene B (**3**, 85.4%) as a major product with a reduced level of selina-4(15),7(11)-diene (**2**, 14.6%) (with a 5-fold reduction of efficiency, Table 1). A301D led to the formation of small quantities of alternative side products δ-selinene (**6**, 8.3%) and selina-3,7(11)-diene (**9**, 3.9%, Figure 2, Table S2) (not produced by SdS_WT_) along with selina-4(15),7(11)-diene as the major product, alongside a significantly reduced *k*_cat_ (35-fold) and increased *K*_M_ (Table 1). These results showed that replacing A301 with larger polar residues could lead to changes in the product profile, altering the carbocation rearrangements likely due to changes in the active site contour, but did not introduce water capture of carbocations.

**Table 1:** Kinetic Constants of SdS_WT_ and Its Variants^a^

	*K***_M_ (μM)**	*k***_cat_ (s**^**–1**^**) × 10**^**–3**^	*k*_**cat**_**/***K*_**M**_(μM^–1^ s^–1^) × 10^**–3**^
SdS_WT_	0.86 ± 0.11	7.0 ± 0.02	8.14
D181V	5.25 ± 0.58	13.5 ± 0.06	2.57
A183G	0.91 ± 0.26	3.6 ± 0.02	3.96
F297A	4.33 ± 2.27	1.4 ± 0.03	0.32
F297W	4.16 ± 0.78	1.73 ± 0.01	0.42
A301Y	3.22 ± 0.37	5.6 ± 0.02	1.74
A301D	6.26 ± 4.0	0.20 ± 0.01	0.03
A301S	4.23 ± 0.88	9.16 ± 0.07	2.17
W304S		ND	
W304E		ND	
G305H	0.31 ± 0.10	1.0 ± 0.06	3.23
G305E	1.80 ± 0.19	4.0 ± 0.01	2.22
G305E (pH 6.0)	1.76 ± 0.36	4.0 ± 0.02	2.27
G305D	7.42 ± 3.1	2.3 ± 0.05	0.31
A301N+G305D		ND	
A301N+G305E	1.11 ± 0.12	0.7 ± 0.002	0.63
A301Y+G305E	11.11 ± 4.0	0.5 ± 0.01	0.05
W304S+G305E		ND	

a ND: Not determined due to low activity.

G305 in the K-helix of SdS (corresponding to S303 in AS and H320 in Gd11olS) was investigated next. Although the replacement of G305 with H and D did not change the product profile, replacement with E resulted in the formation of the sesquiterpene alcohol selin-7(11)-en-4-ol (**10**, 20%), which is not produced by wild-type, along with selina-4(15),7(11)-diene as a major product (52.9%, Figure 2, Table S2). Notably, G305E caused a more significant change in the active site water cluster compared to G305D in our FDP-complex MD simulations (Figure 1D). Formation of selin-7(11)-en-4-ol (**10**) is expected to arise from the hydroxylation of carbocation B on C3, instead of deprotonation of C15 to form selina-4(15),7(11)-diene (**2**, Scheme 1). SdS W304S and SdS W304E were also created, as this is expected to increase water availability around carbocation B (see, e.g., Figure 1D), but these variants did not result in hydroxylated product formation: both variants produced germacrene B (**3**) as major product along with a reduced level of selina-4(15),7(11)-diene (**2**) (Figure 2, Table S2).

In an attempt to further increase the formation of selin-7(11)-en-4-ol observed in SdS G305E, we carried out several double mutations at A301, W304, and G305. Functional characterization of the double mutant A301Y + G305E resulted in germacrene B (**3**) as the sole product (the neutral intermediate in SdS_WT_,^23^Figure 2). However, double mutants A301N + G305E and A301N + G305D resulted in a profile largely similar to SdS_WT_, with just a higher proportion of alternative non-hydroxylated side-products (e.g., δ-selinene (**6**) in variant A301N+G305E; Figure 2, Table S2). Furthermore, the kinetic characterization of all double mutants resulted in a significant reduction in the overall kinetic efficiency (Table 1).

In Class I terpene synthases, the G-helix is structurally conserved and undergoes structural changes upon substrate binding and plays a crucial role in product distribution.^17,34,44,45^ Point mutation to the kink in the G-helix has resulted in the abolition of the hydroxylation activity in germacradien-11-ol synthase.^22^ However, in δ-cadinene synthase (DCS), site-directed saturation mutagenesis of the hinge points of the kink resulted in a variant (N403P/L405H) producing germacradien-4-ol (Gd4ol) by utilizing water capture.^46^ Hence, we generated two variants in this region, D181V and A183G (equivalent to V187 and G189 in germacradien-11-ol synthase). G182A was previously shown to lead to strongly reduced production of selina-4(15),7(11)-diene and an increase in germacrene B.^34^ Functional characterization of D181V showed a change in the product distribution, with germacrene B and selina-4(15),7(11)-diene being produced in equal proportions with a ∼3-fold reduction in kinetic efficiency (Table 1). However, variant A183G did not display any significant change in the product profile.

### Characterization of the Hydroxylated Sesquiterpene Produced by SdS G305E

**Figure 3:**
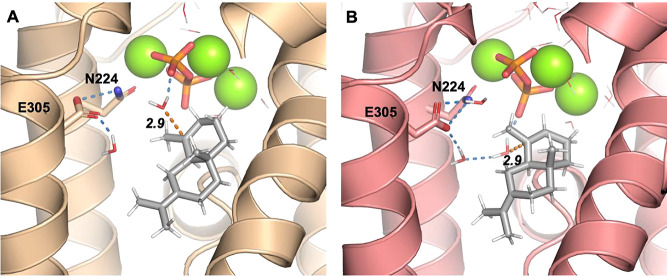
QM/MM simulations of carbocation B in the SdS G305E. (A) Example of a water approach to cationic carbon C3 in a pre-*S* position. (B) Example of a water approach to C3 in a pre-*R* position. E305 and N224 side chains are shown. C3 to water oxygen distance is indicated by the orange dashed line (with distance in Å labeled) Hydrogen bonds between E305, water and N224 are indicated by dashed blue lines. The backbone of residues 48–60 is omitted for clarity.

To determine the structure of the major sesquiterpene alcohol (**10**) produced by the G305E variant, preparative scale incubation with (2*E*,6*E*)-FDP (see the Supporting Information for details) was performed, resulting in 15 mg of the product. After chromatographic purification using deactivated silica (neutralized with 1% triethylamine) with *n*-pentane as the eluent, a total of 3.5 mg of colorless oil was isolated with >99% purity, as judged by GCMS (Figure S20). The purified compound was analyzed by NMR spectroscopy and characterized as selin-7(11)-en-4-ol (spectroscopic assignment of the ^1^H, ^13^C NMR, and 2D spectra are given in the Supporting Information, Figures S63–S68) by comparison with published NMR spectroscopic data.^35−37^ Selin-7(11)-en-4-ol (eudesm-7(11)-en-4-ol) has been isolated as a racemate from the steam-distillation of the flowering plant *Dipterocarpus cornutus* and its structure has been deduced using NMR spectroscopy and X-ray studies.^35^^1^H and ^13^C NMR spectra recorded in this study match well with those reported for the racemate but it differs for one carbon signal reported for dextrorotatory enantiomer of selin-7(11)-en-4-ol (Tables S3 and S4). Moreover, recent work has established the absolute configuration of selina-4(15),7(11)-diene as 2*S*,7*R*-selina-diene.^23^ Since selin-7(11)-en-4-ol and selina-4(15),7(11)-diene are derived from the same carbocation (Scheme 1) and produced together, it is safe to assume that their stereocenters at C2 and C7 will have the same configuration. With this information, the selin-7(11)-en-4-ol characterized here can be identified as a levorotatory enantiomer. Our detailed 2D NMR spectra analysis (Figures S65–S67) thus reveals the configuration of the stereocenter of C4 in the produced selin-7(11)-en-4-ol as *S* (C3 in FDP numbering; Scheme 1). To the best of our knowledge, a native synthase for this compound is yet to be identified. SdS G305E is thus the first sesquiterpene synthase that can be called a selin-7(11)-en-4-ol synthase and could be used to produce this molecule stereospecifically using synthetic biology tools, and its biological potential could be further explored. A first step toward such production would be to increase the selin-7(11)-en-4-ol (**10**) yield. Mechanistic details on the formation of **10** by SdS G305E may provide insights into how this might be achieved. We thus performed QM/MM (DFTB3/CHARMM36) MD simulations of SdS_WT_ and SdS G305E in complex with carbocation B, which is quenched either by deprotonation (forming **2** or **9**; Scheme 1) or by water addition (forming **10**). These simulations (10× 1 ns, with carbocation B treated with DFTB3; details in Supporting Information) indicated that in SdS_WT_ water does not approach the cationic carbon (C3) consistent with hydroxylation to form an *S* configuration, whereas it does for SdS G305E. (For SdS_WT_, 8 out of 10 independent simulations did not have water approaching within 3.4 Å; in the remaining 2 simulations, a water molecule gets “stuck” in a position with no mobility, which may be an artifact, and where hydroxylation would lead to an *R* configuration; Figure S2A). In SdS G305E high-mobility water approaching C3 was observed in all simulations (Figures 3 and S2A). Water can approach C3 from two different locations: a pre-*S* conformation where it is coordinated by PPi and sometimes also E305 (Figure 3A), or a pre-*R* conformation where it is coordinated by PPi only (Figure 3B). Only the pre-*S* position is consistent with the formation of **10** (*S* configuration at C3), instead of its enantiomer (*R* at C3). PPi is in a prime position to assist with hydroxylation, possibly aided by G305E, by abstracting a proton from the water molecule. However, the balance between such hydroxylation and the deprotonation of C15 by PPi leading to product **2** is likely to be subtle, as this deprotonation may occur (with proton abstraction distances similar between SdS_WT_ and SdS G305E, see Figure S2C), for example when there is no water molecule correctly positioned sufficiently close to C3 and PPi (Figure S2E). In SdS_WT_, no water is observed approaching C3 from the pre-*S* conformation. This is because the water cluster as observed in the crystal structure (Figure 2A) is maintained (Figure S2D), forming part of the active site contour, which prevents these water molecules from being “activated” for hydroxylation of C3.

### Increased Selin-7(11)-en-4-ol Formation through pH Optimization

**Figure 4:**
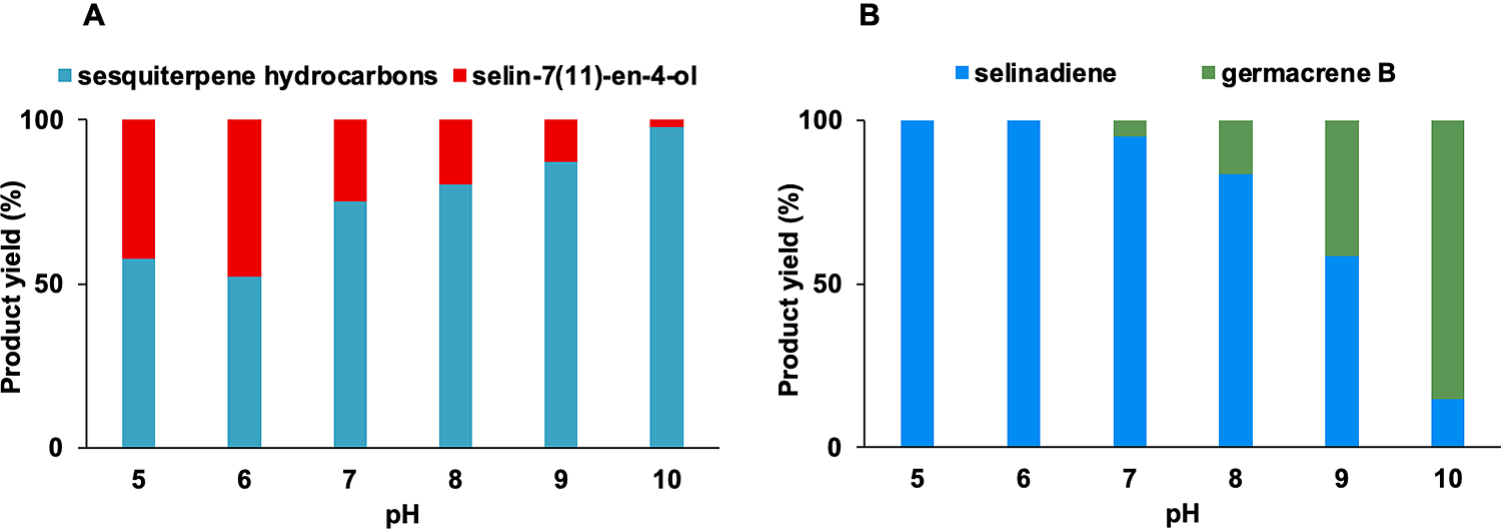
Effect of the pH on the catalysis of SdS_WT_ and variant G305E upon incubation with (2*E*,6*E*)-FDP. (A) Effect of pH on the product distribution of SdS variant G305E. (B) Effect of pH on product distribution in SdS_WT_. At lower pH, 100% selina-4(15),7(11)-diene (**2**) is formed, whereas increasing the pH leads to increasing amounts of germacrene B (**3**).

Several studies have shown that the product profile of terpene synthases can be changed by varying pH.^1,47^ PPi may be either unprotonated (as in our simulations) or singly or doubly protonated when in complex with (sesqui)terpene synthases.^48,49^ This protonation state will affect the basicity of PPi and thereby likely the efficiency of carbocation quenching (with higher efficiency at lower pH, until it affects other aspects of turnover). For SdS_WT_, the quenching would be through deprotonation (with no water molecule readily available for hydroxylation), whereas for SdS G305E, a mixture of deprotonation and hydroxylation would occur. For the latter, the balance is likely to shift with a change in pH (the efficiency of deprotonation and hydroxylation will likely differ with changes in pH). It is therefore possible that changing pH could help increase the yield of selin-7(11)-en-4-ol in SdS G305E. We thus set up enzymatic reactions in various buffers with pH ranging from 4.0 to 10.0 for SdS G305E and SdS_WT_ with (2*E*,6*E*)-FDP (Figures S21–S34). Product distributions differed significantly between pH 5.0–10.0 (Figure 4 and Table S5; no activity was detected for either variant at pH 4.0). GCMS analysis of the pentane extractable products from incubation of SdS G305E with (2*E*,6*E*)-FDP indicated an increase in selin-7(11)-en-4-ol formation with decreasing pH, the highest being 47.8% at pH 6.0, whereas sesquiterpene hydrocarbon formation was higher with an increase in pH (with germacrene B as a major product at pH 10.0). Moreover, decreasing the pH for SdS_WT_ led to exclusive selina-4(15),7(11)-diene formation at pH 5.0 and 6.0. Conversely, the proportion of germacrene B formation increased with a higher pH, becoming the major product at pH 10.0 (Figure 4, Table S5).

This change in the product profile due to lower pH may help progress the reaction past germacrene B in both wild-type and G305E by favoring the reprotonation of germacrene B that is required to produce carbocation B (Scheme 1), whereas at higher pH, the reprotonation of germacrene B is hindered, resulting in germacrene B as a major product. This subtle change in reprotonation efficiency would be consistent with the proposed Gly182 backbone carbonyl as the base/acid in this process.^23^ There was no selin-7(11)-en-4-ol formation observed in SdS_WT_ at lower pH (100% selina-4(15),7(11)-diene formed at pH 6.0), indicating that the formation of this compound by SdS G305E is due to the presence of glutamic acid rather than the pH change itself. The effect of G303E on hydroxylation is subtle: once the reaction has progressed past germacrene B, the proportion of the major product selin-7(11)-en-4-ol formed through hydroxylation (**10**, 48%) and that formed through proton abstraction (**2** and **9**, together 47%) is approximately equal at pH 6.0 (Table S5). This points to a finely balanced situation, with highly similar free energy barriers for these reactions in SdS G303E. However, as expected for sesquiterpene synthases, the overall reaction is likely limited by product release.^50^ Kinetic characterization of SdS G305E at pH 8.0 and 6.0 (which produces 20 and 47.8% selin-7(11)-en-4-ol, respectively) indicated similar kinetic parameters (Table 1) with a four-fold reduction in overall kinetic efficiency as compared to SdS_WT_ (Table 1). G305E thus likely leads to slightly slower product release, and this is not significantly affected by pH. Overall, these results show that engineering water capture in selina-4(15),7(11)-diene synthase led to the creation of a novel variant (which can be called a selin-7(11)-en-4-ol synthase) with broad pH stability.

### Metabolic Engineering for Bacterial Production of Selin-7(11)-en-4-ol (**10**)

**Figure 5:**
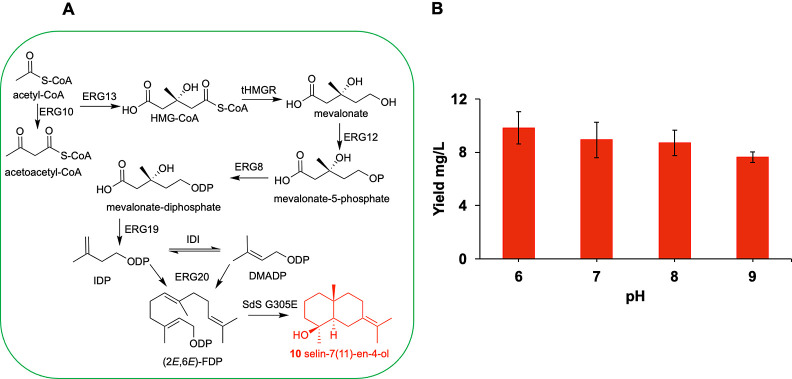
Production of selin-7(11)-en-4-ol in bacterial strains using engineered BL21(DE3) cells. (A) Schematic representation of enzymatic steps of the mevalonate pathway and SdS G305E catalyzing conversion of acetyl co-A into selin-7(11)-en-4-ol. ERG10: acetoacetyl-CoA thiolase from *Escherichia coli*, ERG13: HMG-CoA synthase from *Saccharomyces cerevisiae*, tHMGR: truncated-HMG-CoA reductase from *S. cerevisiae*, ERG12: mevalonate kinase from *S. cerevisiae*, ERG8: phosphomevalonate kinase from *S. cerevisiae*, ERG19: mevalonate-diphosphate decarboxylase from *S. cerevisiae*, IDI: IDP isomerase and ERG20: (2*E*,6*E*)-farnesyl diphosphate synthase from *E. coli* and SdS G305E: selina-4(15),7(11)-diene variant G305E producing selin-7(11)-en-4-ol. (B) Level of selin-7(11)-en-4-ol produced in batch fermentation at different pH (6.0–9.0) after 24 h of induction by the SdS G305E variant.

Metabolic engineering strategies have been applied for terpene production in bacteria and yeast strains by engineering extra copies of mevalonate pathway genes, upregulating the intrinsic MEP pathway genes, and downregulating competing routes downstream of GDP, FDP and/or GGDP depending on the final outcome.^51−55^ We exploited a similar approach to demonstrate the production of selin-7(11)-en-4-ol (**10**) by incorporating mevalonate pathway genes cloned in vectors (pMevT and PMBIS, see Supporting Information for details) and SdS G305E (Figure 5A). Our pH optimization studies showed that, in an in vitro reaction condition, the formation of selin-7(11)-en-4-ol increased at a slightly lower pH (6.0), with it becoming the major product. We carried out test fermentation reactions at different pH (6.0–9.0) to see the effect on the formation of selin-7(11)-en-4-ol in an in vivo environment. Analysis of the dodecane layer after 24 h induction resulted in selin-7(11)-en-4-ol (17%) at pH 6.0 with selina-4(15),7(11)-diene (**2**) as a major product. By increasing the pH (7.0–9.0) of culture broth, the proportion of selin-7(11)-en-4-ol decreased, accompanied by an increase in the formation of selina-3,7(11)-diene (**9**) (Figures S35–S38). Quantitative analysis using GC showed that selin-7(11)-en-4-ol production was achieved on the scale of 9.8 ± 1.2 mg/L of bacterial culture at pH 6.0 (Figure 5B).

## Conclusions

In this study, we have used a combination of molecular dynamics simulation and site-directed mutagenesis to introduce water capture in non-hydroxylating selina-4(15),7(11)-diene synthase (SdS). We have identified that mutation of G305 in the K-helix region can activate water in the active site cavity. Introducing G305E resulted in the formation of a complex hydroxylated sesquiterpene alcohol that we characterized as selin-7(11)-en-4-ol using NMR spectroscopy. Simulation indicated that this mutation leads to water becoming available to quench the final carbocation. Based on this, optimizing the pH in the enzyme assay of SdS G305E led to a significant increase in the formation of selin-7(11)-en-4-ol. To the best of our knowledge, this is the first report on the enzymatic formation of selin-7(11)-en-4-ol. We also incorporated the SdS G305E variant along with the mevalonate pathway genes in bacterial strains and demonstrated the in vivo formation of selin-7(11)-en-4-ol. This study may open the way for engineering terpene synthases by introducing hydroxylation to create new enzymes that can be applied to the sustainable production of bioactive molecules.

## Methods

Materials and methods used are described in detail in the Supporting Information.

## Data Availability

Raw data for GC-MS analysis, product ratio calculations, mass fragmentation, NMR spectroscopic data files for selin-7(11)-en-4-ol, and kinetic raw data for SdS_WT_ and mutants are available at https://doi.org/10.17035/d.2024.0307793923.

## Supporting Information Available

Details on cloning, mutagenesis, expression and purification of protein variants; MD and QM/MM simulation methods and results (Figures S1–S2); GC-MS chromatograms (Figures S3–S34), GC chromatograms (Figures S35–S39), mass fragmentation patterns (Figures S40–S48), kinetics and product analysis (including NMR spectra; Figures S49–S68); are described in the Supporting Information document (PDF)
Relevant simulation files provided in sds_md.zip (ZIP)

## Abbreviations

MD: molecular dynamics
SdS: selina-4(15),7(11)-diene synthase
NMR: nuclear magnetic resonance
