# Supplementary material for: Simulation-Guided Engineering Enables a Functional Switch in Selinadiene Synthase toward Hydroxylation
Source: ACS Catal. 2024 Jul 9;14(14):11034–43. doi: 10.1021/acscatal.4c02032 (PMC11264211; doi:10.1021/acscatal.4c02032)
Supplement: Supplementary file 1 — cs4c02032_si_001.pdf [file cs4c02032_si_001.pdf]

Supporting Information

Simulation-guided engineering enables a functional switch in selinadiene synthase towards hydroxylation

Prabhakar L. Srivastava,[†] Sam T. Johns,[‡] Angus Voice,[‡] Katharine Morley,[‡] Andrés M. Escorcia,[‡] David J. Miller,[†] Rudolf K. Allemann*[†] and Marc W. Van der Kamp*[‡]

[†]School of Chemistry, Cardiff University, Main Building, Park Place, Cardiff CF10 3AT, United Kingdom

[‡]School of Biochemistry, University of Bristol, University Walk, Bristol BS8 1TD, United Kingdom

*Correspondence to: marc.vanderkamp@bristol.ac.uk, allemannrk@cardiff.ac.uk

Table of Contents

1.	General materials and methods	S2
2.	Construction of SdS variants	S3
3.	Expression and purification of SdS_{WT} and variants	S3
4.	Enzymatic incubations and product analysis	S4
5.	Kinetic characterization of SdS_{WT} and variants	S4
6.	Large scale incubations and product characterization	S5
7.	pH optimisation for increased selin-7(11)-en-4-ol formation	S6
8.	*In-vivo* production of selin-7(11)-en-4-ol using metabolic engineering	S6
9.	Computational methods	S8
10.	Tables	S11
11.	Analysis of MD simulations	S16
12.	Total ion chromatograms (TICs)	S18
13.	GCMS Mass Spectra	S37
14.	Kinetic data	S42
15.	NMR spectra	S49
16.	References	S54

1. General materials and methods

The full-length gene sequence (1-365 aa) coding for selina-4(15),7(11)-diene synthase (SdS, UniProt ID: B5HDJ6) from *Streptomyces pristinaespiralis* ATCC 25486[1] was codon optimised for *E. coli* and synthesized in a pET28a vector frame between EcoR1 and HindIII restriction site from Genscript with an N-terminal His tag.

A prestained protein size marker (14.4-116.0) kDa was used to identify proteins by 12% SDS-PAGE. PrimeSTAR® master mix was purchased from TaKaRa. Primers for mutagenesis were purchased from Sigma Aldrich (UK). All mutated constructs were confirmed by DNA sequence analysis from Eurofins. [1-^{3}H]-FDP (20 Ci/mmol) was purchased from American Radiolabelled chemicals, Inc. Commercial [1-^{3}H]-FDP was diluted by adding unlabelled FDP to give a final specific activity of 24000 dpm/µM.

^{1}H and ^{13}C NMR spectra were measured on a Bruker Ultrashield 500 NMR spectrometer and are reported as chemical shifts in parts per million downfield from tetramethylsilane.

Commercial (*E*)-nerolidol was used as an authentic standard for gas chromatography. (*E*,*E*)-FDP was synthesized in our lab as described previously.[2]

Gas chromatography coupled with mass spectrum (GC-MS) was performed on a Perkin Elmer Clarus 680 GC fitted with a Perkin Elmer Elite-1 column 100% dimethyl polysiloxane (30 m x 0.25 mm internal diameter) and a Perkin Elmer Clarus SQ 8 C mass spectrometer: The elution program used an injection port temperature of 100 °C; split ratio 19:1; initial temperature 80 °C hold 2 min, ramp of 8 °C/min to 280 °C and hold for 3 min with a flow rate of 1 mL He/min. GC analysis to quantify selin-7(11)-en-4-ol formation from the overproduction using mevalonate pathway genes in BL21(DE3) cells was performed on Agilent 7890A GC system equipped with a RestekRt-BDEXsm silica capillary column (30 m x 0.32 mm internal diameter) on

following parameter: injection port at 100 °C; split ratio 5:1; initial pressure 1 kPa; initial column temperature 80 °C, hold 2 min, ramp of 8 °C/min to 220 °C (3 min hold), flow 1 mL H/min.

Thin layer chromatography was performed on pre-coated aluminium sheets of Merck silica gel 60 F254 (0.20 mm). TLC visualizations were performed by staining the TLC with anisaldehyde.

2. Construction of SdS variants

Mutation primers were designed for the selected amino acid present in the active site pocket (Table S1). For PCR the following mixture was used: 12.5 µL PrimeSTAR® master mix, 0.5 µL forward/ reverse primer (10 µM stock), 50 ng template DNA (plasmid), 1 µL DMSO and water to a final volume of 25 µL. After the PCR, 1 µL of DpnI (NEB) was added to each reaction mixture and further incubated at 37 °C for 2 h. A 5 µL of samples were transformed into XL1 blue chemical competent cells and plated on LB kanamycin agar plates and incubated at 37 °C for overnight to select the positive clones. Single colonies were inoculated in LB medium containing kanamycin (50 µg/mL) and incubated at 37 °C for overnight. Plasmids were isolated using a plasmid miniprep kit (Qiagen). The variant sequences were verified via sequencing using the forward (T7 promoter) or the reverse primer (T7 terminator).

3. Expression and purification of SdS_{WT} and variants

pET28a harbouring SdS_{WT} and variants were introduced in BL21(DE3) chemically competent cells for protein expression and positive clones were selected on LA + kanamycin (50 µg/mL) plates. A single colony was used to inoculate 20 mL of LB media containing 50 µg/mL of kanamycin and grown overnight at 37 ºC and 200 rpm.

The overnight grown culture was transferred to 500 mL of terrific broth (TB) media containing 50 μg/mL of kanamycin and incubated at 37 ºC and 200 rpm until the OD at 600 nm reached 0.8-1.0. At this stage, cultures were induced by addition of isopropyl β-D-1-thiogalactopyranoside (IPTG, a final concentration of 0.2 mM) and incubated overnight at 16 °C and 200 rpm. The cells were harvested by centrifugation at 6000*g* for 20 min and pellets were stored in -20 ºC until further use. The induced pellets were resuspended in 20 mL lysis buffer /g of cell mass (20 mM Tris, pH 8.0, 500 mM NaCl, 10 mM $MgCl_2$, 10% glycerol, 1 mg/mL lysozyme and 1 mM PMSF). The cells were lysed by sonication (amplitude 40%, pulse on 5 s and pulse off 10 s for 5 min) and the resulting supernatant was centrifuged at 18000*g* for 45 min to remove the cell debris. The supernatant was filtered through 0.2 μm syringe filters and applied to a Ni-NTA affinity drip column (QIAGEN, 5 mL). Proteins were eluted with 2 column volumes of 20 - 250 mM imidazole gradient in lysis buffer. Fractions containing proteins with purity >95% as judged by SDS-PAGE were combined and dialyzed against 20 mM Tris, pH 8.0, 100 mM NaCl, glycerol 10%. The Bradford protein assay[3] was used to measure the concentration of total protein and commercial bovine serum albumin as the calibration standard.

4. Enzymatic incubations and product analysis

Enzymatic assays were performed using 5 μM of purified protein in 50 mM Tris buffer, pH 8.0, containing 5 mM $MgCl_2$, 5.0 mM 2-mercaptoethanol and 100 μM FDP in a 500 μL scale overlaid with 1 mL of n-pentane, incubated for overnight at room temperature with gentle agitation. After the incubation, assay mixtures were vortexed and the n-pentane layer was transferred to fresh vial. The pentane extracts were analyzed by GC-

MS using the method described above. The products formed were verified by NIST Library Mass Spectra matches and NMR spectra analysis.

5. Kinetic characterization of SdS_{WT} and variants

Steady-state kinetics assays were carried out using [1-^{3}H]-(*E*,*E*)-FDP (240000 dpm nmol^{-1}) in buffer as previously described.[4] Briefly, reactions (250 µL) were initiated by addition of purified enzyme (100 nM) to assay buffer solutions containing [1-^{3}H]-(*E*,*E*)-FDP (0.02-20 µM) at 0 °C and overlaid with 1 mL of hexane. The resulting enzymatic preparations were incubated at 30 °C for 10 minutes, cooled on ice and immediately quenched by addition of EDTA (50 µL, 0.5 M) and vortexing for 30 s. The organic layer was then passed through a small silica column (~500 mg) into 15 mL EcoScintTM fluid (National Diagnostics), the aqueous portion was further extracted twice with 1 mL portions of 11:1 hexane/diethyl ether by vortexing for 10 s and the organic extracts were passed through the same silica column into the 15 mL EcoScintTM fluid. At the end the silica column was washed with 1 mL of 11:1 hexane/diethyl ether. The combined organic extracts in EcoScintTM fluid was analyzed on a scintillation counter (Packard 2500 TR™) in ^{3}H mode for 4 min per sample. The kinetic constants (K_M and k_{cat}) were calculated by fitting the data to the Michaelis-Menten equation, using Systat Sigmaplot.

6. Large scale incubations and product characterization

For the characterization of hydroxylated sesquiterpene produced from SdS G305E, preparative scale incubations were carried out using 10 µM of purified protein with 0.35 mM FDP in Tris-HCl buffer, pH 8.0, 5 mM $MgCl_2$ and 5 mM 2-mercaptoethanol in a total reaction volume of 100 mL. This solution was overlaid with 100 mL of n-

pentane and incubated at room temperature for 24 h with gentle stirring. Reactions were performed in duplicates. After incubation, all the reaction mixtures were pooled, extracted twice with 100mL n-pentane each time, and the n-pentane solution was concentrated and analyzed by GC-MS.

In order to purify the hydroxylated sesquiterpene from the crude reaction mixture, a filter column was prepared (5 cm x 0.5 cm) using triethylamine treated (1% in pentane) silica gel (230-400 mesh particle size). The crude product was loaded onto the column and eluted with n-pentane and ethyl acetate gradient, collecting 5 mL fractions. All the fractions were analyzed by GC-MS as described above. The sesquiterpene alcohol was eluted in the 5% pentane and ethyl acetate solution. Eluted fractions were pooled together before being dried over anhydrous Na_2SO_4. The resulting solution was filtered, and solvent removed carefully under reduced pressure (300 mbar, 30 °C water bath) to give 3.5 mg of colourless oil as judged by taking weight using analytical balance. For NMR spectroscopic analysis the sample was dissolved in $CDCl_3$ and analyzed by ^1H, ^{13}C, DEPT, HSQC, HMBC, NOSEY and COSY NMR spectroscopy.

7. pH optimisation for increased selin-7(11)-en-4-ol formation

Enzymatic assays were performed in different pH to improve the formation of selin-7(11)-en-4-ol by G305E using 5 µM of purified protein in 50 mM different buffers (Na-citrate pH 4.0, Na-acetate pH 5.0, MES pH 6.0, HEPES pH 7.0, Tris pH 8.0, CHES pH 9.0 and 10.0) containing 5 mM $MgCl_2$, 5.0 mM 2-mercaptoethanol and 100 µM FDP in a 500 µL scale. The reaction mixtures were overlaid with 1 mL of n-pentane and incubated at room temperature for overnight with gentle agitation. After the incubation, assay samples were vortexed, and the n-pentane layer was transferred to fresh vial. The

pentane extracts were analyzed by GC-MS to determine the product distribution using the method described above.

8. *In-vivo* production of selin-7(11)-en-4-ol using metabolic engineering

In order to overproduce the selin-7(11)-en-4-ol using metabolic engineering as a sustainable tool, we used similar strategies as developed earlier.[5] We purchased the pMevT and pMBIS plasmids from Addgene containing entire mevalonate pathway genes to overproduce (2*E*,6*E*)-farnesyldiphosphate (FDP) which is the precursor for sesquiterpene biosynthesis. Both these plasmids were incorporated in *E. coli* BL21(DE3) cells along with pET28a vector containing SdS G305E variant. Positive clones were selected by growing the transformation mixture on LB media containing antibiotics kanamycin + chloramphenicol + tetracycline. Positive clones containing all the plasmids (pMevT, pMBIS and pET28 SdS G305E) were inoculated in LB media containing kanamycin + chloramphenicol + tetracycline as antibiotics and incubated at 37 °C for overnight at 200rpm. Overnight grown culture was transferred to 25 mL of terrific broth (TB media) containing kanamycin + chloramphenicol + tetracycline antibiotics and cultures were allowed to grow to the OD at 600 nm of 2-3. After the growth, cultures were centrifuged at 4000g for 10 minutes at 4 °C. Supernatant was discarded and pellets were suspended in 25 mL fresh terrific broth preadjusted at different pH (6.0 to 9.0), induced with 1mM final concentration of IPTG, and further incubated at 20 °C for the overproduction of selin-7(11)-en-4-ol for 24hr. 10% V/V of dodecane was added to the media to trap the products formed. After 24hr of induction, cultures were centrifuged and dodecane layer was transferred to fresh vial. A 20 μL of dodecane layer was diluted to 1 mL in pentane and analysed by GC and GCMS. To

quantify the production of selin-7(11)-en-4-ol in the fermentation broth, a calibration curve was prepared for authentic standard (*E*)-β-farnesene by injecting 5 μL of different concentration ranging from 7.8 ng/μL to 500 ng/μL in GC to calculate the peak area which was plotted against concentration. The peak area for selin-7(11)-en-4-ol at different pH was calculated and compared with the calibration curve to get the quantity produced in batch fermentation.

9. Computational Methods

Preparation and MD simulation of SdS•FDP complexes.

The crystal structure of SdS in complex with Mg^{2+} and dihydrofarnesyl pyrophosphate (PDB code 4OKZ, chain A)[1] was used as starting structure to model the Michaelis complex of SdS and its mutants (F297A, W304S, G305D, G305E and W304S/G305E) with FDP. The protein structure was submitted to the PDB2PQR server[6–8] to determine likely protonation states, His tautomers, and for checking the orientation of the side chains of Asn, Gln, and His. Protonation states were assigned based on pK_a values predicted by PropKa3.0 (as a module of PDB2PQR) and PropKa3.1 (which handles the influence of Mg^{2+} ions and farnesyl diphosphate, FDP).[9,10] All Asp and Glu side-chains were negatively charged, Lys and Arg positively charged. Histidine residues (all solvent-exposed) were either singly protonated on Nε2 (positions 19, 230 and 292) or singly protonated on Nδ1 (positions 22, 85, 93, 103, 162 and 195), based on the predicted hydrogen-bonding network. Because the natural N- and C-terminal residues were not resolved in the crystal structure, the present N- and C-terminal residues (Glu4, Ala349) were neutralized with acetyl and N-methyl amide groups, respectively. FDP was modelled in guided by the dihydrofarnesyl pyrophosphate coordinates in the crystal structure, ensuring consistency with either *R*- or *S*- germacrenyl cation formation (upon ionization and C1-C10 cyclisation). These pre-*R* and pre-*S* conformations of FDP were then subjected to geometry optimization, performing 1000 steps of steepest descent (SD) followed by 1000 steps of adapted-basis Newton–Raphson minimization (ABNR), keeping all other heavy atoms fixed. The complexes of the SdS mutants were built from their equivalent SdS_{WT} complexes using the mutagenesis wizard of the PyMOL package, with deletion of the crystallographic water HOH 1010 when introducing G305D and G305E (due to a significant steric clash). For all complexes,

FDP was then again optimized along with the crystal waters and the protein residues within 12 Å of the FDP:C1 atom. These optimizations of the FDP•enzyme complexes were performed with the CHARMM program (version 41b1).[11]

After the initial optimization, all complexes were solvated using a rectangular box of TIP3P water molecules with a minimum buffer of 13 Å around the protein, using the solvate plugin of the VMD package.[12] Using the autoionize plugin of VMD, 15 (WT, F297A, W304S) or 16 Na^+ (other mutants) ions were added to neutralize the systems.[12] Subsequently, the same minimization, heating, equilibration and production protocols were performed as in our previous works featuring FDP-sesquiterpene synthase complexes,[13,14] using the Amber18 program. To ensure Mg^{2+} ions maintained the coordination observed in the crystal structure, the same distance restraints as applied previously were again used: a one-sided harmonic restraint of 50 kcal mol^{-1} Å^{-2} was applied on distances of > 2.2 Å for the interatomic distances $Mg^{2+}{}_A$-O13, $Mg^{2+}{}_A$-O23, and $Mg^{2+}{}_C$-O13. A total of 10 independent MD simulations of 30 ns each were performed in the NPT ensemble for each SdS variant by using different initial velocity distributions. Six runs were performed using the complex corresponding to FDP in pro-*R* configuration as starting structure, and the other four MDs with the complex with FDP in pro-*S* configuration. Throughout, the CHARMM36 protein force field was employed, [15] with FDP and Mg^{2+} parameters taken from Van der Kamp *et al.*[16] and Allner *et al.*[17], respectively, and the TIP3P model for water. Periodic boundary conditions and a time step of 2 fs were used, with a direct space cut-off of 8 Å for nonbonded interactions with PME for long-range electrostatics. All bonds involving hydrogen atoms were constrained by SHAKE. Analysis of the simulations was performed using the final 20 ns of each simulation.

Preparation and QM/MM MD simulation of SdS•carbocation B complexes.

For the preparation of the QM/MM MD simulations of carbocation B in SdS_{WT} and SdS G305E, systems were prepared using the CHARMM-GUI input generator (https://www.charmm-gui.org/),[18] with CGenFF parameters[19–21] obtained for carbocation B and PPi and an otherwise similar setup (with CHARMM36 for protein and Na^+, Mg^{2+} ions and TIP3P water). All His residues (which are all solvent-exposed in SdS) were singly protonated on Nδ1. Optimized coordinates for carbocation B were obtained from Das et al. (cation D in this work)[22] and the initial position of this carbocation was modelled by aligning equivalent atoms to the conformation of dihydrofarnesyl diphosphate in the crystal structure and ensuring consistency with Wang et al.[23] (by visual inspection). In all minimization and MD simulation steps, carbocation B was then treated quantum mechanically, using the semiempirical DFTB3 method (as implemented in the sander program of AmberTools18). The protocols used were essentially the same as the MM MD simulation protocols used for the FDP•SdS complexes, with the following changes in simulation times: heating in 20 ps, NPT equilibration in 25 ps, gradual release of CA positional restraints in 20 ps. Then, 1.2 ns production was performed, with only the final 1 ns used for analysis. Again, 10 independent MD simulations were run for each SdS variant.

10. Tables

Table S1: Primer sequences for mutation. Nucleotide sequences changed are marked bold and underlined.

Sr No	Name	Forward Primer	Reverse Primer
**3**	**SdS D181V**	GTCTGTAT**GTC**GGCGCGACCAGCGTGG	GTCGCGCC**GAC**ATACAGACGCATCAG
**4**	**SdS A183G**	GACGGC**GGC**ACCAGCGTGGTTCTGCCGATGC	GCTGGT**GCC**GCCGTCATACAGACGCATCAGGG
**5**	**SdS F297A**	GCGTTGC**GCT**ATCCGTGGTGCGCAGG	CACGGAT**AGC**GCAACGCAGGCTGTGC
**6**	**SdS F297W**	GCGTTGC**TGG**ATCCGTGGTGCGCAGG	CACGGAT**CCA**GCAACGCAGGCTGTGC
**7**	**SdS A301Y**	CGTGGT**TAT**CAGGACTGGGGCATTAGCAGCG	GTCCTG**ATA**ACCACGGATAAAGCAACGCAGGC
**8**	**SdS A301D**	CGTGGT**GAT**CAGGACTGGGGCATTAGCAGCG	GTCCTG**ATC**ACCACGGATAAAGCAACGCAGGC
**9**	**SdS A301S**	CGTGGT**TCG**CAGGACTGGGGCATTAGCAGCG	GTCCTG**CGA**ACCACGGATAAAGCAACGCAGGC
**10**	**SdS W304S**	CAGGAC**TCG**GGCATTAGCAGCGTGCG	AATGCC**CGA**GTCCTGCGCACCACGGATAAAGC
**11**	**SdS W304E**	CAGGAC**GAG**GGCATTAGCAGCGTGCG	AATGCC**CTC**GTCCTGCGCACCACGGATAAAGC
**12**	**SdS G305H**	GACTGG**CAC**ATTAGCAGCGTGCGTTATACCAC	GCTAAT**GTG**CCAGTCCTGCGCACCACGG
13	**SdS G305E**	GACTGG**GAG**ATTAGCAGCGTGCGTTATACCAC	GCTAAT**CTC**CCAGTCCTGCGCACCACGG
14	**SdS G305D**	GACTGG**GAT**ATTAGCAGCGTGCGTTATACCAC	GCTAAT**ATC**CCAGTCCTGCGCACCACGG
15	**SdS A301N+ G305D**	CGTGGT**AAT**CAGGACTGG**GAC**ATTAGCAGCGTGCG	GCTAAT**GTC**CCAGTCCTG**ATT**ACCACGGATAAAGC
**16**	**SdS A301N+ G305E**	CGTGGT**AAT**CAGGACTGG**GAG**ATTAGCAGCGTGCG	GCTAAT**CTC**CCAGTCCTG**ATT**ACCACGGATAAAGC
**17**	**SdS W304S+ G305E**	CAGGAC**TCGGAG**ATTAGCAGCGTGCG	GCTAAT**CTCCGA**GTCCTGCGCACCACGGATAAAGC
**18**	**SdS A301Y+ G305E**	CGTGGT**TAT**CAGGACTGG**GAG**ATTAGCAGCGTGCG	GCTAAT**CTC**CCAGTCCTG**ATA**ACCACGGATAAAGC

Table S2: Product ratios table of SdS_{WT} and variants upon incubation with (2*E*,6*E*)-FDP (**1**), **2:** selina-4(15),7(11)-diene, **3:** germacrene B, **4:** β-farnesene, **5:** germacrene D, **6:** δ-selinene, **7:** α-elemene, **8:** uncharacterized sesquiterpene, **9:** selina-3,7(11) diene, **10:** selin-7(11)-en-4-ol.

**Retention time**	**13.06**	**13.38**	**11.8**	**12.28**	**12.37**	**12.63**	**12.88**	**13.2**	**15.17**
	**2**	**3**	**4**	**5**	**6**	**7**	**8**	**9**	**10**
**SdS_{WT}**	86.7	13.3	--	--	--	--	--	--	--
**D181V**	45.5	54.5	--	--	--	--	--	--	--
**A183G**	94.5	5.5	--	--	--	--	--	--	--
**F297A**	36.4	55.4	8.3	--	--	--	--	--	--
**F297W**	79.2	20.8	--	--	--	--	--	--	--
**A301Y**	14.6	85.4	--	--	--	--	--	--	--
**A301D**	67.3	17.1	--	--	8.3	0.8	2.7	3.9	--
**A301S**	93.5	6.5	--	--	--	--	--	--	--
**W304S**	33.0	62.6	--	2.7	--	1.7	--	--	
**W304E**	18.5	76.9	--	2.2	--	2.4	--	--	--
**G305H**	82.5	17.5	--	--	--	--	--	--	
**G305E**	52.9	12.9	--	--	4.4	--	3.8	6.3	19.8
**G305D**	77.7	22.3	--	--	--	--	--	--	--
**A301N+G305D**	70.5	24.3	--	--	2.5	1.0	--	1.8	--
**A301N+G305E**	62.0	16.3	--	--	13.3	--	2.8	4.7	0.9
**A301Y+G305E**	--	100.0	--	--	--	--	--	--	--
**W304S+G305E**	23.1	65.2	--	--	3.3	1.6	3.0	3.2	0.7

Table S3: Comparative ^{1}H NMR analysis to characterize selin-7(11)-en-4-ol (**10**):

^{1}H NMR reported by Dachriyanus et al.[24]<br>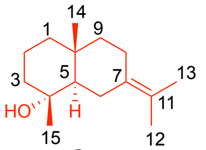<br>a<br>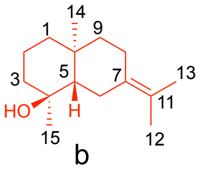<br>b	^{1}H NMR observed in this study<br>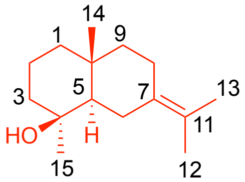	^{1}H for Compound I by Bohlmann et al.[25]<br>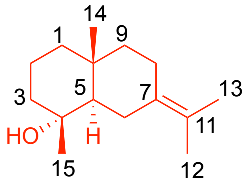<br>a	^{1}H for Compound III reported by Zhao et al.[26]<br>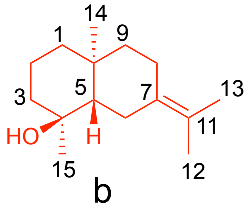<br>b
**^{1}H NMR (500 MHz, CDC13):**<br>δ 0.96 (3H, s, 14-H),<br>1.05 (1H, m, 1-H),<br>1.10 (1H, dd, J = 14.9 and 5.5 Hz, 9-H),<br>1.13 (3H, d, J = 0.8 Hz, 15-H),<br>1.14 (1H, dd, J = 13.0 and 3.0 Hz, 5-H),<br>1.31 (1H, m, 3-H),<br>1.39 (1H, m, 1-H),<br>1.42 (1H, m, 9-H),<br>1.55 (2H, m, 2-H),<br>1.64 (1H, m, 6-H),<br>1.66 (3H, q, J = 1.1 Hz, 12-H or 13-H),<br>1.69 (3H, q, J = 1.1 Hz, 12-H or 13-H),<br>1.80 (1H, dddd, J = 12.5, 3.3, 3.3 and 1.7 Hz, 3-H),<br>1.89 (1H, dd, J = 14.2 and 14.2 Hz, 8-H),<br>2.49 (1H, ddddd, J = 14.5, 2.5, 2.5, 2.5 and 2.5 Hz, 8-H),<br>2.81 (1H, ddd, J = 13.4, 2.2 and 13 2.2 Hz, 6-H).	**^{1}H NMR (500 MHz, CDC13):**<br>δ 0.96 (3H, s, 14-H),<br>1.05 (1H, m, 1-H),<br>1.10 (1H, dd, 9-H),<br>1.13 (3H, d, 15-H),<br>1.14 (1H, dd, 5-H),<br>1.31 (1H, m, 3-H),<br>1.39 (1H, m, 1-H),<br>1.42 (1H, m, 9-H),<br>1.55 (2H, m, 2-H),<br>1.64 (1H, m, 6-H),<br>1.66 (3H, q, 12-H),<br>1.69 (3H, q, 12-H),<br>1.80 (1H, dddd, 3-H),<br>1.89 (1H, dd, 8-H),<br>2.49 (1H, ddddd, 8-H),<br>2.81 (1H, ddd, 6-H).	**^{1}H NMR (500 MHz, CDC13):**<br>δ 0.95 (s, 14-H)<br>1.13 (s, 15-H)<br>1.60 (m, 6-H)<br>1.66 (s, br, 13-H)<br>1.69 (s, br, 12-H)<br>2.39 (dd, br, 8-H)<br>2.49 (dddd, 8-H)<br>2.81 (ddd, 6-H)	**^{1}H NMR (500 MHz, CDC13):**<br>δ 0.96 (d, 0.8, 14-H)<br>1.01 (ddd, 12.0, 13.0, 1.0, 9-H),<br>1.05 (m, 1-H),<br>1.07 (d, 0.6, 15-H)<br>1.15 (dd, 13.0, 2.7, 5-H),<br>1.32 (m, 1-H),<br>1.38 (m, 2-H),<br>1.39 (m, 3-H),<br>1.52 (m, 2-H),<br>1.55 (ddd, 13.0, 4.0, 1.0, 9-H)<br>1.57 (br dd 13.5, 10.4, 6-H),<br>1.62 (q, 1.6, 12-H)<br>1.64 (q, 1.6, 13-H)<br>1.69 (m, 3-H),<br>1.88 (t, 13.8, 8-H),<br>2.48 (dddd, 13.8, 12.0, 2.6, 2.6, 8-H),<br>2.92 (ddd, 13.5, 2.6, 2.6, 6-H),

Table S4: Comparative ^{13}C NMR analysis to characterize selin-7(11)-en-4-ol (**10**):

^{13}C NMR reported by Dachriyanus et al.[24] 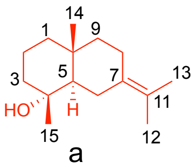 a 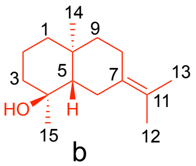 b	^{13}C NMR observed in this study 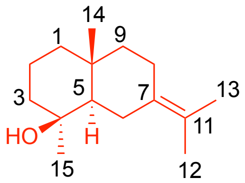	^{13}C NMR for Compound reported by Zhao et al.[26] 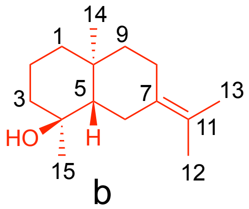 b
**^{13}C NMR (125 MHz, CDC13):**<br><br>δ 18.07 (C-14),<br>20.02 (C-12 or C-13),<br>20.07 (C-12 or C-13),<br>20.19 (C-2),<br>22.04 (C-15),<br>24.61 (C-6),<br>25.44 (C-8),<br>34.81 (C-10),<br>40.97 (C-1),<br>43.56 (C-3),<br>45.23 (C-9),<br>55.73 (C-5),<br>72.31 (C-4),<br>120.96 (C-11),<br>131.89 (C-7)	**^{13}C NMR (125 MHz, CDC13):**<br>δ 18.23 (C-14),<br>20.18 (C-12 or C-13),<br>20.23 (C-12 or C-13),<br>20.34 (C-2),<br>22.17 (C-15),<br>24.75 (C-6),<br>25.59 (C-8),<br>34.96 (C-10),<br>41.09 (C-1),<br>43.70 (C-3),<br>45.38 (C-9),<br>55.88 (C-5),<br>72.49 (C-4),<br>121.14 (C-11),<br>131.54 (C-7)	**^{13}C NMR (125 MHz, CDC13):**<br><br>δ 18.5 (C14)<br>20.1 (C12)<br>20.1 (C13)<br>20.80 (C2)<br>22.6 (C15)<br>25.3 (C8)<br>26.2 (C6)<br>35.3 (C10)<br>42.0 (C1)<br>44.1 (C3)<br>56.2 (C5)<br>56.2 (C9)<br>71.5 (C4)<br>120.5 (C11)<br>132.9 (C7)

Table S5: Product ratios table of SdS_{WT} and variant G305E upon incubation with (2*E*,6*E*)-FDP (**1**) at different pH (4.0-10.0), **2:** selina-4(15),7(11)-diene, **3:** germacrene B, **4:** β-farnesene, **5:** germacrene D, **6:** δ-selinene, **7:** α-elemene, **8:** uncharacterized sesquiterpene, **9:** selina-3,7(11) diene, **10:** selin-7(11)-en-4-ol.

**Effect of pH on selinadiene synthase (SdS_{WT}) catalysis**									
**pH**	**2**	**3**	**4**	**5**	**6**	**7**	**8**	**9**	**10**
**4.0**	--	--	--	--	--	--	--	--	--
**5.0**	100.0	--	--	--	--	--	--	--	--
**6.0**	100.0	--	--	--	--	--	--	--	--
**7.0**	95.1	4.9	--	--	--	--	--	--	--
**8.0**	83.7	16.3	--	--	--	--	--	--	--
**9.0**	58.4	41.6	--	--	--	--	--	--	--
**10.0**	14.9	85.1	--	--	--	--	--	--	--
**Effect of pH on selinadiene synthase (SdS) variant G305E catalysis**									
**4.0**	--	--	--	--	--	--	--	--	--
**5.0**	45.5	--	--	--	2.7	--	5.9	3.6	42.4
**6.0**	43.3	--	--	--	2.2	--	3.2	3.5	47.8
**7.0**	57.1	4.4	--	--	4.2	--	4.2	5.5	24.7
**8.0**	52.9	12.9	--	--	4.4	--	3.8	6.3	19.8
**9.0**	50.4	26.7	--	--	2.8	--	1.9	5.5	12.7
**10.0**	13.6	82.4	--	--	--	--	--	1.7	2.3

11. Analysis of MD simulations

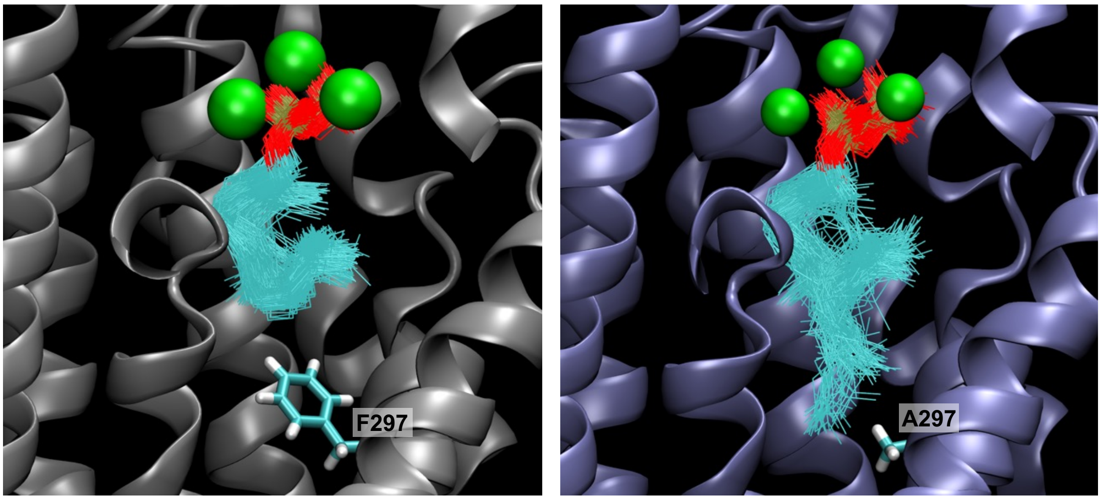

Figure S1. Comparison of FDP conformations in SDS_{WT} (left) and SDS F297A (right). 250 FDP conformations are shown (excluding hydrogens) from the final 20 ns of each independent MD simulation (0.8 ns intervals). This reveals a significant proportion of extended conformations due to the F297A mutation.

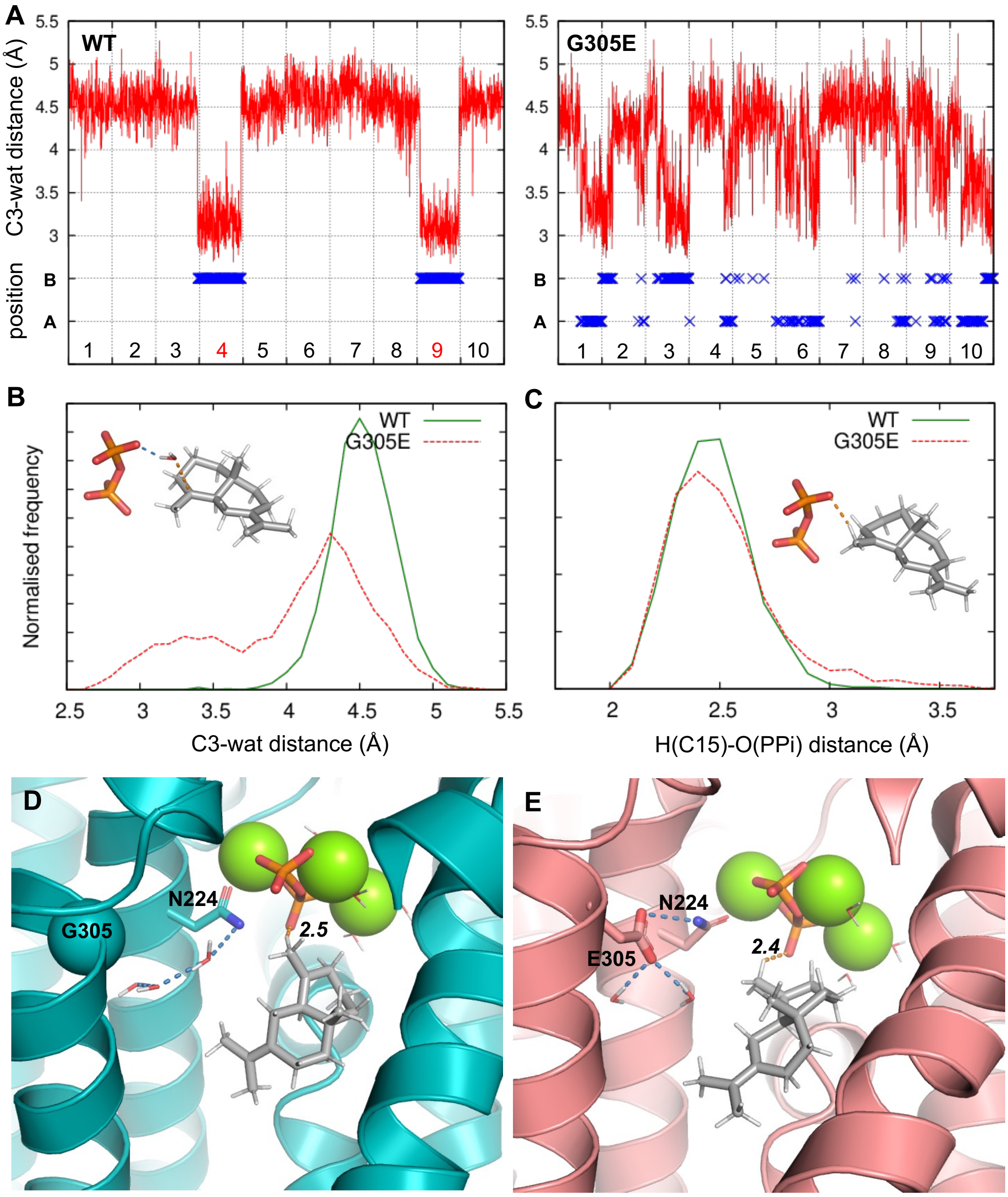

Figure S2. Analysis of interactions of carbocation B and water/PPi in the QM/MM MD (DFTB3/CHARMM36) simulations with SDS_{WT} and SDS G305E. **A)** Distance of the closest water molecule oxygen (O) to carbon C3 (FDP numbering), which gets hydroxylated upon formation of selin-7(11)-en-4-ol, from 10 independent simulations. The position relative to C3 is indicated with blue crosses when the C3-O distance is < 3.4 Å. Position A is pre-*S*, position B is pre-*R*; see main manuscript Figure 3. In SDS_{WT} runs 4 and 9, a water is 'stuck' in position B permanently, which is likely an artifact. **B)** Histogram of C3-O distances from all simulations (excl. SDS_{WT} runs 4 and 9). **C)** Histogram of the shortest PPi oxygen to H_{C15} distances from all simulations, related to the proton abstraction required to form selinadiene. **D)** Representative conformation in SdS_{WT}, together with the likely proton abstraction by PPi (orange dashed line) leading to selinadiene formation. The water cluster that is part of the active-site contour is shown. **E)** Representative structure carbocation B in SdS G305E when a water molecule is > 4 Å away from C3. Distance for proton abstraction by PPi indicated (orange dashed line) and water molecules hydrogen bonding with E305 are shown.

12. Total ion chromatograms (TICs)

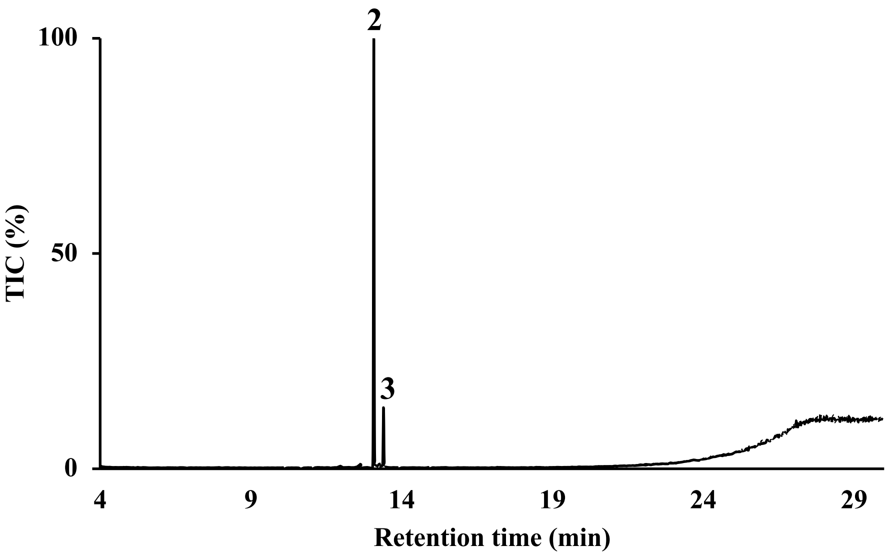

Figure S3. Total ion chromatogram (TIC) of pentane extractable product arising from the incubation of (2*E*,6*E*)-FDP (**1**) with SdS_{WT}, producing selina-4(15),7(11)-diene (**2**) as a major product along with small percentage of germacrene B (**3**).

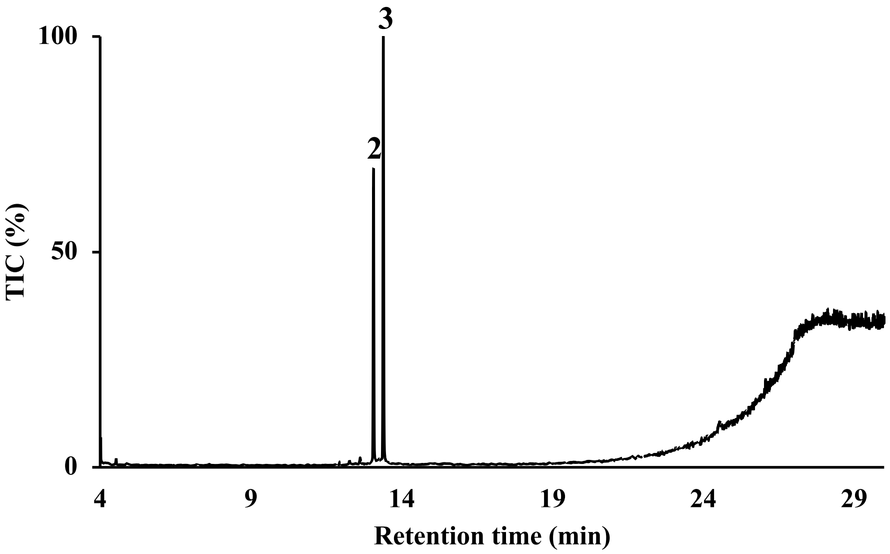

Figure S4. Total ion chromatogram (TIC) of pentane extractable product arising from the incubation of (2*E*,6*E*)-FDP (**1**) with SdS D181V, germacrene B (**3**) as a major product along with selina-4(15),7(11)-diene (**2**).

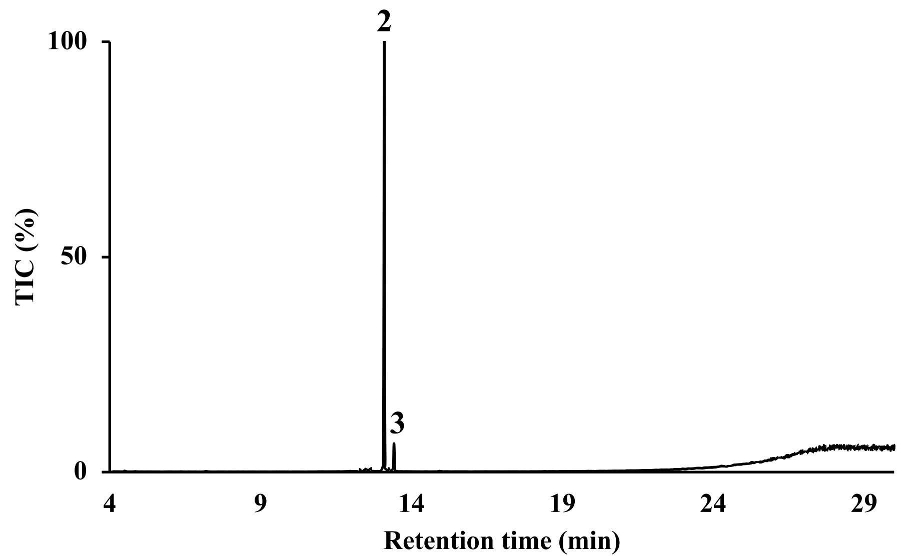

Figure S5. Total ion chromatogram (TIC) of pentane extractable product arising from the incubation of (2*E*,6*E*)-FDP (**1**) with SdS A183G, producing selina-4(15),7(11)-diene (**2**) as a major product along with small percentage of germacrene B (**3**).

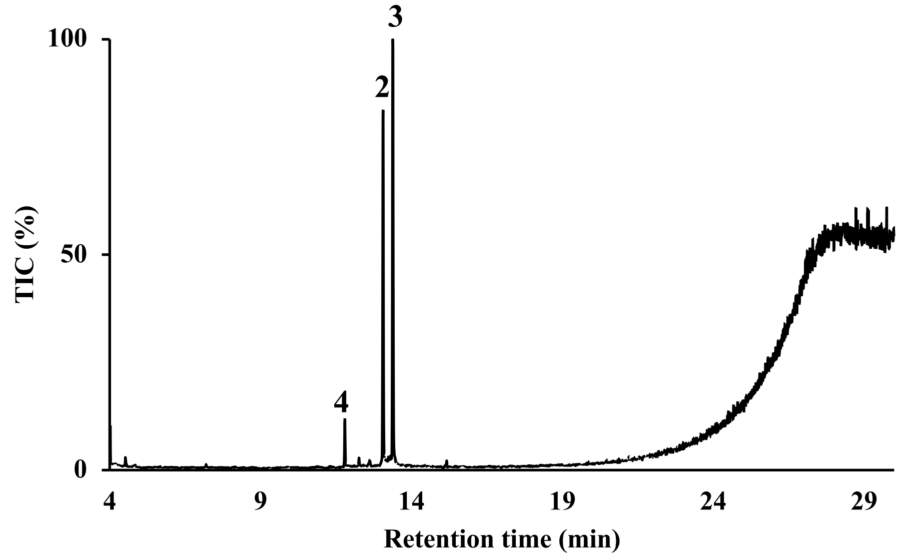

Figure S6. Total ion chromatogram (TIC) of pentane extractable product arising from the incubation of (2*E*,6*E*)-FDP (**1**) with SdS F297A, producing germacrene B (**3**) as a major product with reduced level of selina-4(15),7(11)-diene (**2**) and small percentage of β-farnesene (**4**).

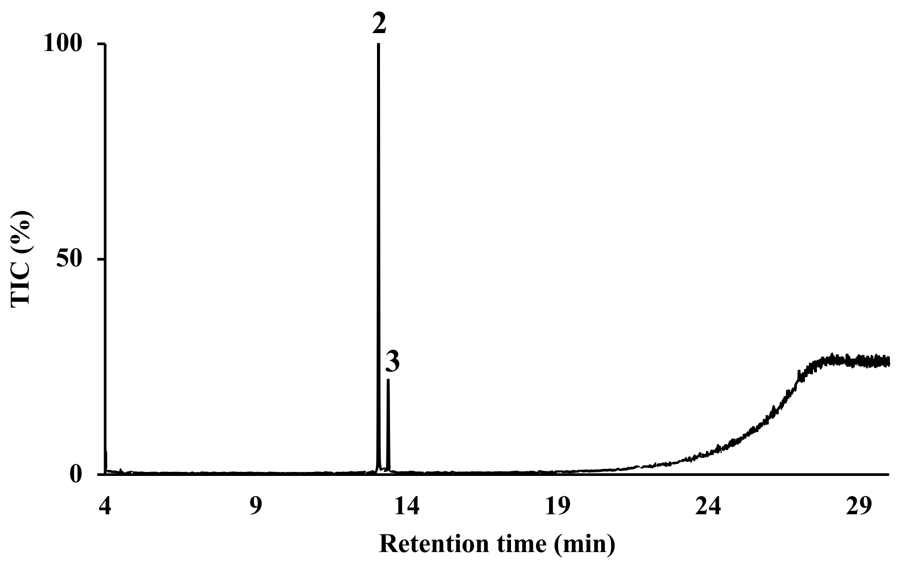

Figure S7. Total ion chromatogram (TIC) of pentane extractable product arising from the incubation of (2*E*,6*E*)-FDP (**1**) with SdS F297W, producing selina-4(15),7(11)-diene (**2**) as a major product along with small percentage of germacrene B (**3**).

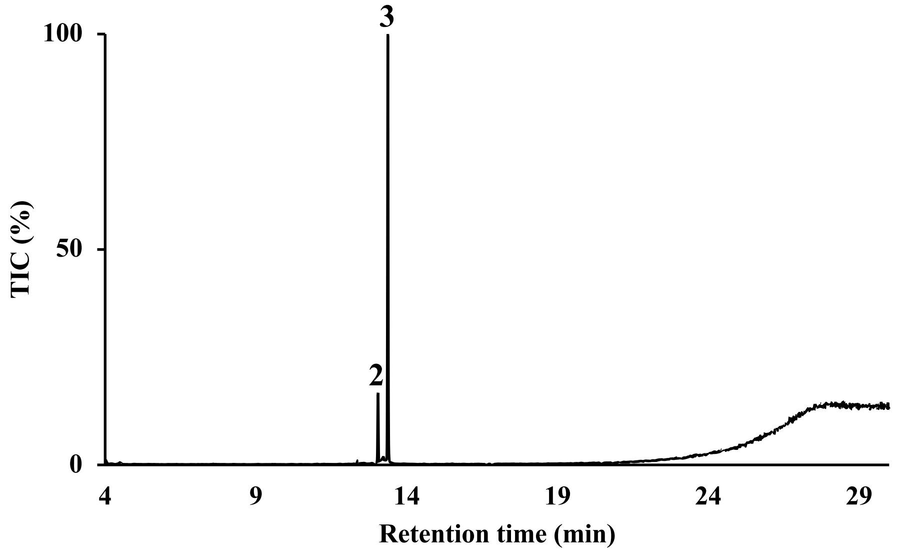

Figure S8. Total ion chromatogram (TIC) of pentane extractable product arising from the incubation of (2*E*,6*E*)-FDP (**1**) with SdS A301Y, producing germacrene B (**3**) as a major product along with small percentage of selina-4(15),7(11)-diene (**2**).

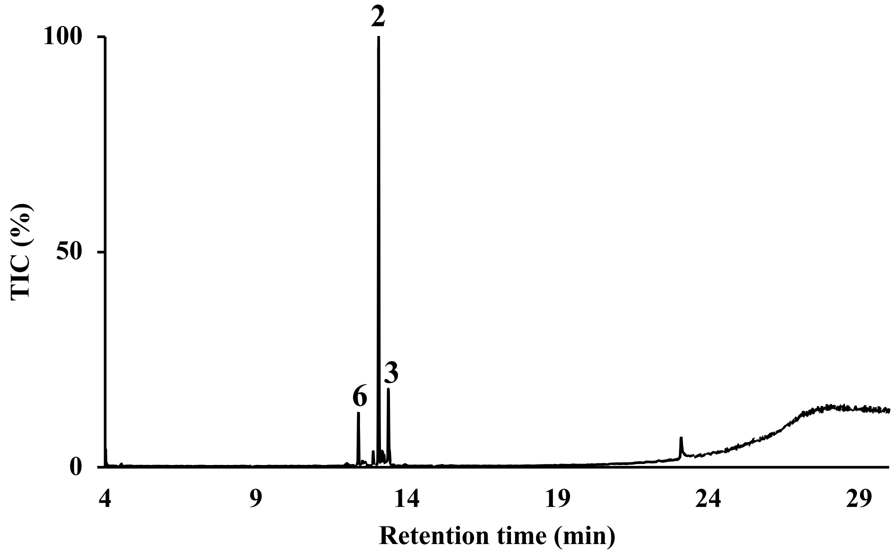

Figure S9. Total ion chromatogram (TIC) of pentane extractable product arising from the incubation of (2*E*,6*E*)-FDP (**1**) with SdS A301D, producing selina-4(15),7(11)-diene (**2**) as a major product along with small percentage of germacrene B (**3**) and δ-selinene (**6**).

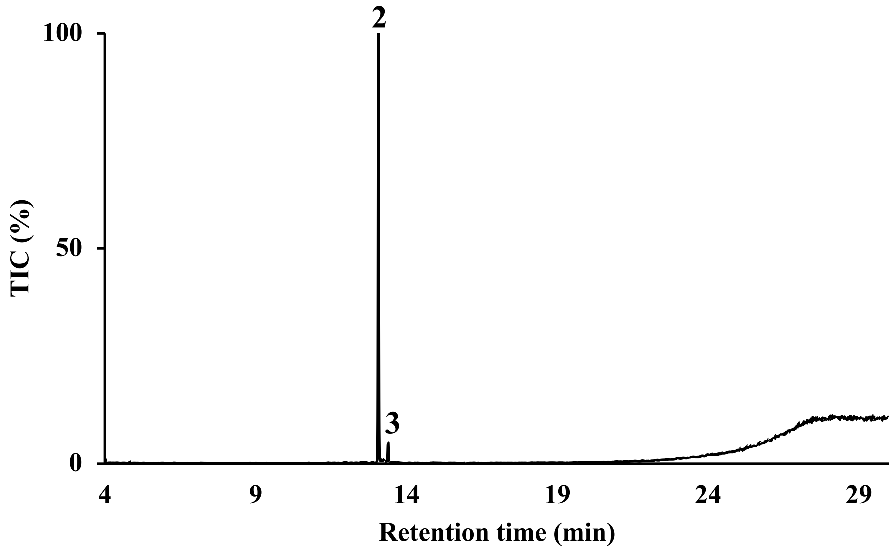

Figure S10. Total ion chromatogram (TIC) of pentane extractable product arising from the incubation of (2*E*,6*E*)-FDP (**1**) with SdS A301S, producing selina-4(15),7(11)-diene (**2**) as a major product along with small percentage of germacrene B (**3**).

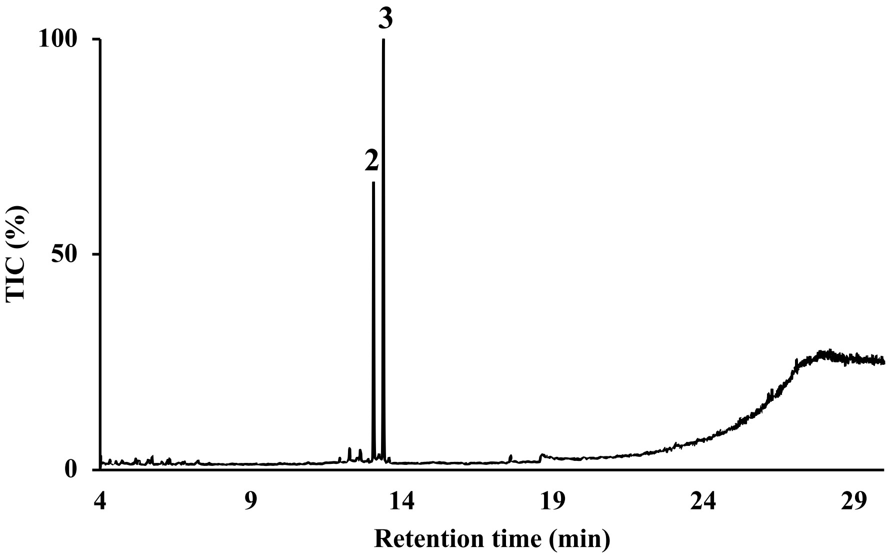

Figure S11. Total ion chromatogram (TIC) of pentane extractable product arising from the incubation of (2*E*,6*E*)-FDP (**1**) with SdS W304S, producing germacrene B (**3**) as a major product along with reduced level of selina-4(15),7(11)-diene (**2**).

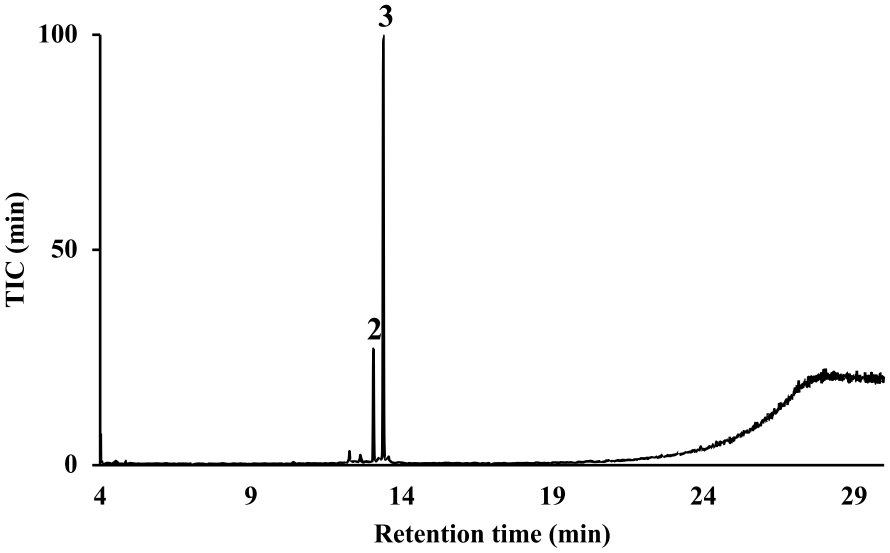

Figure S12. Total ion chromatogram (TIC) of pentane extractable product arising from the incubation of (2*E*,6*E*)-FDP (**1**) with SdS W304E, producing germacrene B (**3**) as a major product along with reduced level of selina-4(15),7(11)-diene (**2**).

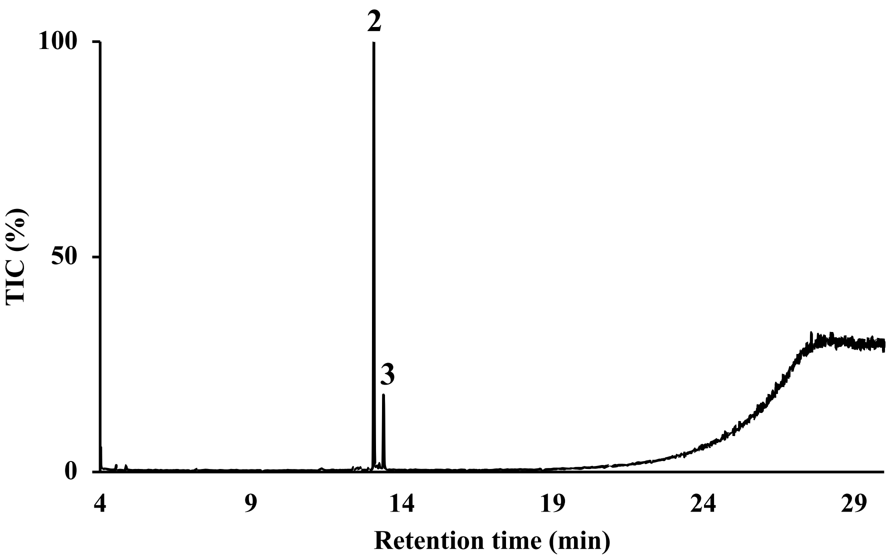

Figure S13. Total ion chromatogram (TIC) of pentane extractable product arising from the incubation of (2*E*,6*E*)-FDP (**1**) with SdS G305H, producing selina-4(15),7(11)-diene (**2**) as a major product along with traces of germacrene B (**3**).

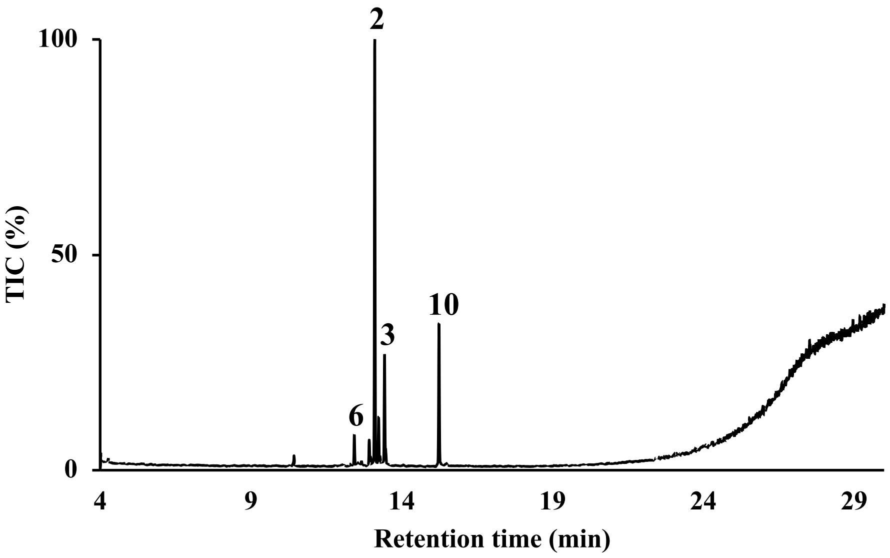

Figure S14. Total ion chromatogram (TIC) of pentane extractable product arising from the incubation of (2*E*,6*E*)-FDP (**1**) with SdS G305E, producing selina-4(15),7(11)-diene (**2**) as a major product along with hydroxylated sesquiterpene selin-7(11)-en-4-ol (**10**) and traces of germacrene B (**2**), δ-selinene (**6**), selina-3,7(11)-diene (**9**).

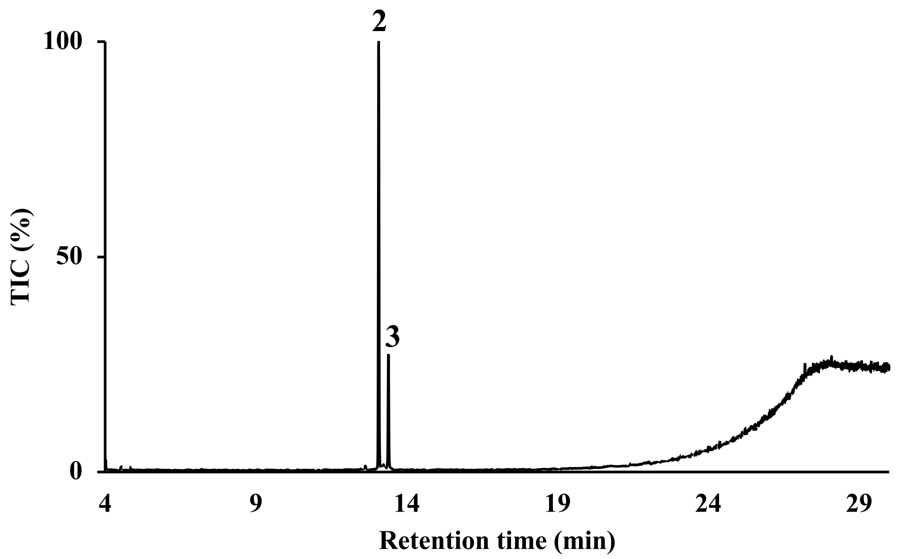

Figure S15. Total ion chromatogram (TIC) of pentane extractable product arising from the incubation of (2*E*,6*E*)-FDP (**1**) with SdS G305D, producing selina-4(15),7(11)-diene (**2**) as a major product along with traces of germacrene B (**3**).

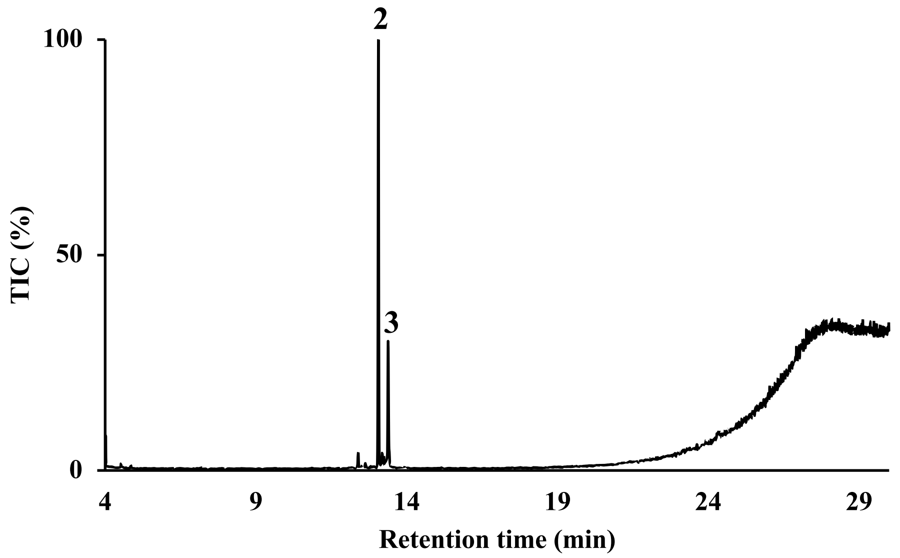

Figure S16. Total ion chromatogram (TIC) of pentane extractable product arising from the incubation of (2*E*,6*E*)-FDP (**1**) with SdS A301N+G305D, producing selina-4(15),7(11)-diene (**2**) as a major product along with small percentage of germacrene B (**3**).

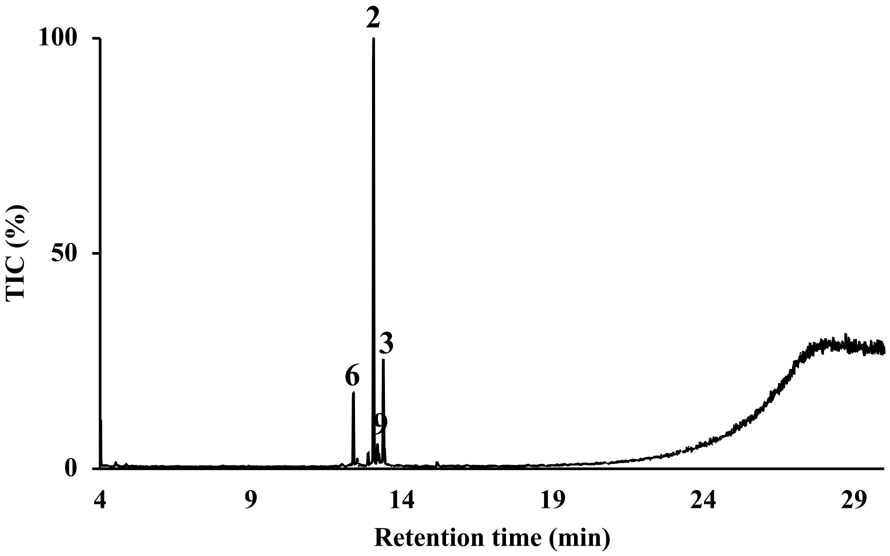

Figure S17. Total ion chromatogram (TIC) of pentane extractable product arising from the incubation of (2*E*,6*E*)-FDP (**1**) with SdS A301N+G305E, producing selina-4(15),7(11)-diene (**2**) as a major product along with germacrene B (**3**), δ-selinene (**6**), and selina-3,7(11)-diene (**9**) in small amounts.

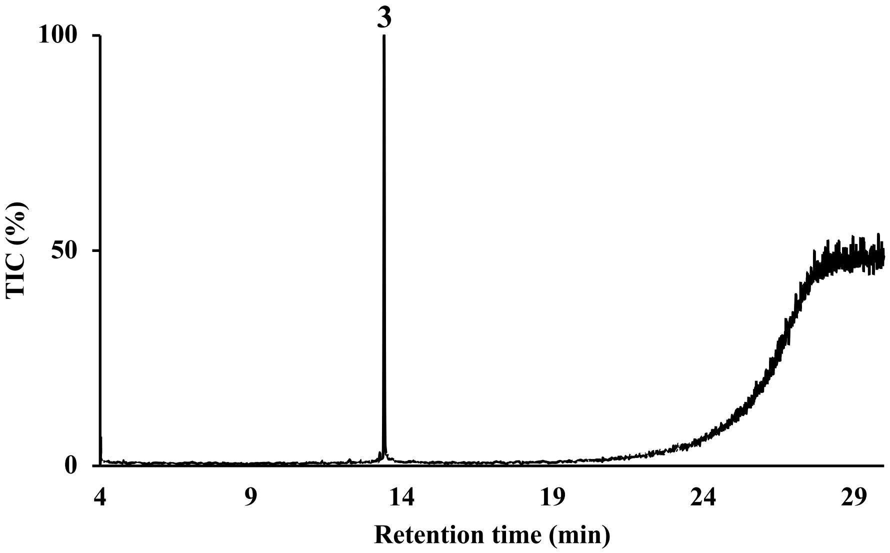

Figure S18. Total ion chromatogram (TIC) of pentane extractable product arising from the incubation of (2*E*,6*E*)-FDP (**1**) with SdS A301Y+G305E, producing germacrene B (**3**) as a sole product.

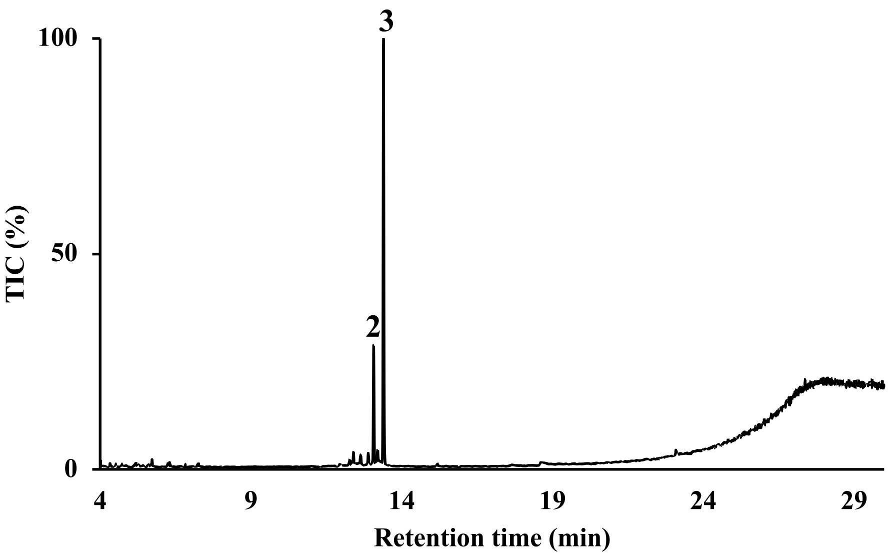

Figure S19. Total ion chromatogram (TIC) of pentane extractable product arising from the incubation of (2*E*,6*E*)-FDP (**1**) with SdS W304S+G305E, producing germacrene B (**3**) as a major product along with reduced level of selina-4(15),7(11)-diene (**2**).

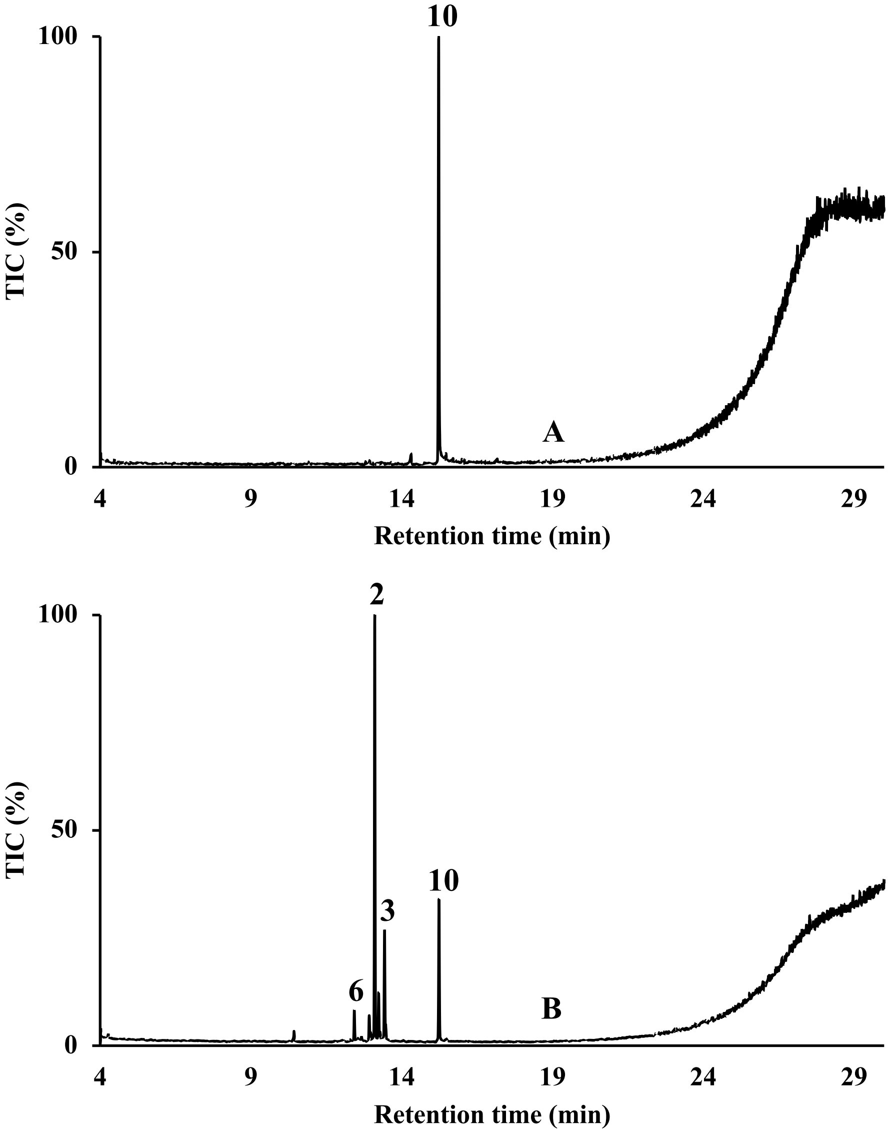

Figure S20. Purification of **10** from large scale assay mixture of SdS G305E. Total ion chromatograms (TIC) of **A)** purified selin-7(11)-en-4-ol (**10**) by column chromatography. This purified sesquiterpene alcohol was confirmed as selin-7(11)-en-4-ol by NMR spectroscopy, **B**) pentane extractable products arising from the incubation of SdS G305E large scale assay with (2*E*,6*E*)-FDP producing selina-4(15),7(11)-diene **(2)** and selin-7(11)-en-4-ol (sesquiterpene alcohol) (**10**).

pH studies

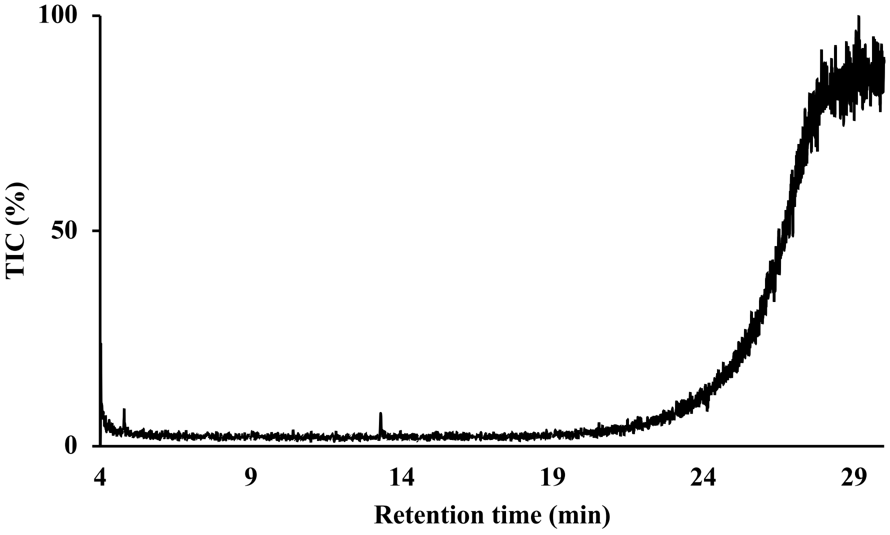

Figure S21. Total ion chromatogram (TIC) of pentane extractable product arising from the incubation of (2*E*,6*E*)-FDP (**1**) with SdS G305E at pH 4.0, resulting in inactive enzyme.

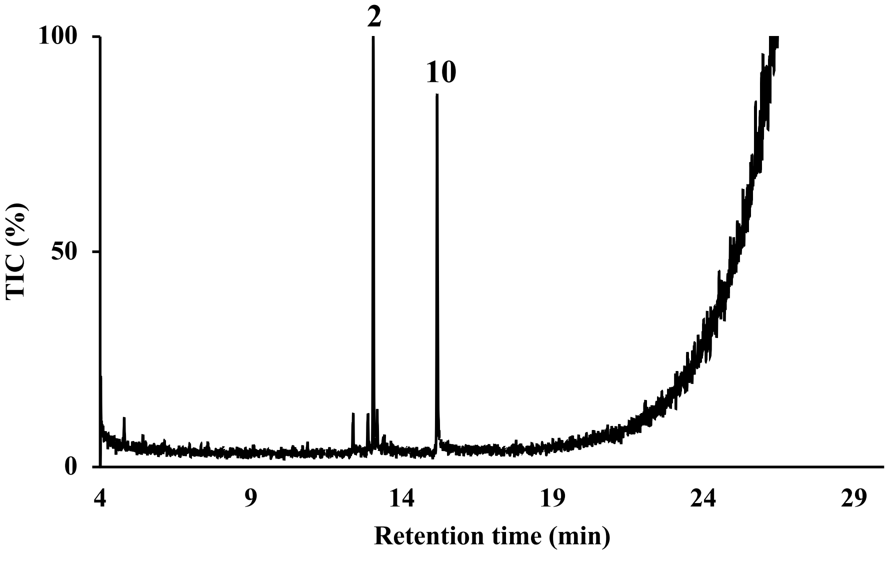

Figure S22. Total ion chromatogram (TIC) of pentane extractable product arising from the incubation of (2*E*,6*E*)-FDP (**1**) with SdS G305E at pH 5.0, producing selina-4(15),7(11)-diene (**2**) as a major product along with hydroxylated sesquiterpene selin-7(11)-en-4-ol (**10**).

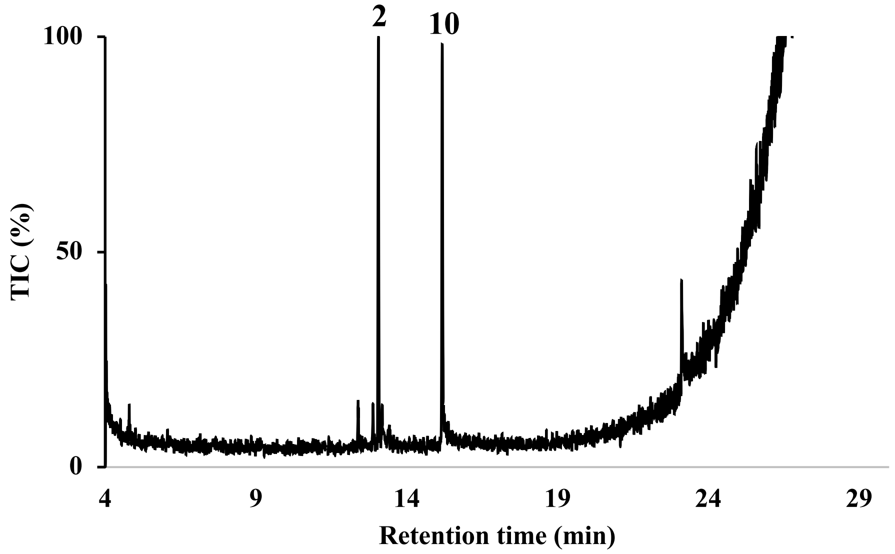

Figure S23. Total ion chromatogram (TIC) of pentane extractable product arising from the incubation of (2*E*,6*E*)-FDP (**1**) with SdS G305E at pH 6.0, producing hydroxylated sesquiterpene selin-7(11)-en-4-ol (**10**) as a major product along with selina-4(15),7(11)-diene (**2**).

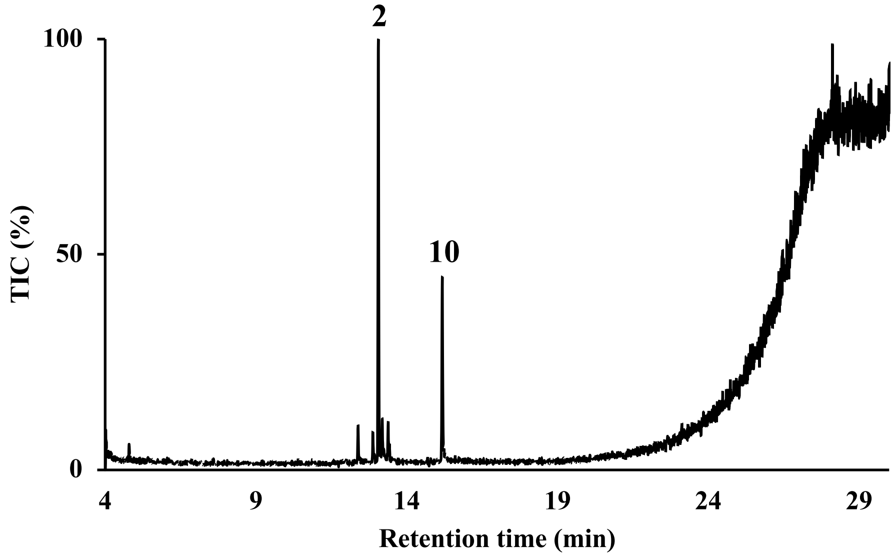

Figure S24. Total ion chromatogram (TIC) of pentane extractable product arising from the incubation of (2*E*,6*E*)-FDP (**1**) with SdS G305E at pH 7.0, producing selina-4(15),7(11)-diene (**2**) as a major product along with hydroxylated sesquiterpene selin-7(11)-en-4-ol (**10**).

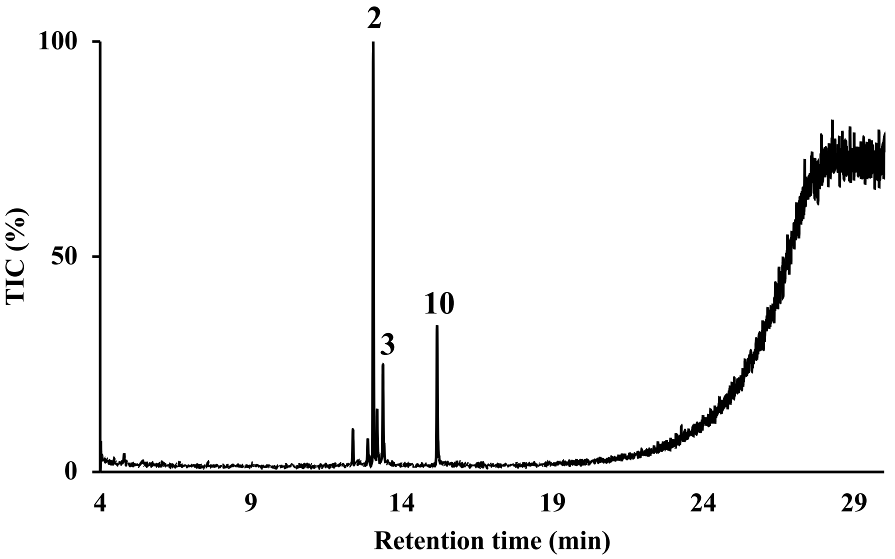

Figure S25. Total ion chromatogram (TIC) of pentane extractable product arising from the incubation of (2*E*,6*E*)-FDP (**1**) with SdS G305E at pH 8.0, producing selina-4(15),7(11)-diene (**2**) as a major product along with hydroxylated sesquiterpene selin-7(11)-en-4-ol (**10**).

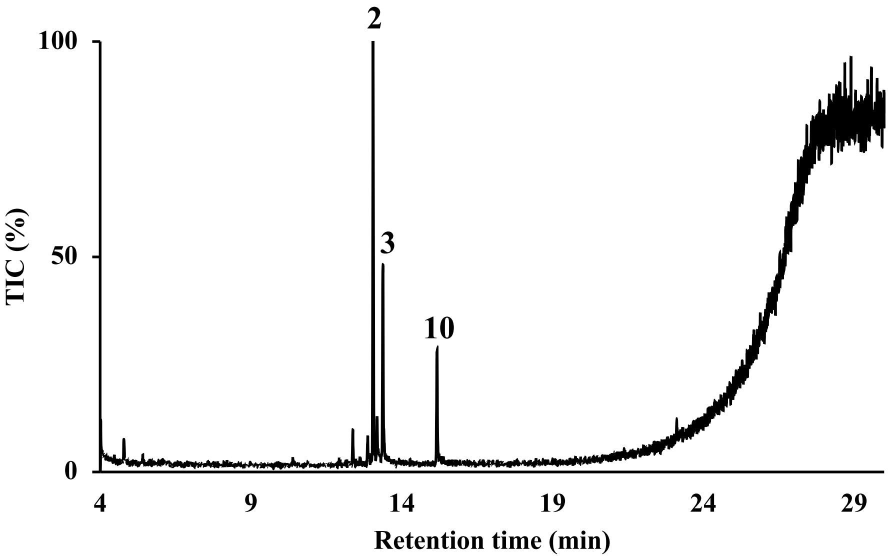

Figure S26. Total ion chromatogram (TIC) of pentane extractable product arising from the incubation of (2*E*,6*E*)-FDP (**1**) with SdS G305E at pH 9.0, producing selina-4(15),7(11)-diene (**2**) as a major product along with hydroxylated sesquiterpene selin-7(11)-en-4-ol (**10**).

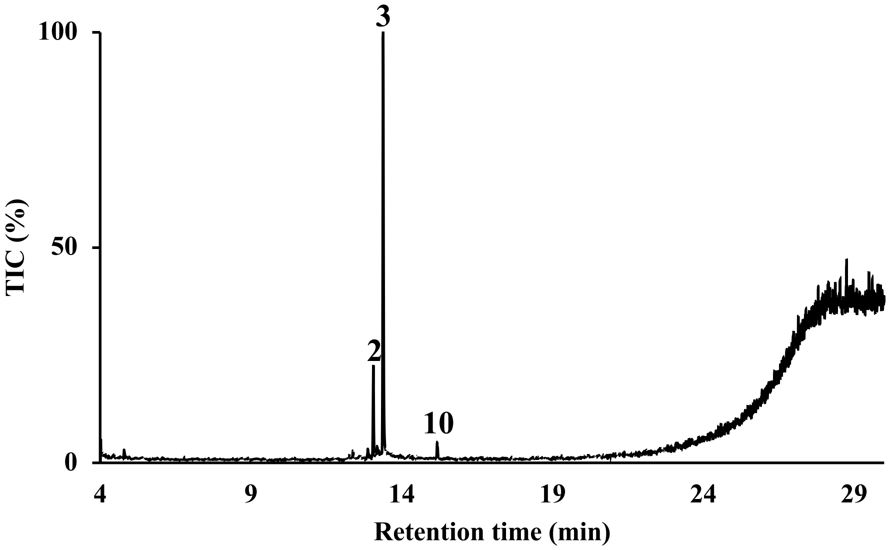

Figure S27. Total ion chromatogram (TIC) of pentane extractable product arising from the incubation of (2*E*,6*E*)-FDP (**1**) with SdS G305E at pH 10.0, producing germacrene B (**3**) as a major product along with traces of selina-4(15),7(11)-diene (**2**) and hydroxylated sesquiterpene selin-7(11)-en-4-ol (**10**).

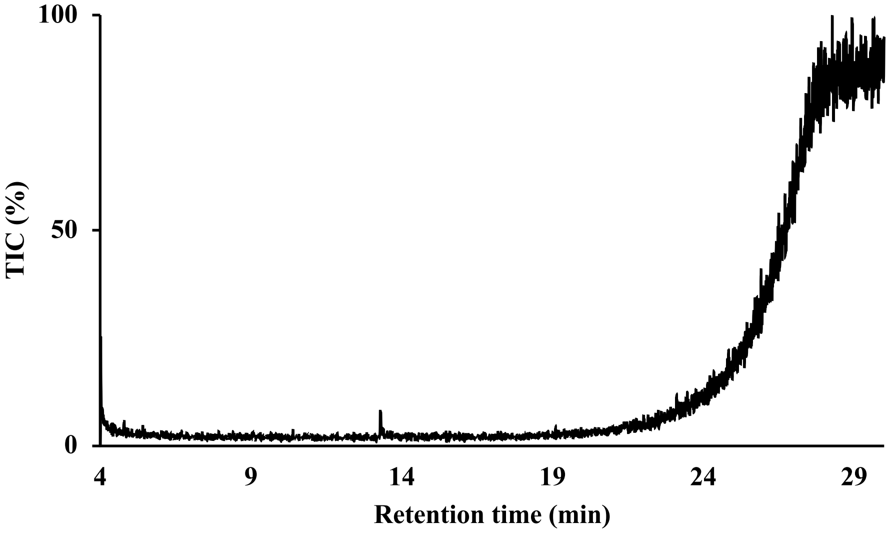

Figure S28. Total ion chromatogram (TIC) of pentane extractable product arising from the incubation of (2*E*,6*E*)-FDP (**1**) with SdS$_{WT}$ at pH 4.0, resulting in inactive enzyme.

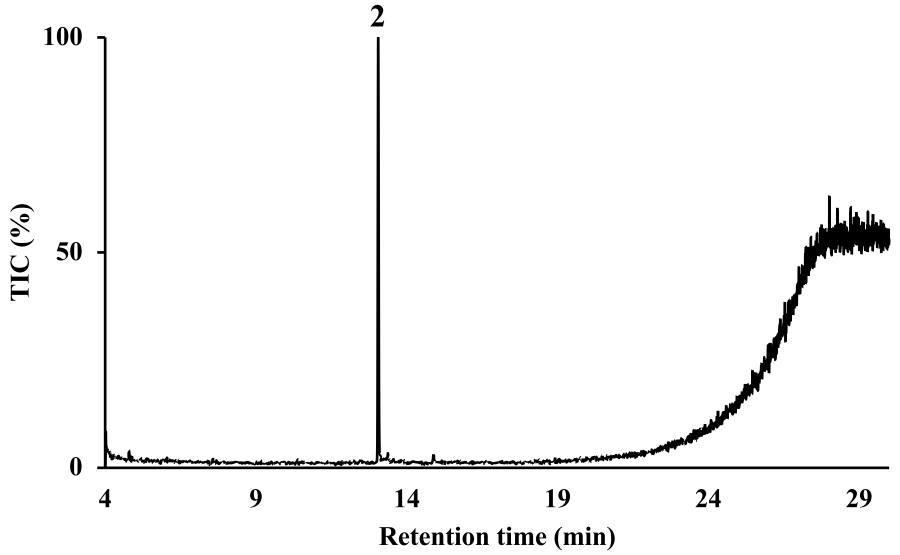

Figure S29. Total ion chromatogram (TIC) of pentane extractable product arising from the incubation of (2*E*,6*E*)-FDP (**1**) with SdS_{WT} pH 5.0, producing selina-4(15),7(11)-diene (**2**) as a sole product.

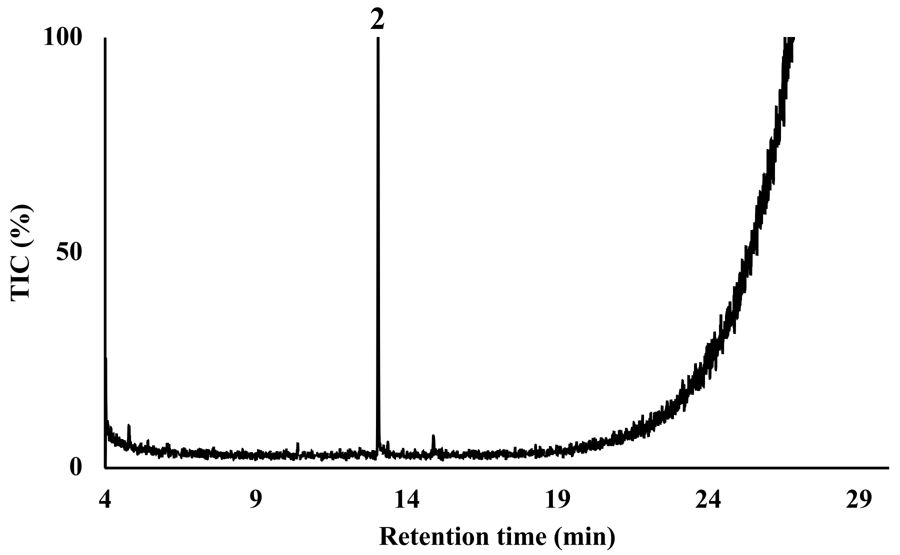

Figure S30. Total ion chromatogram (TIC) of pentane extractable product arising from the incubation of (2*E*,6*E*)-FDP (**1**) with SdS_{WT} pH 6.0, producing selina-4(15),7(11)-diene (**2**) as a sole product.

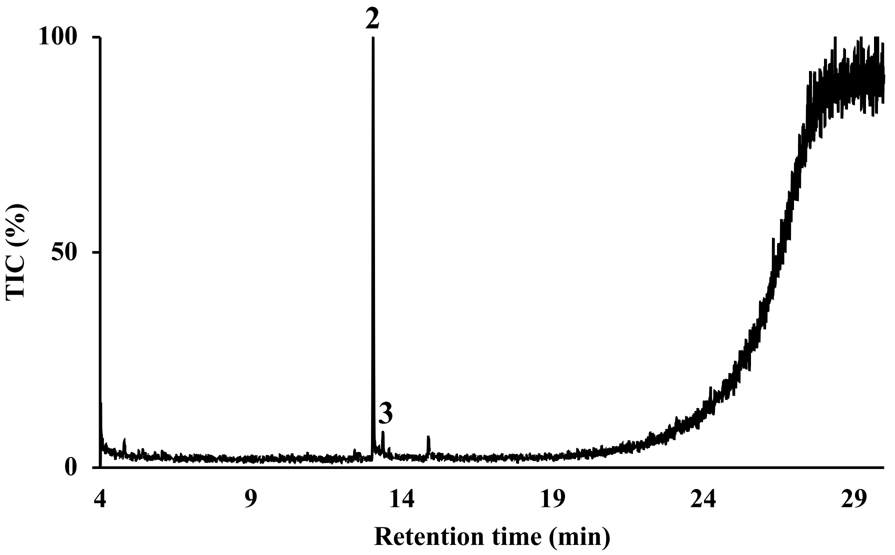

Figure S31. Total ion chromatogram (TIC) of pentane extractable product arising from the incubation of (2*E*,6*E*)-FDP (**1**) with SdS_{WT} pH 7.0, producing selina-4(15),7(11)-diene (**2**) as a main product along with traces of germacrene B (**3**).

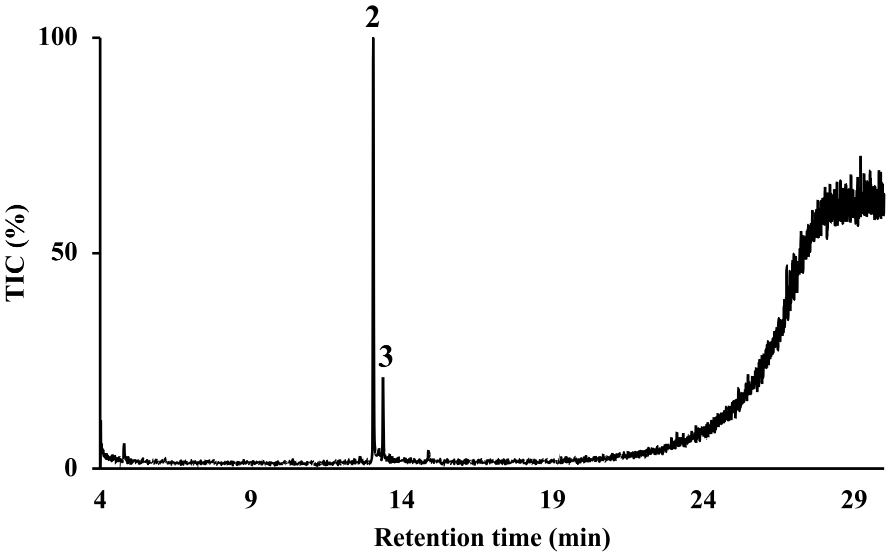

Figure S32. Total ion chromatogram (TIC) of pentane extractable product arising from the incubation of (2*E*,6*E*)-FDP (**1**) with SdS_{WT} pH 8.0, producing selina-4(15),7(11)-diene (**2**) as a main product along with small percentage of germacrene B (**3**).

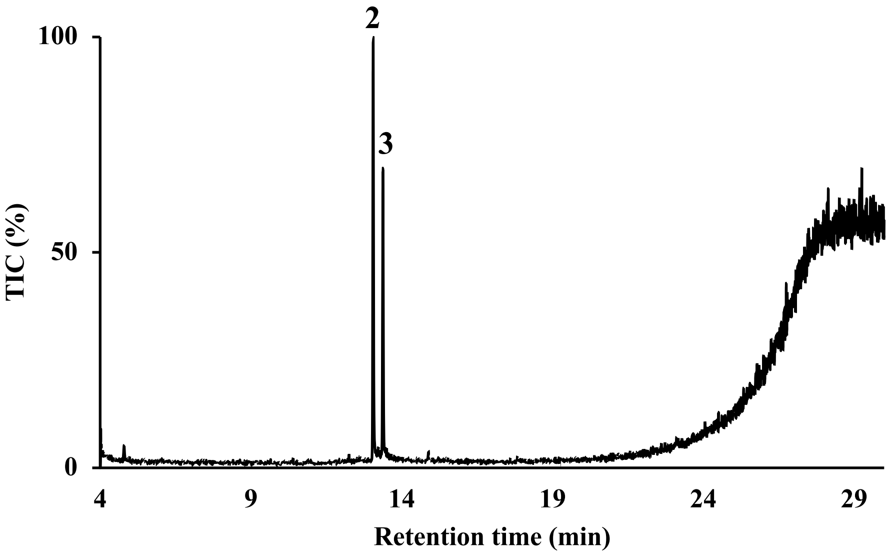

Figure S33. Total ion chromatogram (TIC) of pentane extractable product arising from the incubation of (2*E*,6*E*)-FDP (**1**) with SdS_{WT} pH 9.0, producing selina-4(15),7(11)-diene (**2**) as a main product along with germacrene B (**3**).

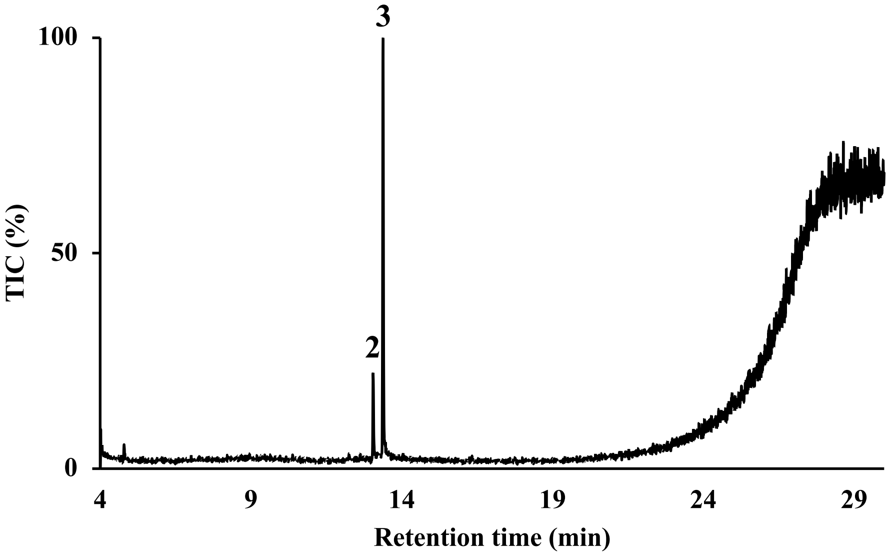

Figure S34. Total ion chromatogram (TIC) of pentane extractable product arising from the incubation of (2*E*,6*E*)-FDP (**1**) with SdS_{WT} pH 10.0, producing germacrene B (**3**) as a main product along with small percentage selina-4(15),7(11)-diene (**2**).

In-vivo production of selin-7(11)-en-4-ol at different pH

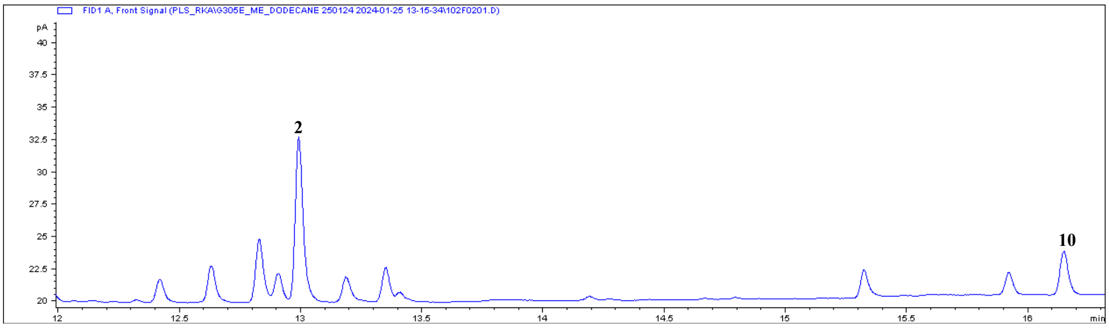

Figure S35. GC analysis of pentane extractable product arising from the *in-vivo* production of selin-7(11)-en-4-ol using metabolic engineering approach in BL21(DE3) cells containing mevalonate pathway genes in pMevT and pMBIS plasmids along with SdS G305E in pET28a at pH 6.0 for 24 hr in 25 mL culture medium, producing selin-7(11)-en-4-ol (**10,** 17%) along with selina-4(15),7(11)-diene as main product (**2**).

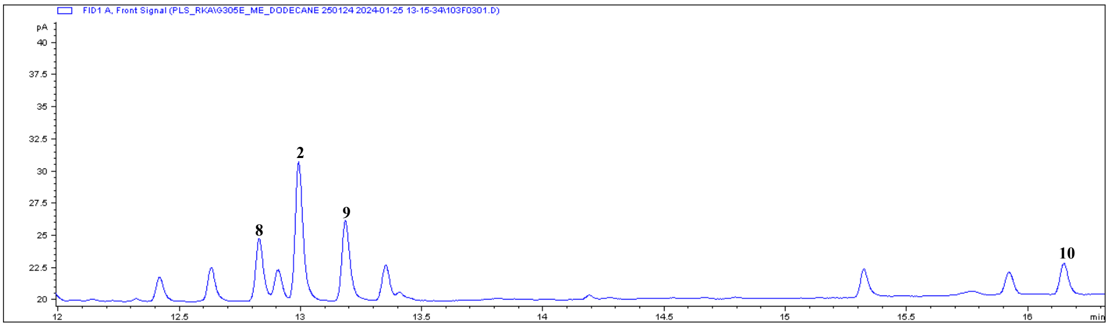

Figure S36. GC analysis of pentane extractable product arising from the *in-vivo* production of selin-7(11)-en-4-ol using metabolic engineering approach in BL21(DE3) cells containing mevalonate pathway genes in pMevT and pMBIS plasmids along with SdS G305E in pET28a at pH 7.0 for 24 hr in 25 mL culture medium, producing selin-7(11)-en-4-ol (**10**) along with selina-4(15),7(11)-diene as a major product (**2**).

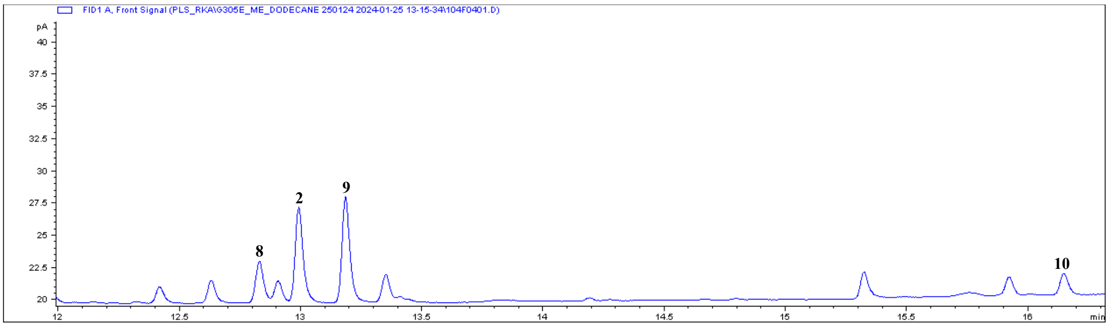

Figure S37. GC analysis of pentane extractable product arising from the *in-vivo* production of selin-7(11)-en-4-ol using metabolic engineering approach in BL21(DE3) cells containing mevalonate pathway genes in pMevT and pMBIS plasmids along with SdS G305E in pET28a at pH 8.0 for 24 hr in 25 mL culture medium, producing selina-3,7(11)-diene (**9**) as a main product along with small percentage of selin-7(11)-en-4-ol (**10**).

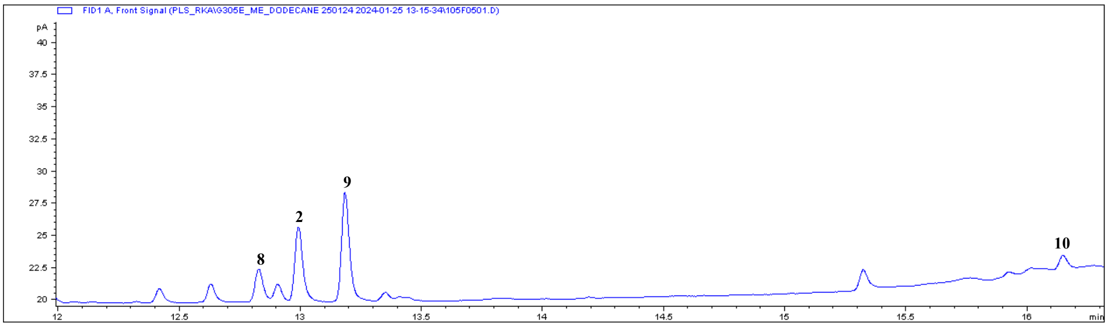

Figure S38. GC analysis of pentane extractable product arising from the *in-vivo* production of selin-7(11)-en-4-ol using metabolic engineering approach in BL21(DE3) cells containing mevalonate pathway genes in pMevT and pMBIS plasmids along with SdS G305E in pET28a at pH 9.0 for 24 hr in 25 mL culture medium, producing selina-3,7(11)-diene (**9**) as a major product along with small percentage selin-7(11)-en-4-ol (**10**).

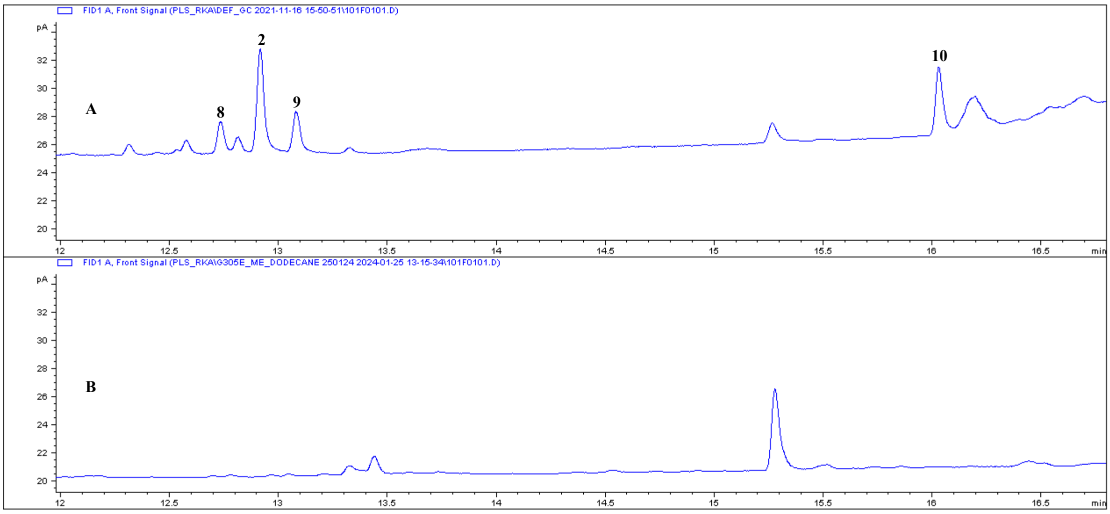

Figure S39. GC analysis of pentane extractable product arising from the *in-vitro* incubation of (2*E*,6*E*)-FDP (**1**) with SdS G305E. **A)** pentane extract of SdS G305E producing selina-4(15),7(11)-diene (**2**) as a main product along with selin-7(11)-en-4-ol (**10**), **B)** pentane blank.

13. GCMS Mass Spectra

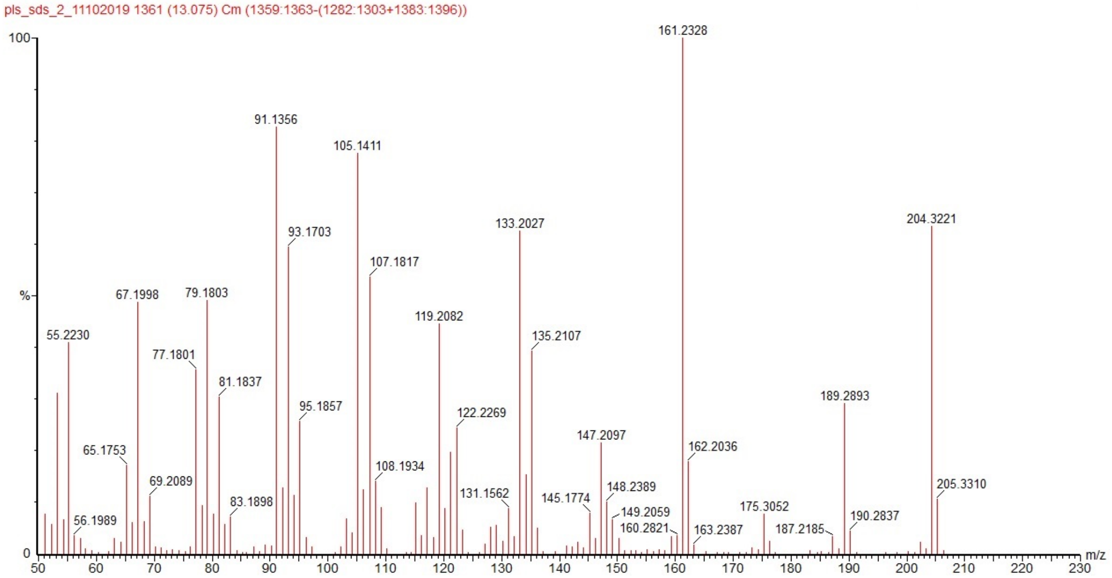

Figure 40: EI Mass spectrum of the compound eluting at 13.06 min in the gas-chromatogram from the incubation of (2*E*,6*E*)-FDP (**1**) with SdS_{WT} (selina-4(15),7(11)-diene, **2**).

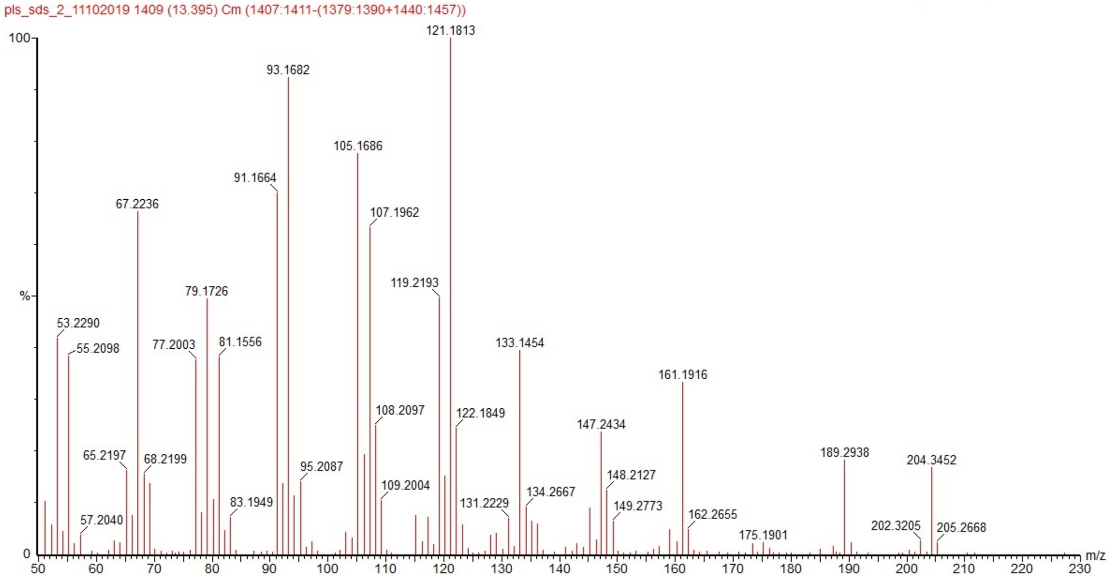

Figure 41: EI Mass spectrum of the compound eluting at 13.38 min in the gas-chromatogram from the incubation of (2*E*,6*E*)-FDP (**1**) with SdS_{WT} (germacrene B, **3**).

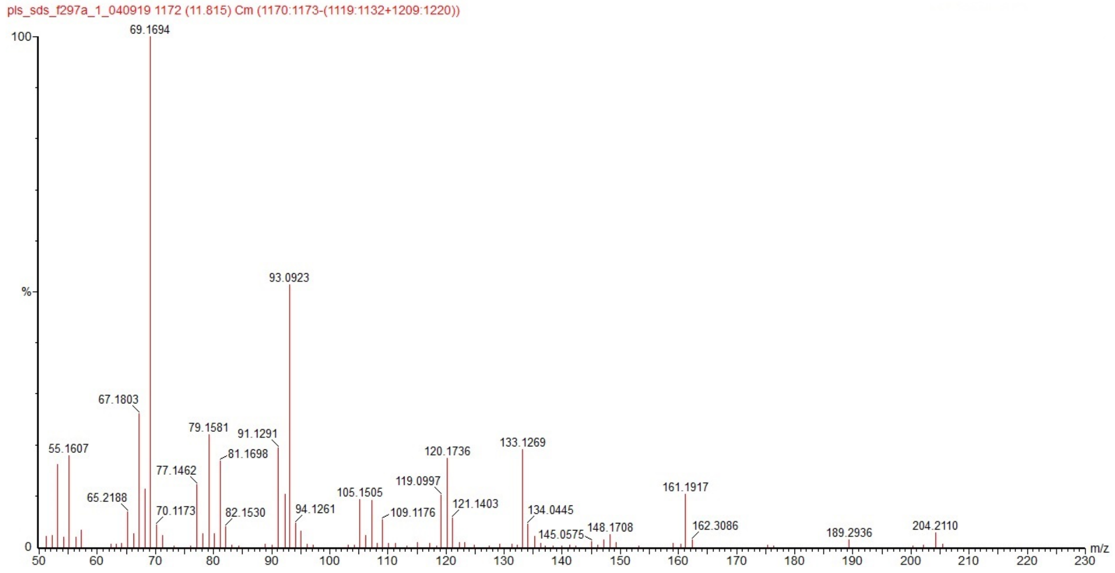

Figure 42: EI Mass spectrum of the compound eluting at 11.80 min in the gas-chromatogram from the incubation of (2*E*,6*E*)-FDP (**1**) with SdS F297A (β-farnesene, **4**).

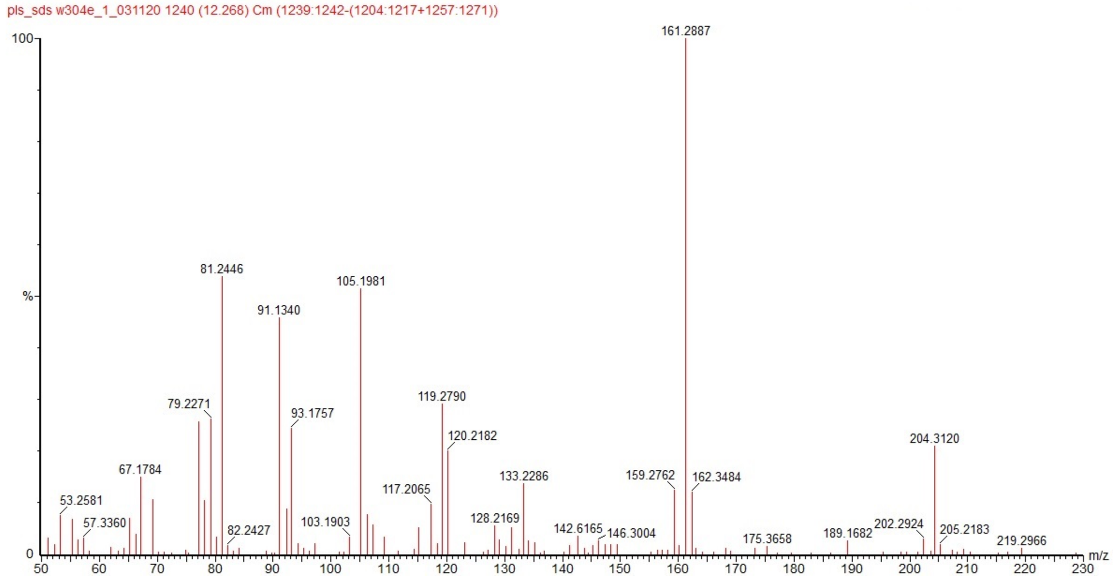

Figure 43: EI Mass spectrum of the compound eluting at 12.28 min in the gas-chromatogram from the incubation of (2*E*,6*E*)-FDP (**1**) with SdS W304S and W304E (germacrene D, **5**).

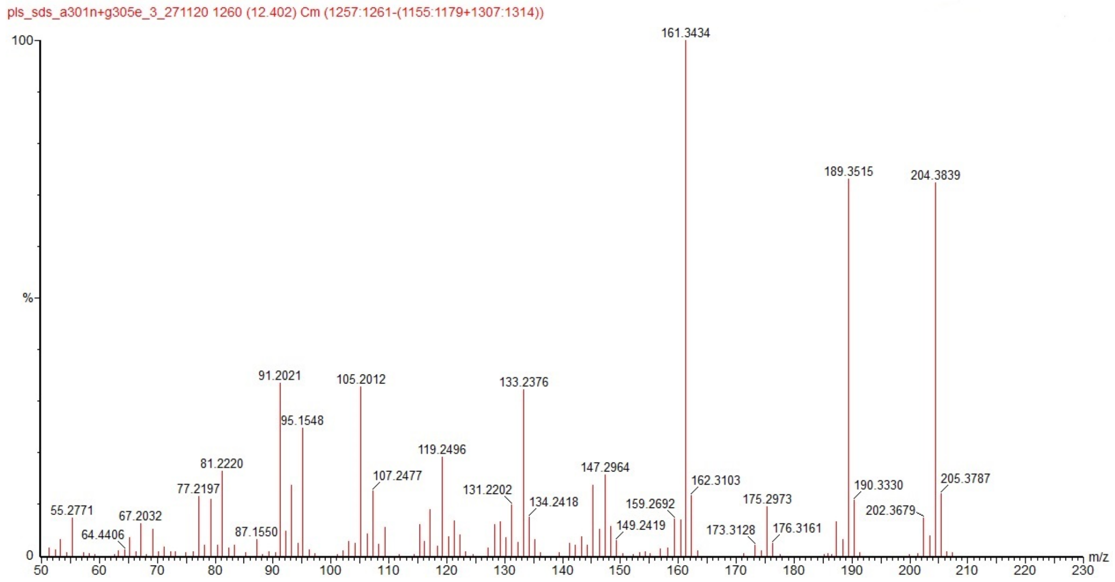

Figure 44: EI Mass spectrum of the compound eluting at 12.37 min in the gas-chromatogram from the incubation of (2*E*,6*E*)-FDP (**1**) with SdS A301D, G305E and A301N+G305E (δ-selinene, **6**).

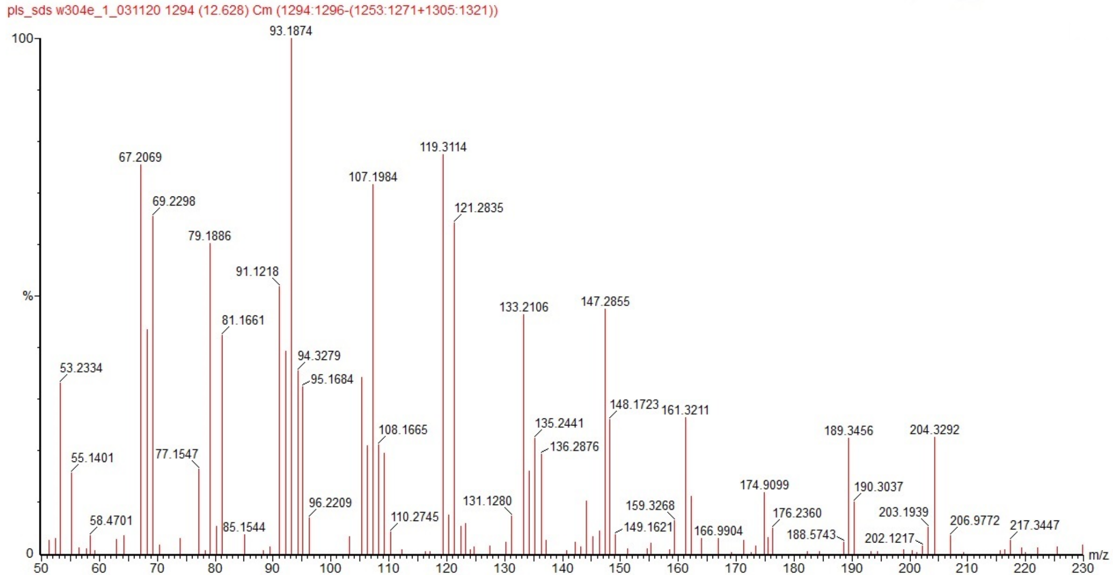

Figure 45: EI Mass spectrum of the compound eluting at 12.63 min in the gas-chromatogram from the incubation of (2*E*,6*E*)-FDP (**1**) with SdS W304S and W304E (α-elemene, **7**).

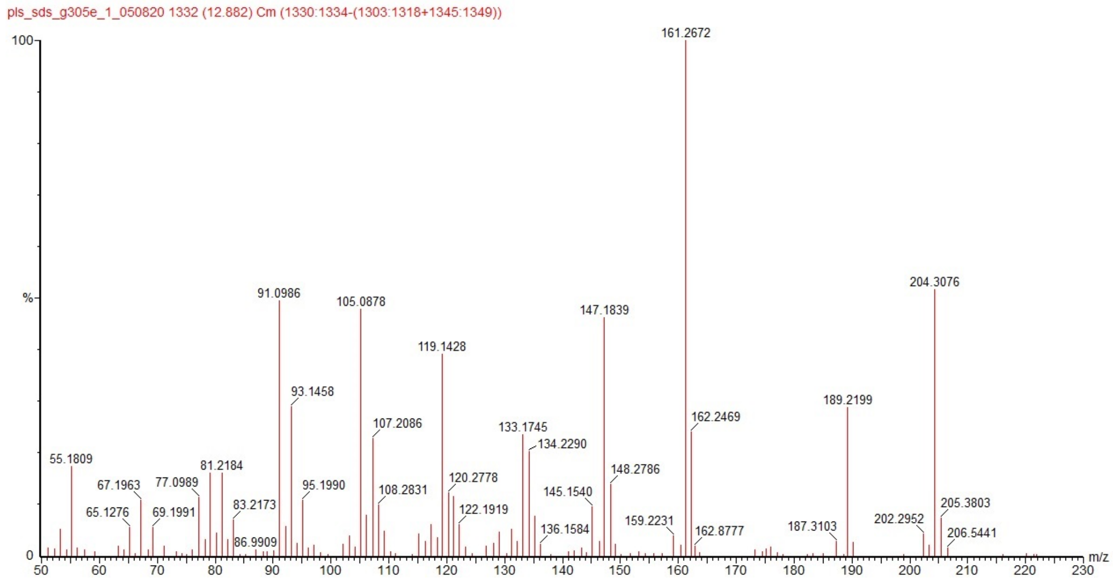

Figure 46: EI Mass spectrum of the compound eluting at 12.88 min in the gas-chromatogram from the incubation of (2*E*,6*E*)-FDP (**1**) with SdS A301D, G305E, A301N+G305E and W304S+G305E (uncharacterized sesquiterpene, **8**).

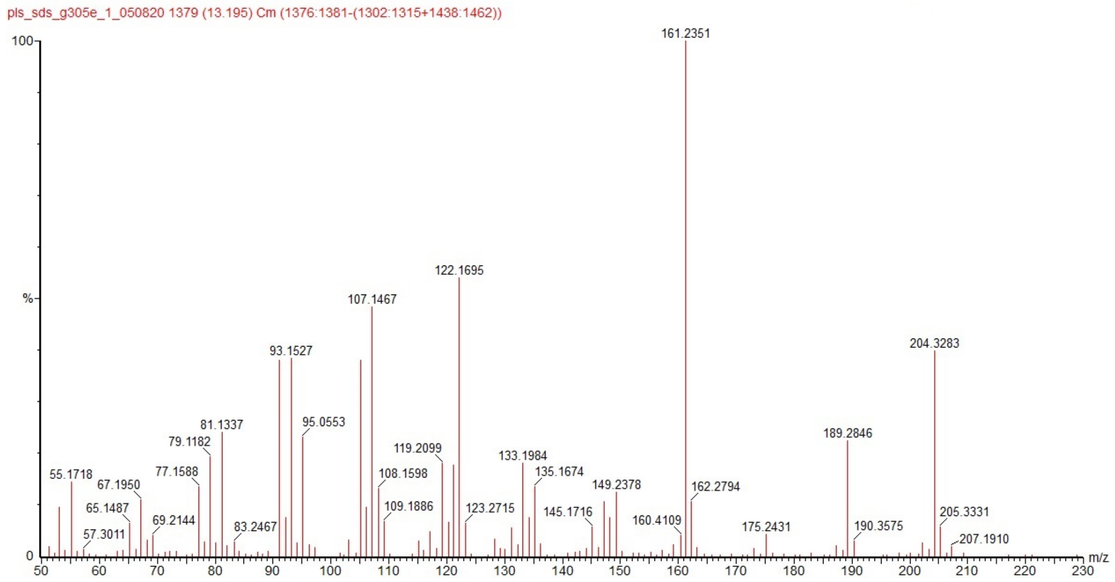

Figure 47: EI Mass spectrum of the compound eluting at 13.20 min in the gas-chromatogram from the incubation of (2*E*,6*E*)-FDP (**1**) with SdS A301D, G305E, A301N+G305E and W304S+G305E (selina-3,7(11)-diene, **9**).

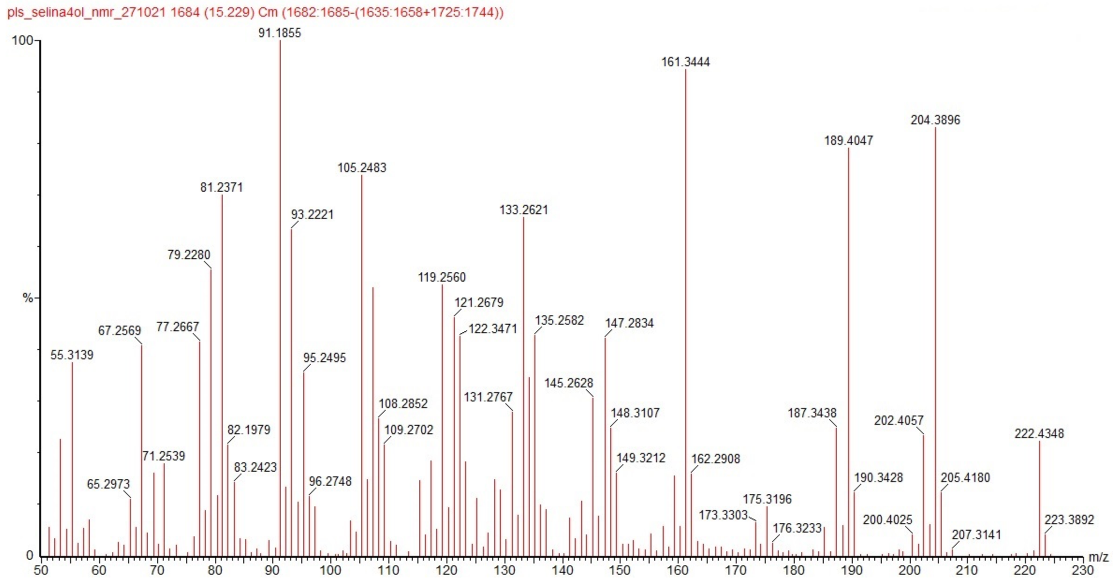

Figure 48: EI Mass spectrum of the compound eluting at 15.17 min in the gas-chromatogram from the incubation of (2*E*,6*E*)-FDP (**1**) with SdS G305E (selin-7(11)-en-4-ol, **10**).

14. Kinetic Data

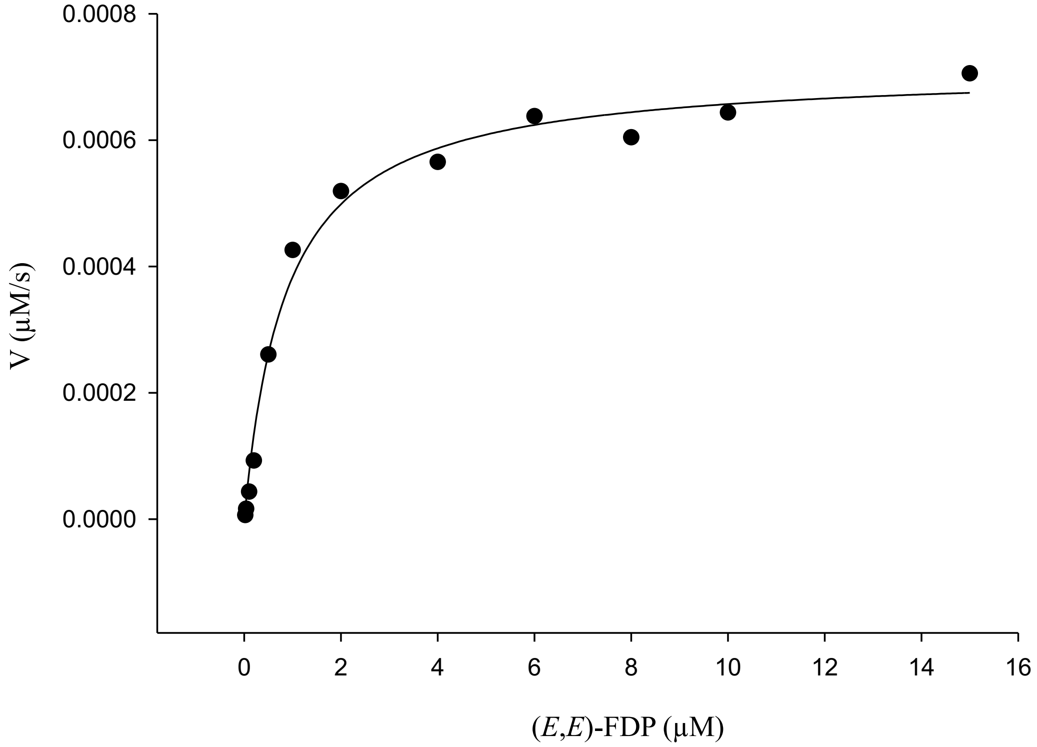

Figure S49: Representative Michaelis-Menten plot for the conversion of [1-^{3}H]-FDP by SdS_{WT}.

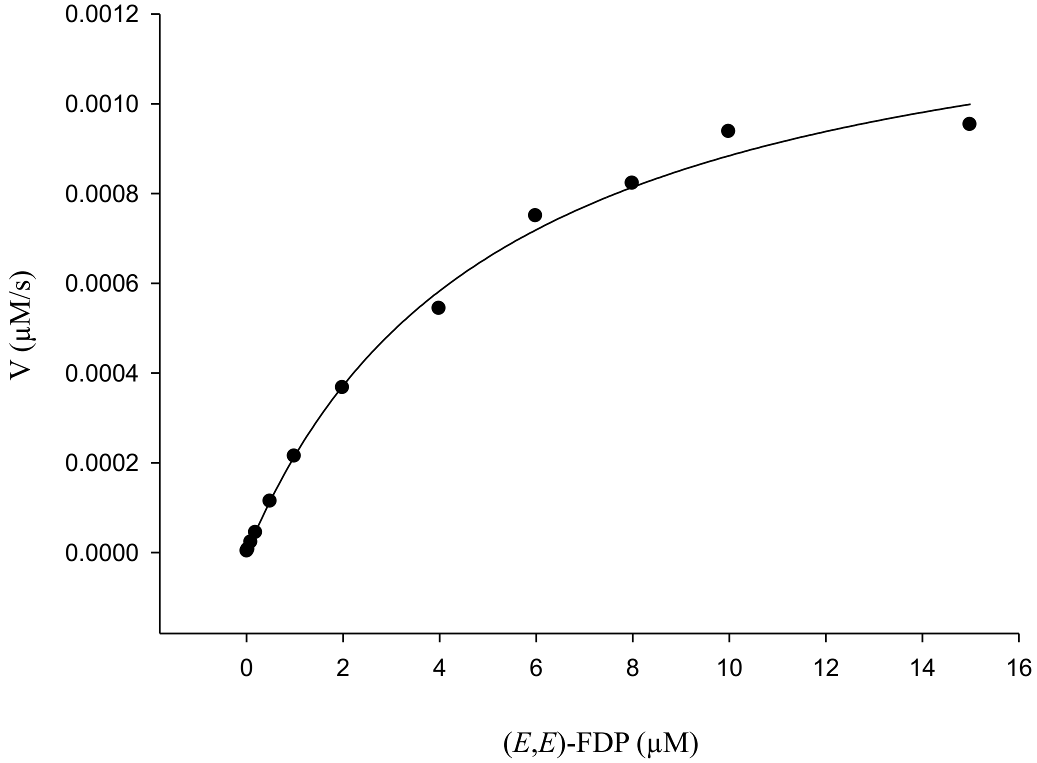

Figure S50: Representative Michaelis-Menten plot for the conversion of [1-^{3}H]-FDP by SdS D181V.

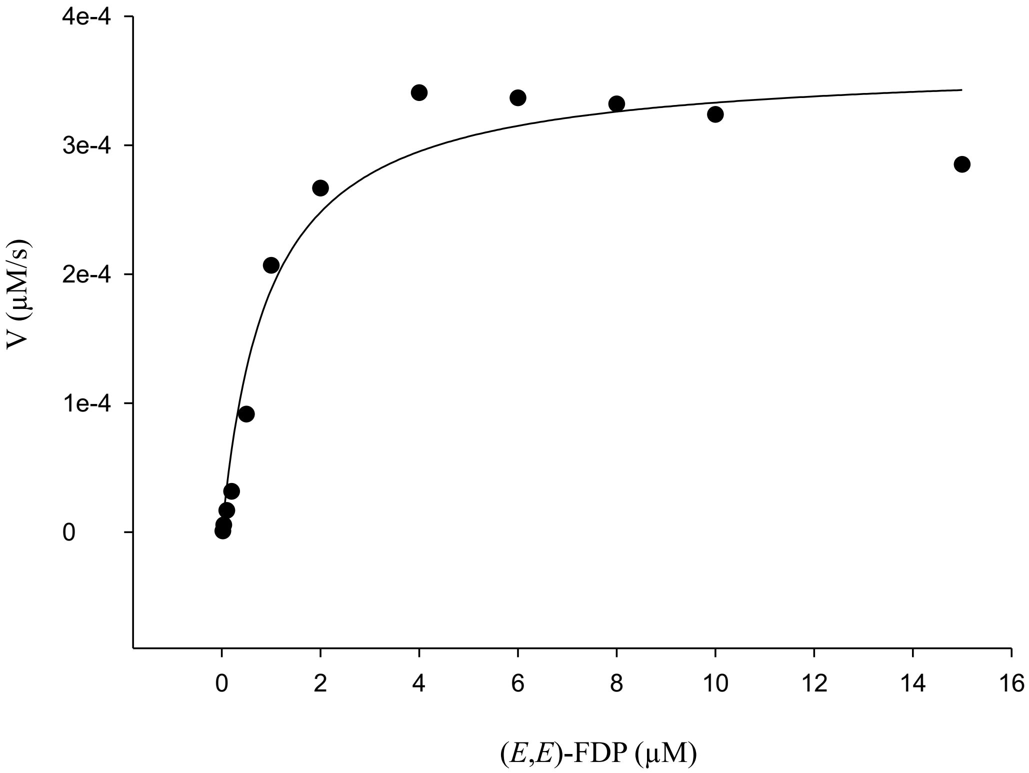

Figure S51: Representative Michaelis-Menten plot for the conversion of [1-^{3}H]-FDP by SdS A183G.

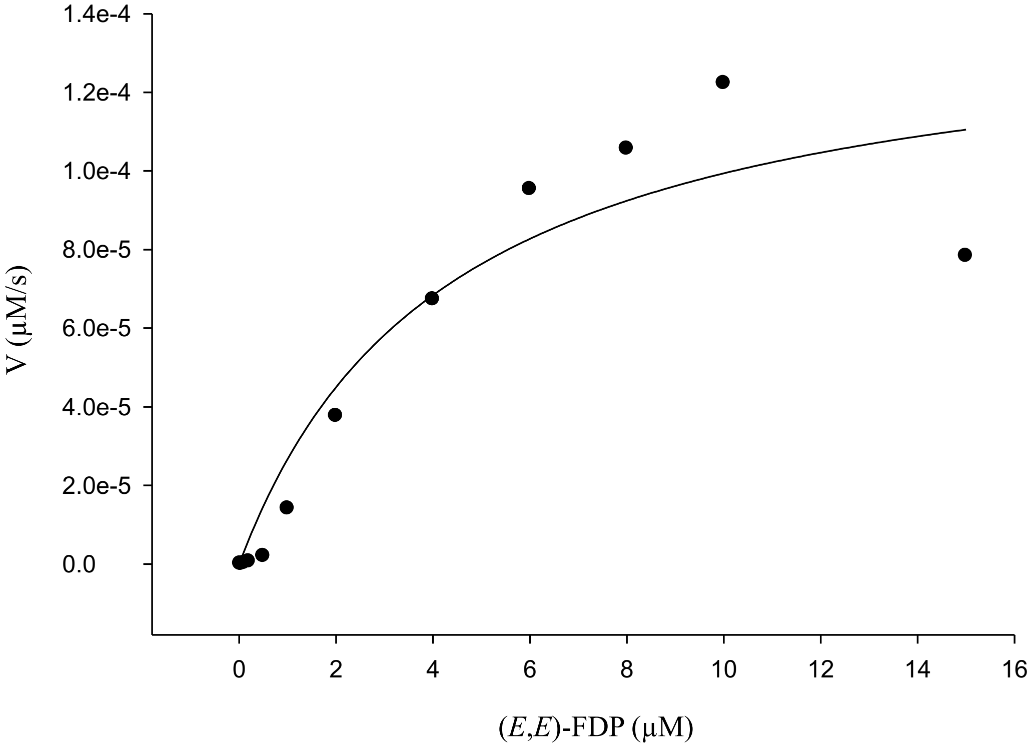

Figure S52: Representative Michaelis-Menten plot for the conversion of [1-^{3}H]-FDP by SdS F297A.

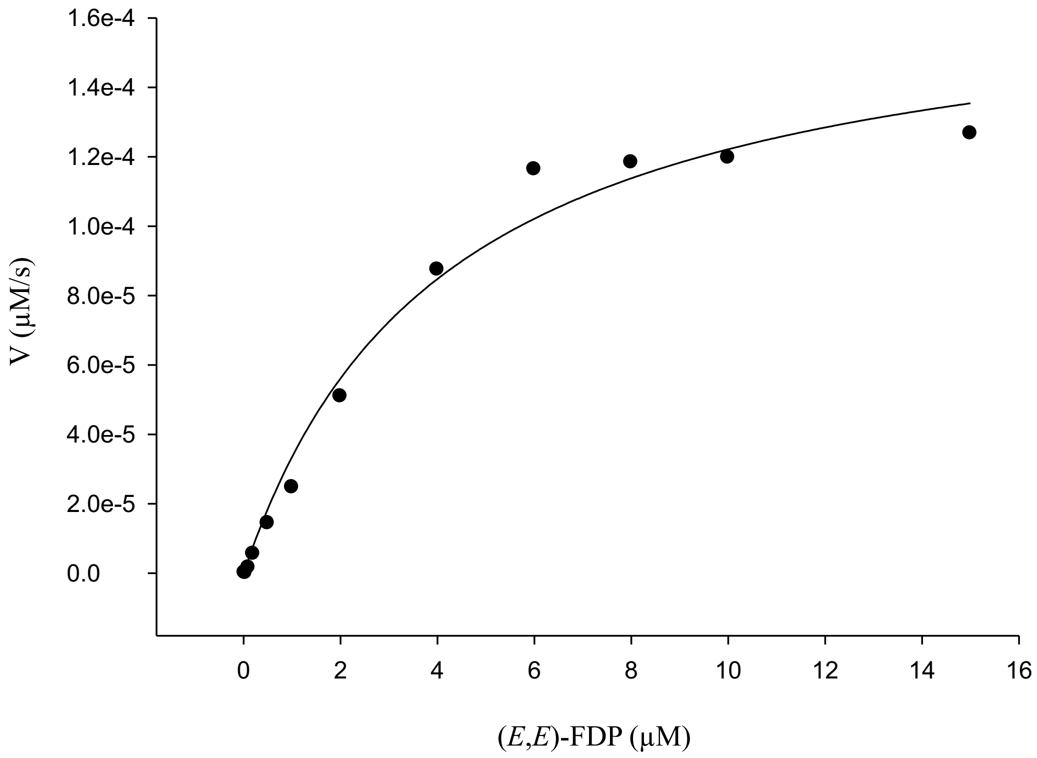

Figure S53: Representative Michaelis-Menten plot for the conversion of [1-^{3}H]-FDP by SdS F297W.

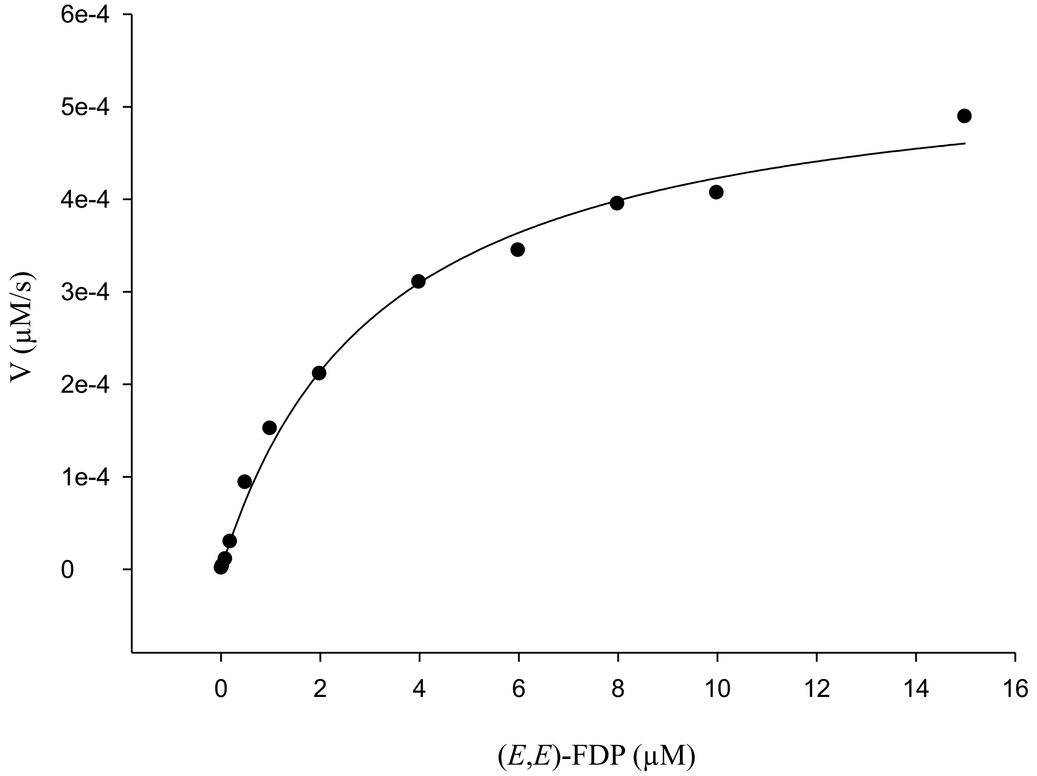

Figure S54: Representative Michaelis-Menten plot for the conversion of [1-^{3}H]-FDP by SdS A301Y.

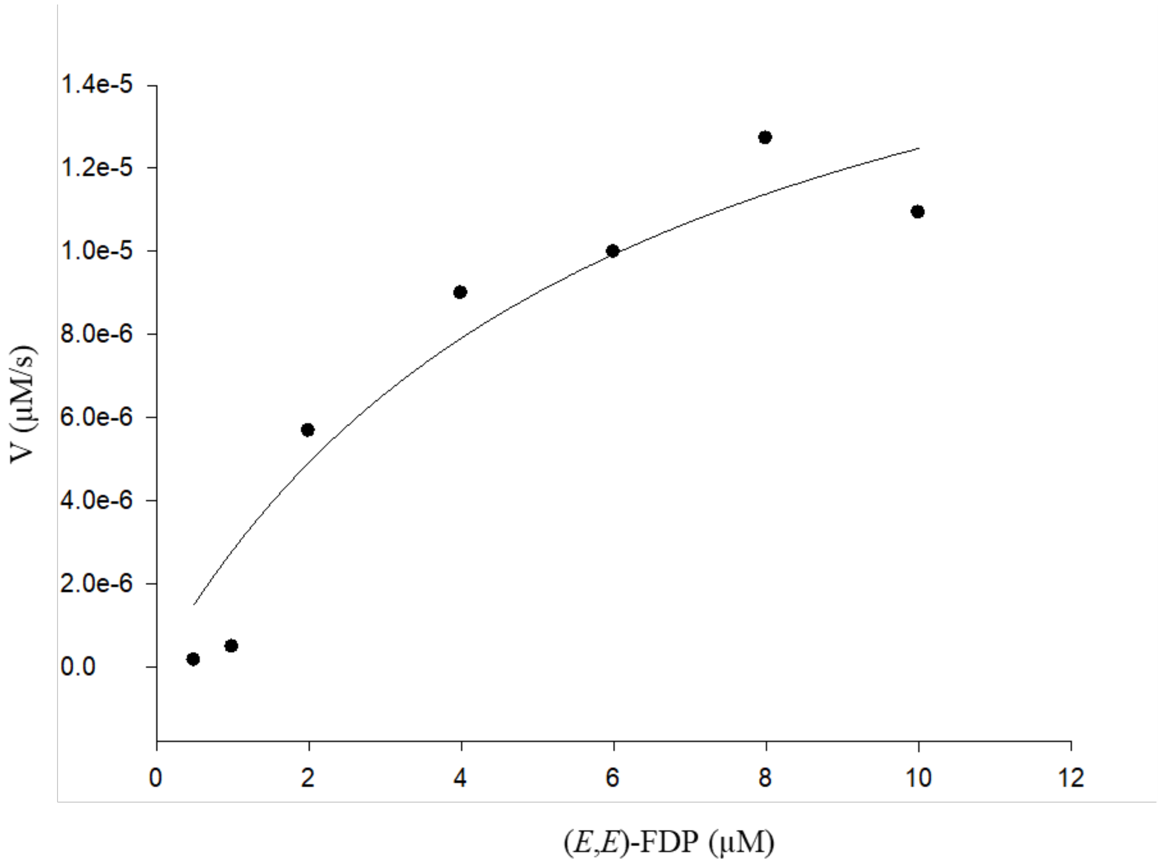

Figure S55: Representative Michaelis-Menten plot for the conversion of [1-^{3}H]-FDP by SdS A301D.

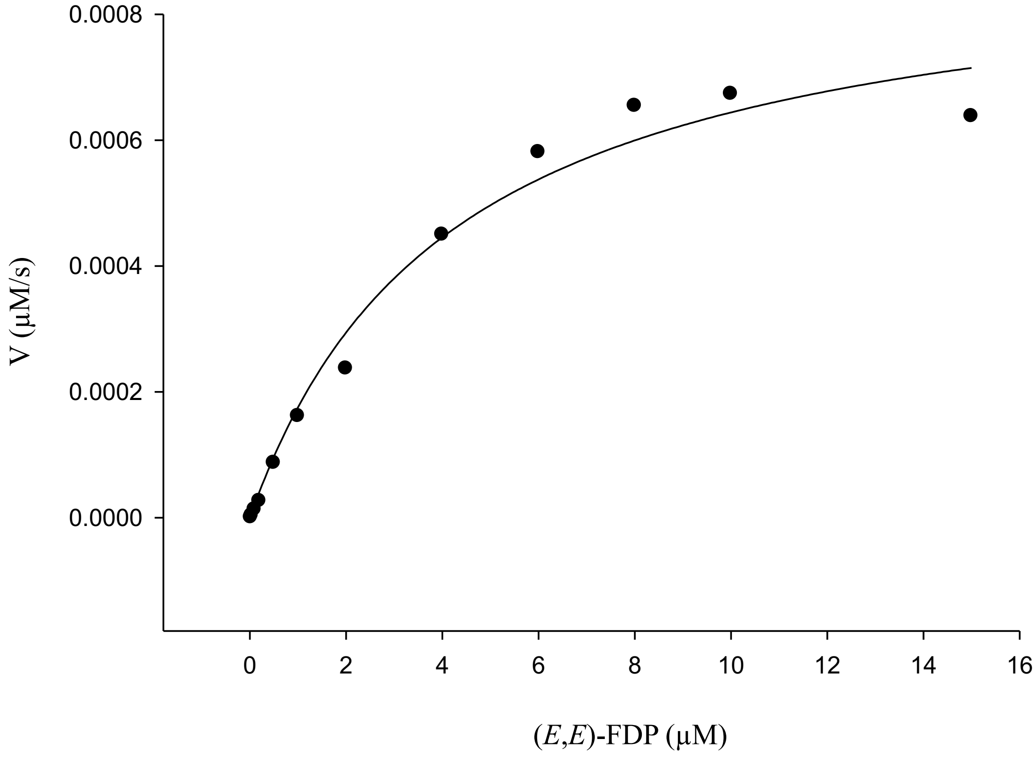

Figure S56: Representative Michaelis-Menten plot for the conversion of [1-^{3}H]-FDP by SdS A301S.

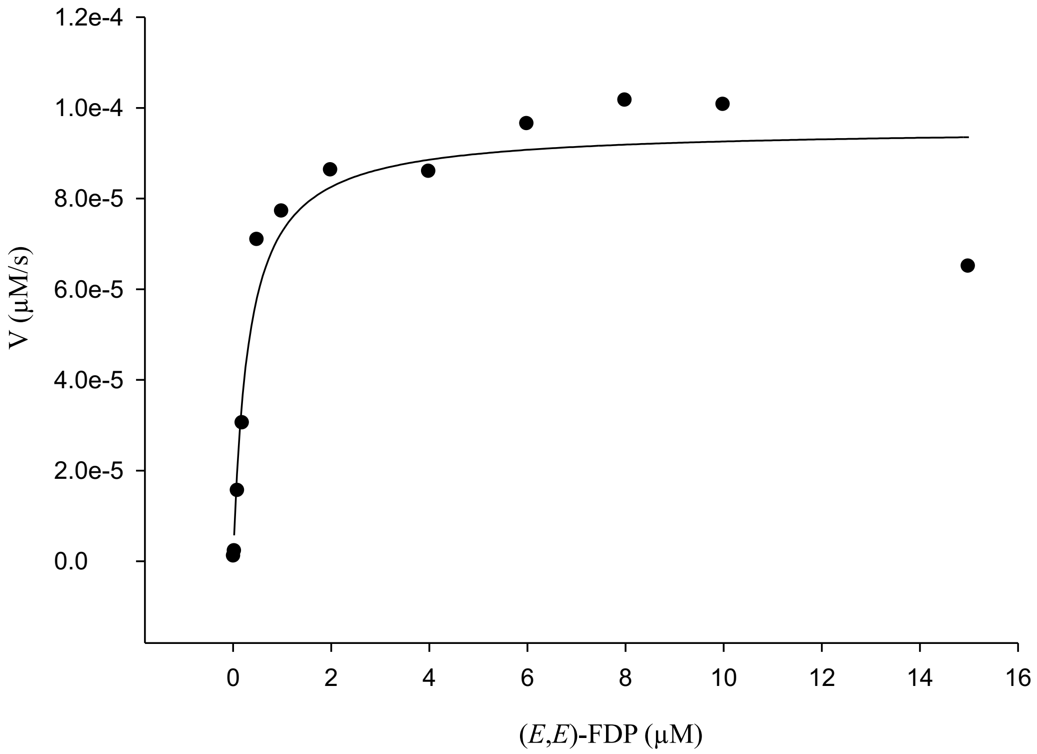

Figure S57: Representative Michaelis-Menten plot for the conversion of [1-^{3}H]-FDP by SdS G305H.

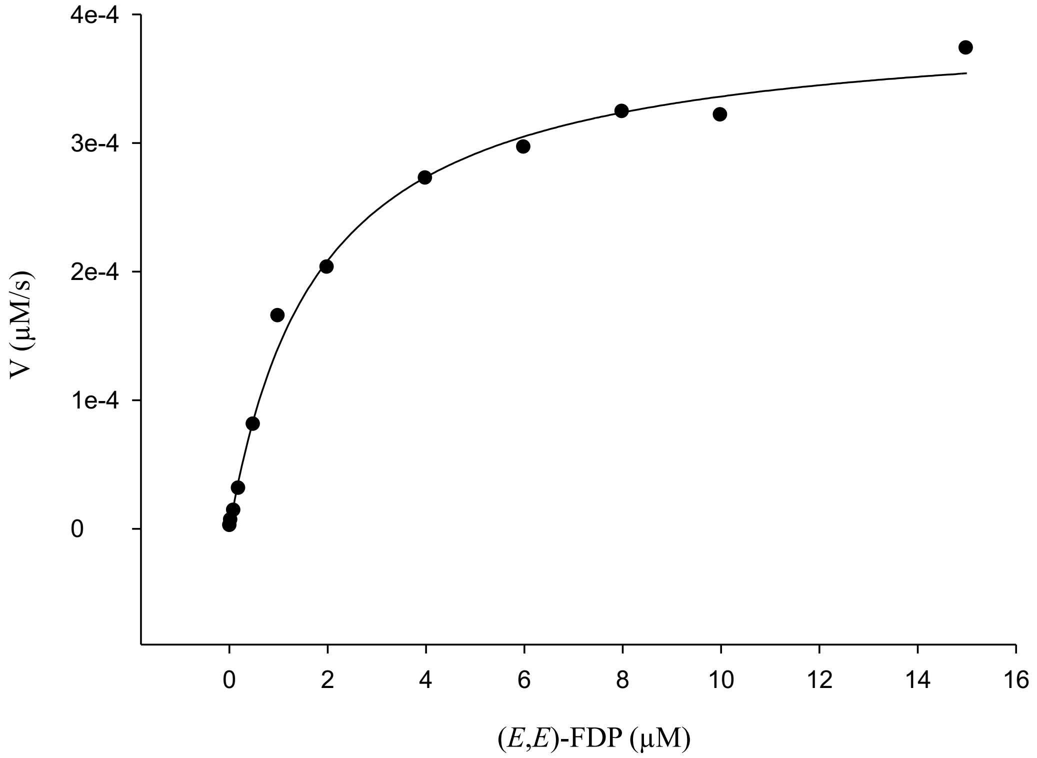

Figure S58: Representative Michaelis-Menten plot for the conversion of [1-^{3}H]-FDP by SdS G305E.

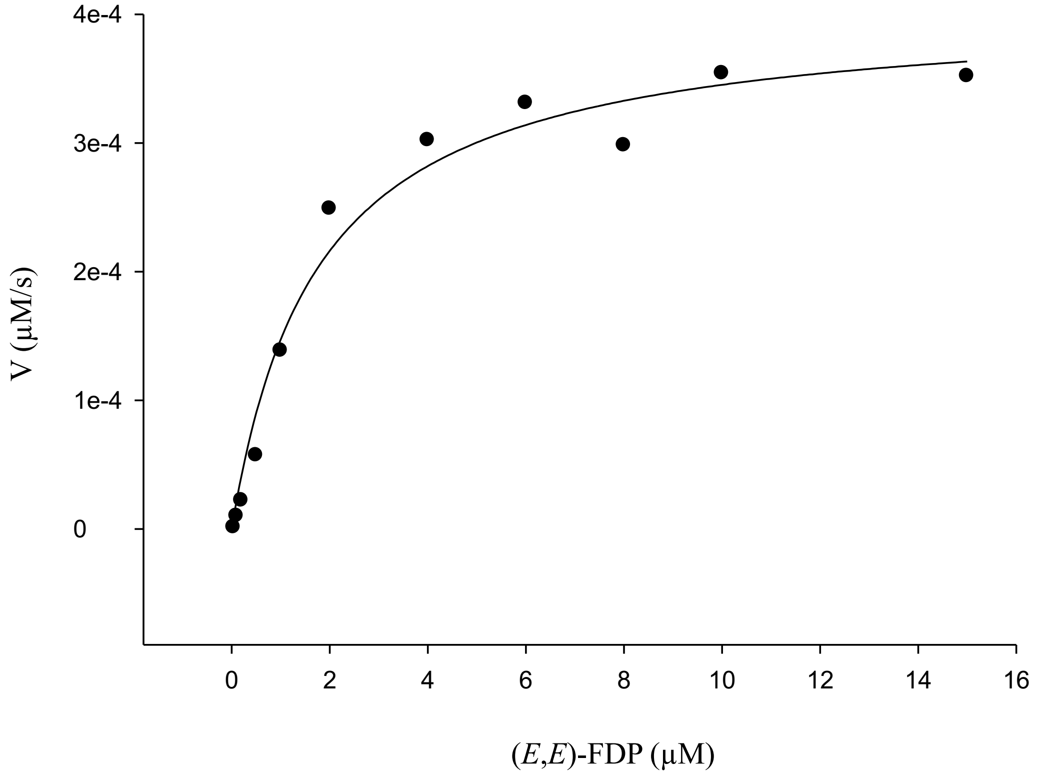

Figure S59: Representative Michaelis-Menten plot for the conversion of [1-^{3}H]-FDP by SdS G305E pH 6.0.

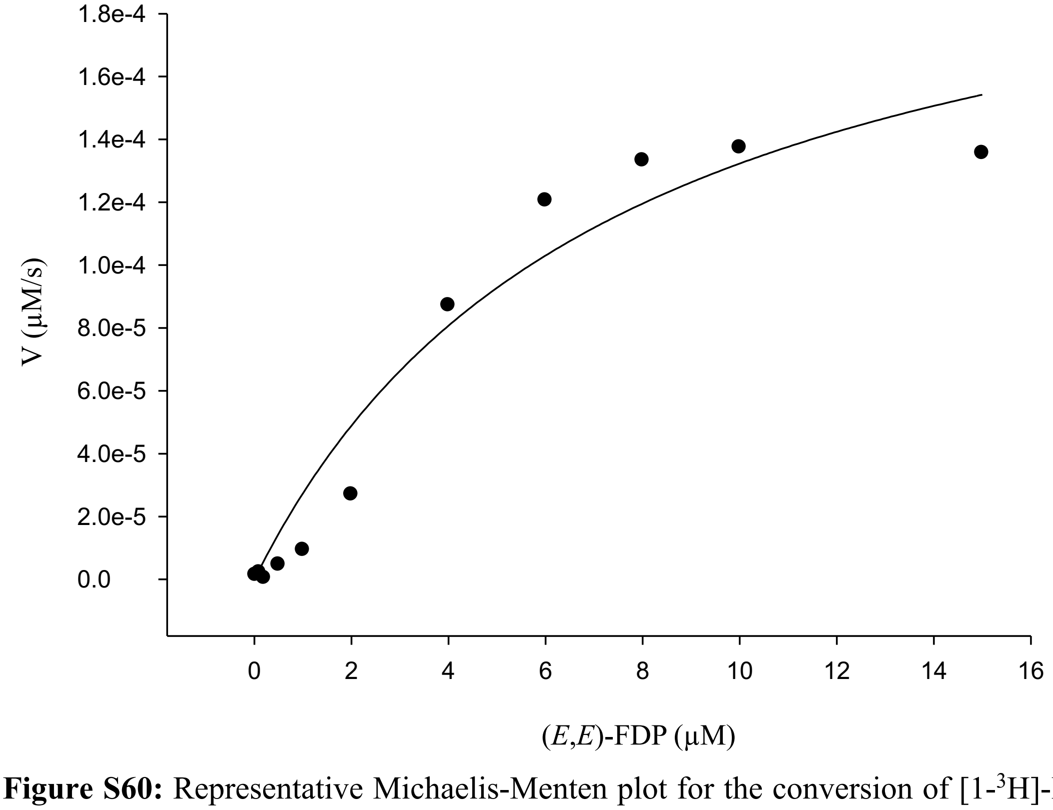

Figure S60: Representative Michaelis-Menten plot for the conversion of [1-^{3}H]-FDP by SdS G305D.

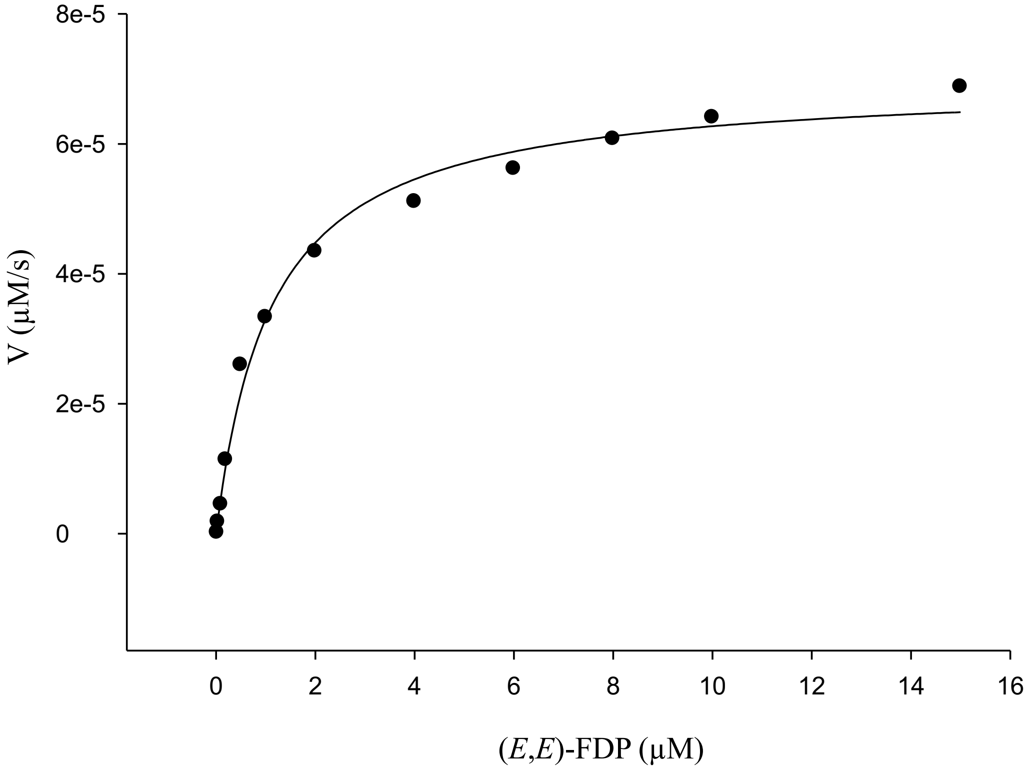

Figure S61: Representative Michaelis-Menten plot for the conversion of [1-^{3}H]-FDP by SdS A301N+G305E.

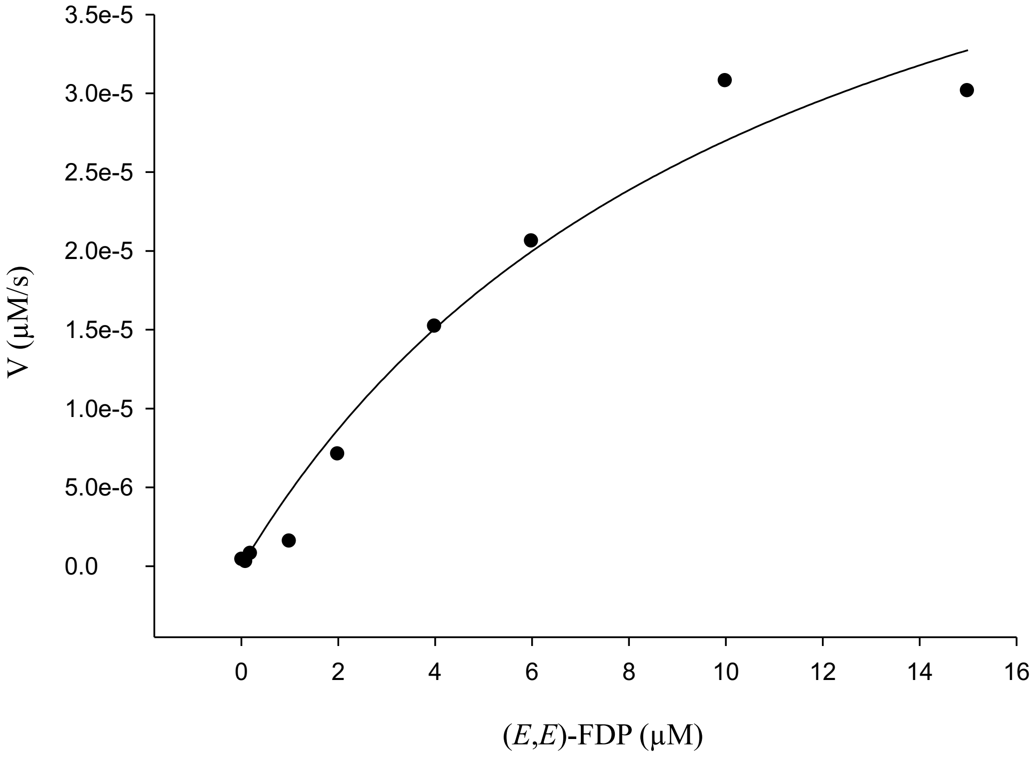

Figure S62: Representative Michaelis-Menten plot for the conversion of [1-^{3}H]-FDP by SdS A301Y+G305E.

15. NMR Spectra

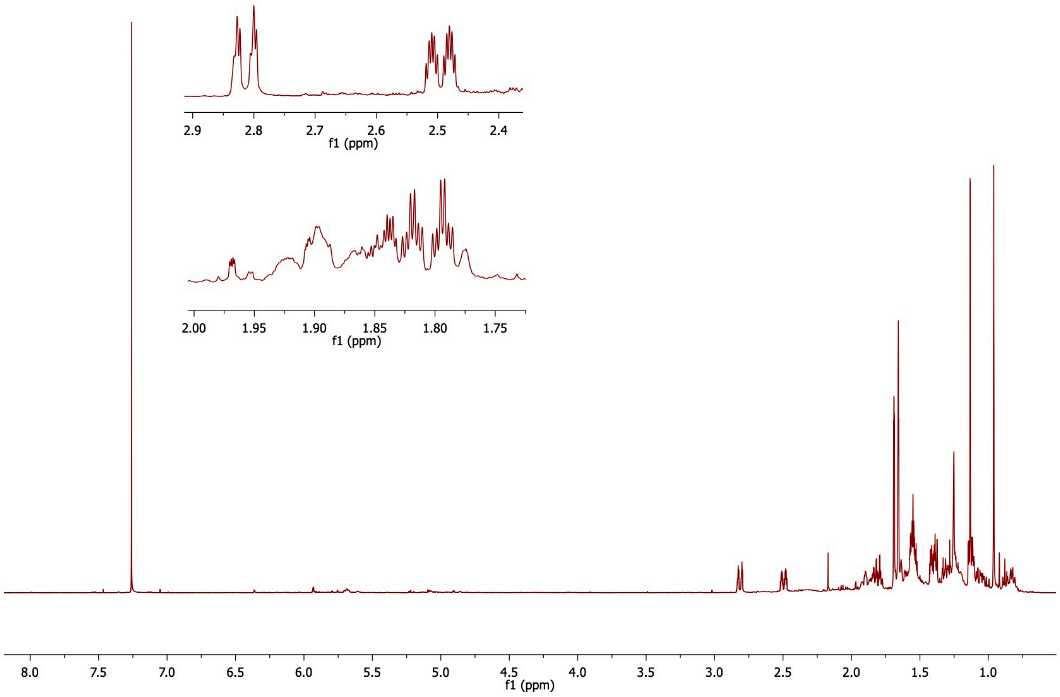

Figure 63: ^{1}H NMR spectrum (500 MHz, $CDCl_3$, 298K) of selin-7(11)-en-4-ol (**10**) full and zoom between 1.75 to 2.9 ppm.

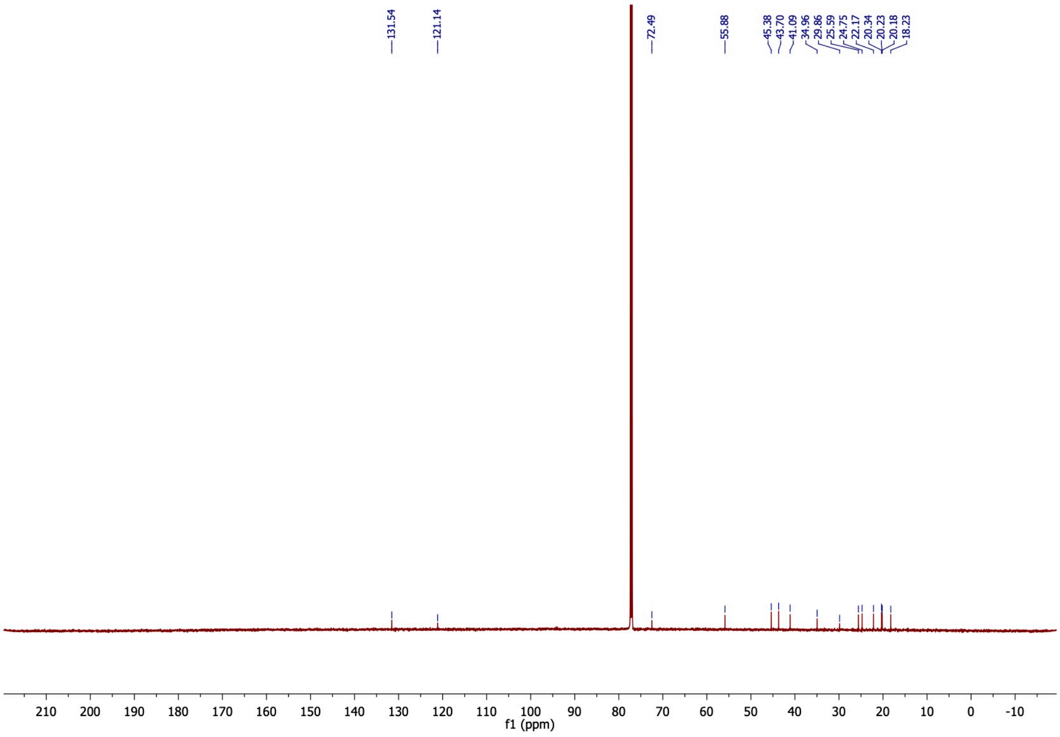

Figure 64: ^{13}C NMR spectrum (500 MHz, $CDCl_3$, 298K) of selin-7(11)-en-4-ol (**10**). Peak at 29.86 represents the contamination of grease.

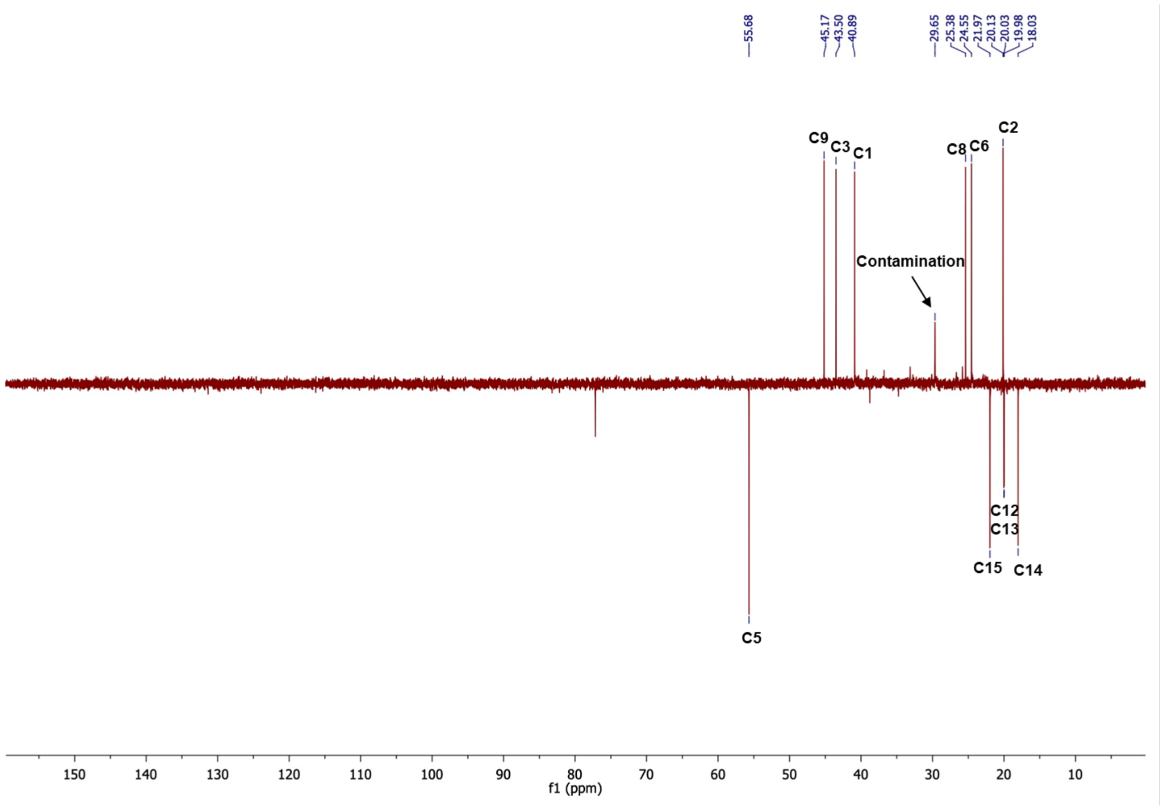

Figure 65: DEPT 135 NMR spectrum (500 MHz, $CDCl_3$, 298K) of selin-7(11)-en-4-ol (**10**). Peak at 29.65 represent the contamination of grease.

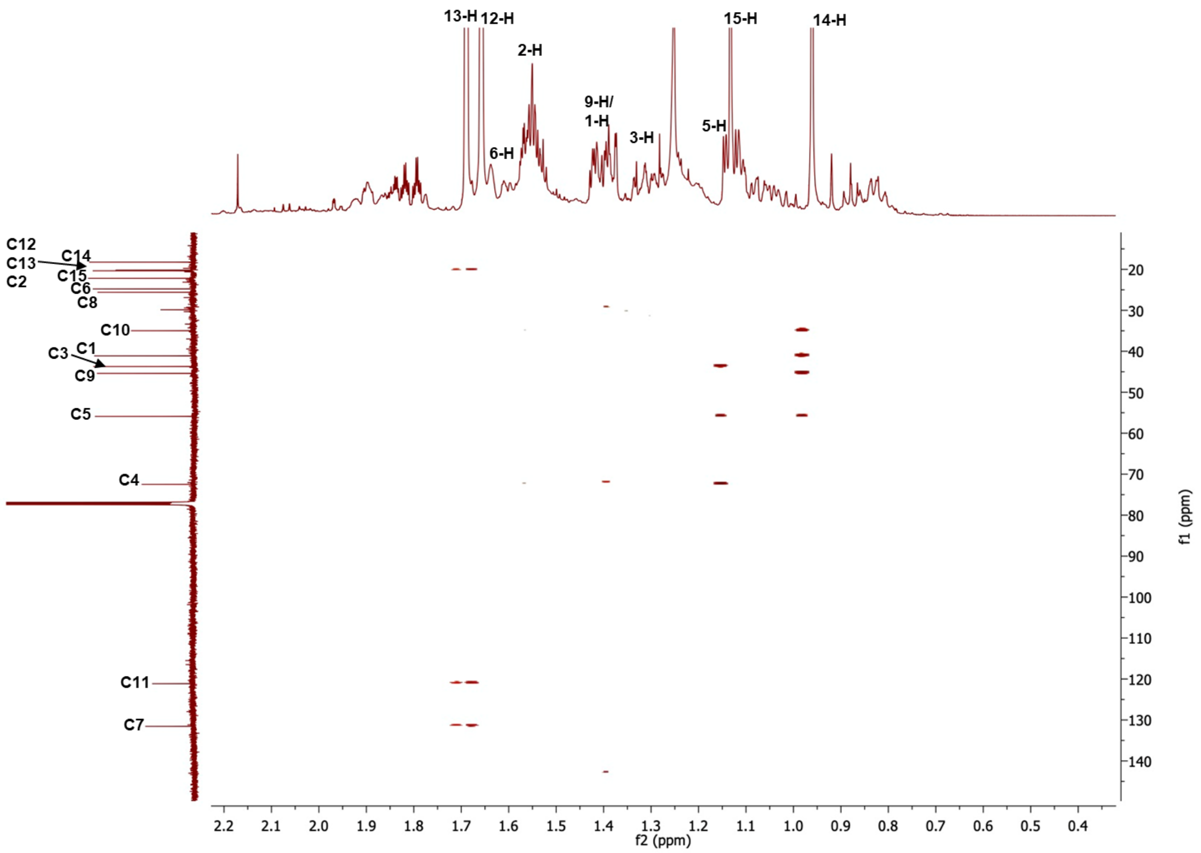

Figure 66A: Edited HMBC NMR spectrum (500 MHz, $CDCl_3$, 298K) of selin-7(11)-en-4-ol (**10**).

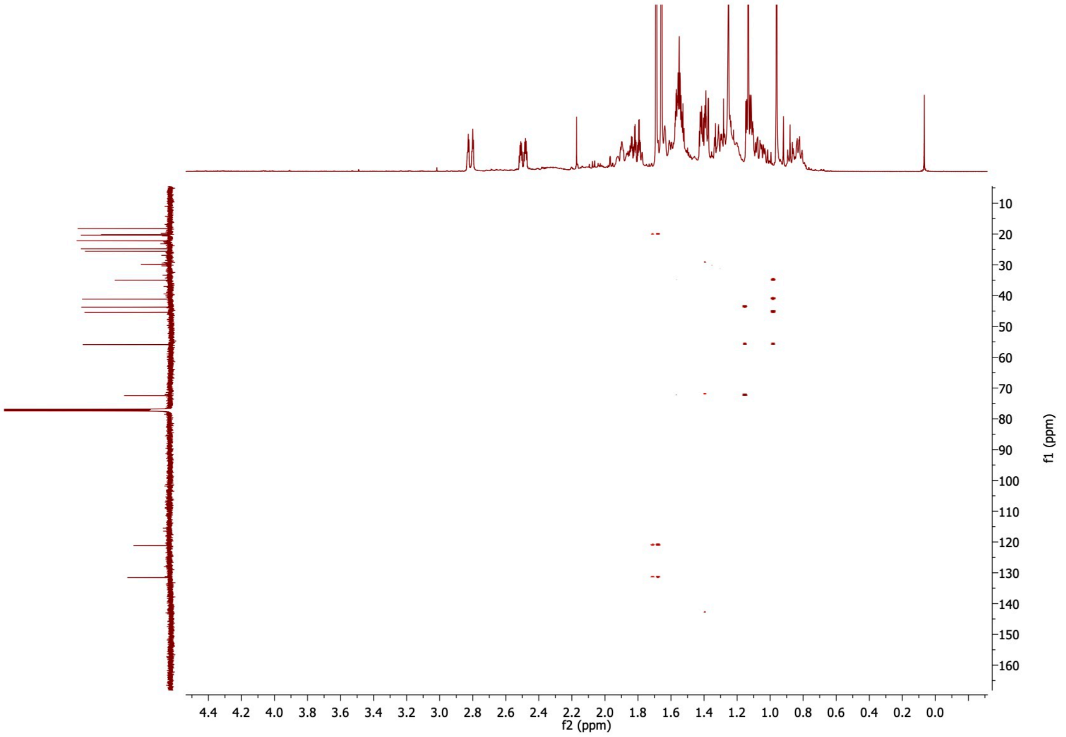

Figure 66B: HMBC NMR spectrum (500 MHz, $CDCl_3$, 298K) of selin-7(11)-en-4-ol (**10**).

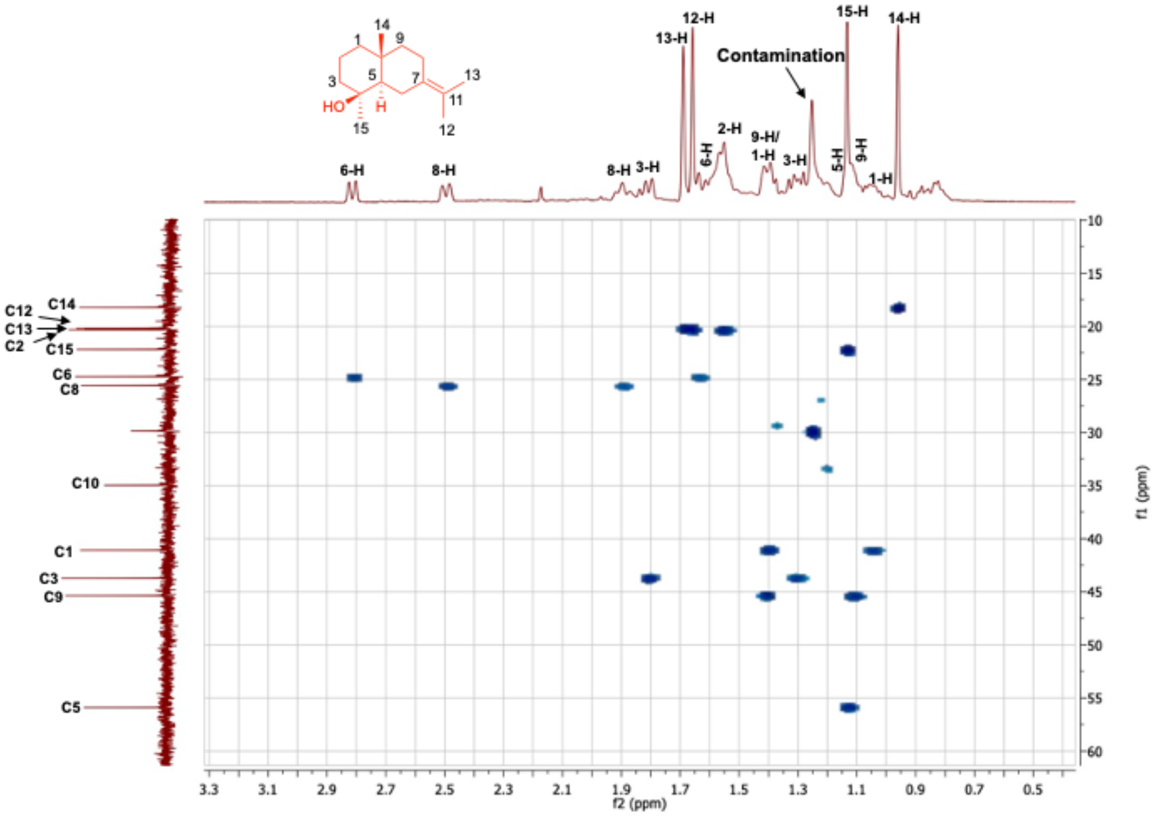

Figure 67A: Edited HSQC NMR spectrum (500 MHz, $CDCl_3$, 298K) of selin-7(11)-en-4-ol (**10**).

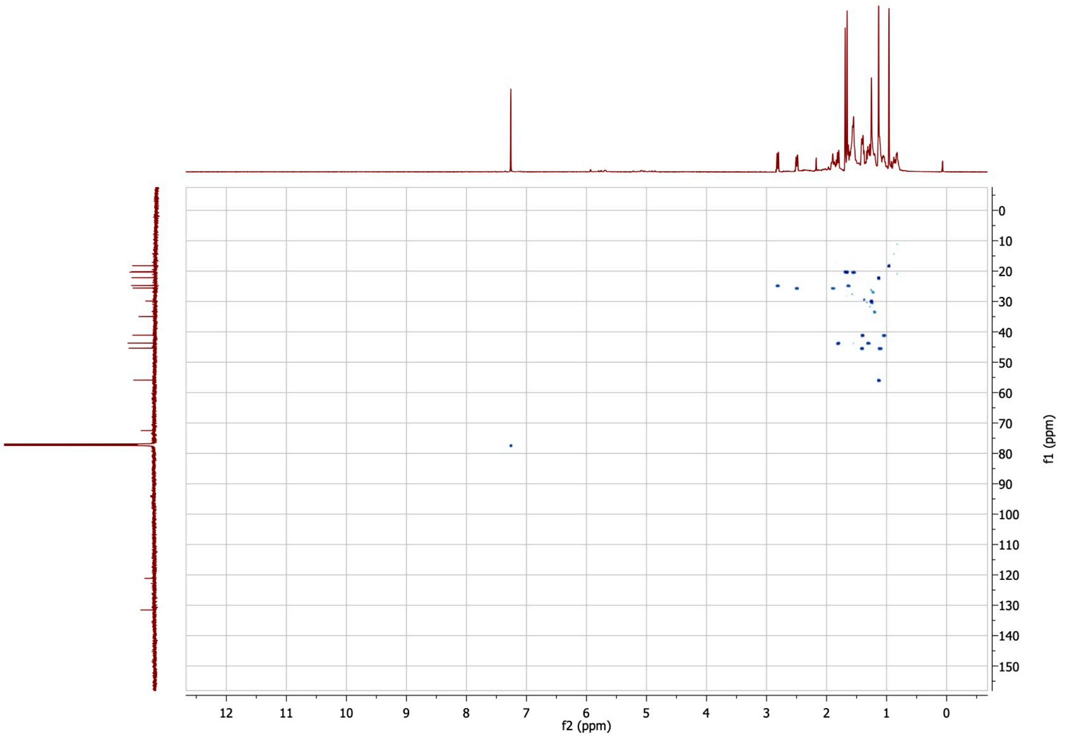

Figure 67B: HSQC NMR spectrum (500 MHz, $CDCl_3$, 298K) of selin-7(11)-en-4-ol (**10**).

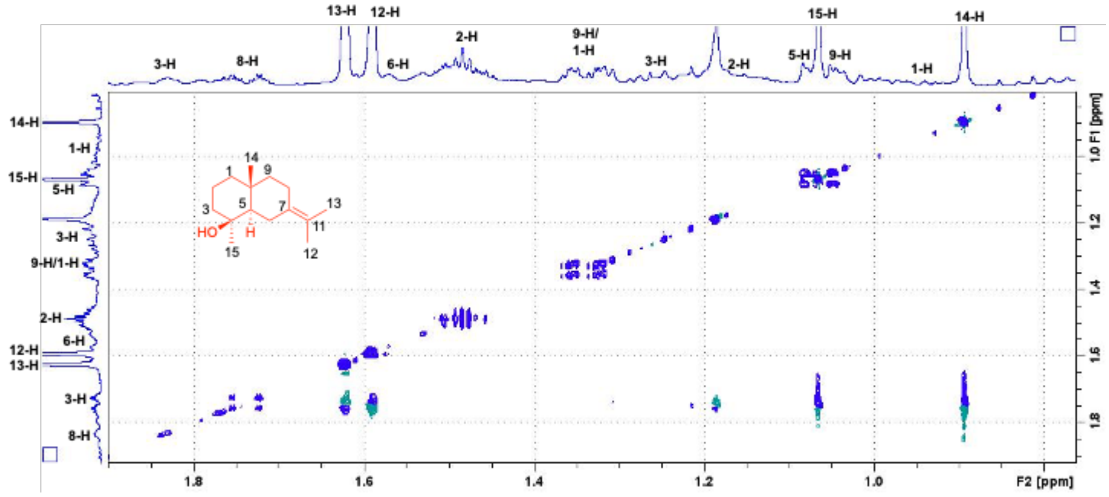

Figure S68A: Edited NOESY NMR spectrum (400 MHz, $CDCl_3$, 298K) of selin-7(11)-en-4-ol (**10**).

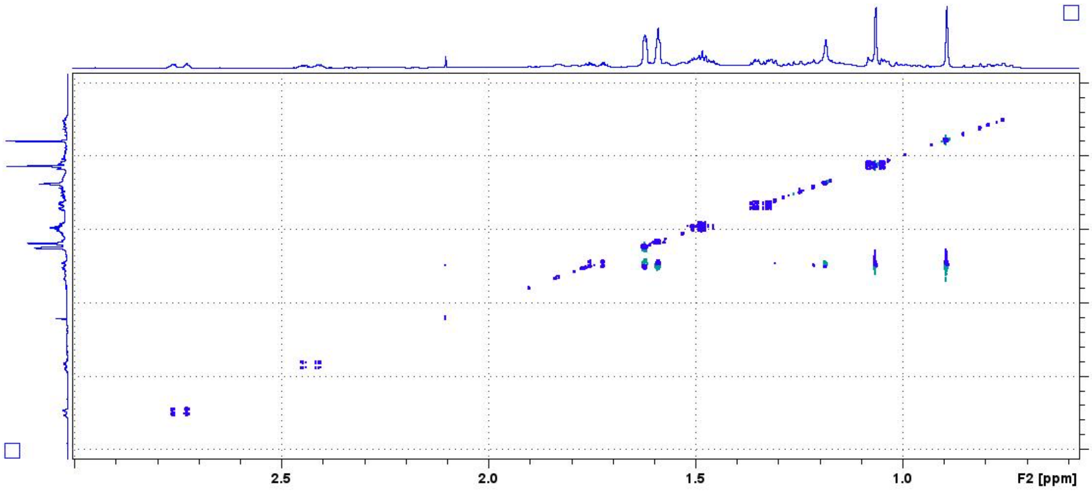

Figure S68B: NOESY NMR spectrum (400 MHz, $CDCl_3$, 298K) of selin-7(11)-en-4-ol (**10**).

16. References

(1) Baer, P.; Rabe, P.; Fischer, K.; Citron, C. A.; Klapschinski, T. A.; Groll, M.; Dickschat, J. S. Induced-Fit Mechanism in Class I Terpene Cyclases. *Angewandte Chemie International Edition* **2014**, *53*, 7652–7656.

(2) Davisson, V. J.; Woodside, A. B.; Neal, T. R.; Stremler, K. E.; Muehlbacher, M.; Poulter, C. D. Phosphorylation of Isoprenoid Alcohols. *Journal of Organic Chemistry* **1986**, *51*, 4768–4779.

(3) Bradford, M. M. A Rapid and Sensitive Method for the Quantitation of Microgram Quantities of Protein Utilizing the Principle of Protein-Dye Binding. *Analytical Biochemistry* **1976**, *72*, 248–254.

(4) Grundy, D. J.; Chen, M.; González, V.; Leoni, S.; Miller, D. J.; Christianson, D. W.; Allemann, R. K. Mechanism of Germacradien-4-Ol Synthase-Controlled Water Capture. *Biochemistry* **2016**, *55*, 2112–2121.

(5) Martin, V. J. J.; Pitera, D. J.; Withers, S. T.; Newman, J. D.; Keasling, J. D. Engineering a Mevalonate Pathway in *Escherichia coli* for Production of Terpenoids. *Nat Biotechnol* **2003**, *21*, 796–802.

(6) *http://nbcr-222.ucsd.edu/pdb2pqr_2.1.1/.*

(7) Dolinsky, T. J.; Czodrowski, P.; Li, H.; Nielsen, J. E.; Jensen, J. H.; Klebe, G.; Baker, N. A. PDB2PQR: Expanding and Upgrading Automated Preparation of Biomolecular Structures for Molecular Simulations. *Nucleic Acids Research* **2007**, *35* (SUPPL.2), W522–W525.

(8) Dolinsky, T. J.; Nielsen, J. E.; McCammon, J. A.; Baker, N. A. PDB2PQR: An Automated Pipeline for the Setup of Poisson-Boltzmann Electrostatics Calculations. *Nucleic Acids Research* **2004**, *32* (Web Server), W665–W667.

(9) Li, H.; Robertson, A. D.; Jensen, J. H. Very Fast Empirical Prediction and Rationalization of Protein pKa Values. *Proteins: Structure, Function, and Bioinformatics* **2005**, *61*, 704–721.

(10) Bas, D. C.; Rogers, D. M.; Jensen, J. H. Very Fast Prediction and Rationalization of pKa Values for Protein-Ligand Complexes. *Proteins* **2008**, *73*, 765–783.

(11) Brooks, B. R.; Brooks, C. L.; Mackerell, A. D.; Nilsson, L.; Petrella, R. J.; Roux, B.; Won, Y.; Archontis, G.; Bartels, C.; Boresch, S.; Caflisch, A.; Caves, L.; Cui, Q.; Dinner, A. R.; Feig, M.; Fischer, S.; Gao, J.; Hodoscek, M.; Im, W.; Kuczera, K.; Lazaridis, T.; Ma, J.; Ovchinnikov, V.; Paci, E.; Pastor, R. W.; Post, C. B.; Pu, J. Z.; Schaefer, M.; Tidor, B.; Venable, R. M.; Woodcock, H. L.; Wu, X.; Yang, W.; York, D. M.; Karplus, M. CHARMM: The Biomolecular Simulation Program. *Journal of Computational Chemistry* **2009**, *30*, 1545–1614.

(12) Humphrey, W.; Dalke, A.; Schulten, K. VMD : Visual Molecular Dynamics. *Journal of Molecular Graphics* **1996**, *14*, 33–38.

(13) Srivastava, P. L.; Escorcia, A. M.; Huynh, F.; Miller, D. J.; Allemann, R. K.; Van Der Kamp, M. W. Redesigning the Molecular Choreography to Prevent Hydroxylation in Germacradien-11-Ol Synthase Catalysis. *ACS Catal* **2021**, *11*, 1033–1041.

(14) Srivastava, P. L.; Johns, S. T.; Walters, R.; Miller, D. J.; Van der Kamp, M. W.; Allemann, R. k. Active Site Loop Engineering Abolishes Water

Capture in Hydroxylating Sesquiterpene Synthases. *ACS Catal* **2023**, *13*, 14199–14204.
(15) Best, R. B.; Zhu, X.; Shim, J.; Lopes, P. E. M.; Mittal, J.; Feig, M.; MacKerell, A. D.; Jr. Optimization of the Additive CHARMM All-Atom Protein Force Field Targeting Improved Sampling of the Backbone φ, ψ and Side-Chain X1 and X2 Dihedral Angles. *Journal of chemical theory and computation* **2012**, *8*, 3257.
(16) Van der Kamp, M. W.; Sirirak, J.; Zurek, J.; Allemann, R. K.; Mulholland, A. J. Conformational Change and Ligand Binding in the Aristolochene Synthase Catalytic Cycle. *Biochemistry* **2013**, *52*, 8094–8105.
(17) Allnér, O.; Nilsson, L.; Villa, A. Magnesium Ion–Water Coordination and Exchange in Biomolecular Simulations. *Journal of Chemical Theory and Computation* **2012**, *8*, 1493–1502.
(18) Jo, S.; Kim, T.; Iyer, V. G.; Im, W. CHARMM-GUI: A Web-Based Graphical User Interface for CHARMM. *J Comput Chem* **2008**, *29*, 1859–1865.
(19) Vanommeslaeghe, K.; Hatcher, E.; Acharya, C.; Kundu, S.; Zhong, S.; Shim, J.; Darian, E.; Guvench, O.; Lopes, P.; Vorobyov, I.; Mackerell, A. D. CHARMM General Force Field: A Force Field for Drug-like Molecules Compatible with the CHARMM All-Atom Additive Biological Force Fields. *J Comput Chem* **2010**, *31*, 671–690.
(20) Vanommeslaeghe, K.; D. MacKerell Jr., A. Automation of the CHARMM General Force Field (CGenFF) I: Bond Perception and Atom Typing. *J Chem Inf Model* **2012**, *52*, 3144–3154.
(21) Vanommeslaeghe, K.; Prabhu Raman, E.; D. MacKerell Jr., A. Automation of the CHARMM General Force Field (CGenFF) II: Assignment of Bonded Parameters and Partial Atomic Charges. *J Chem Inf Model* **2012**, *52*, 3155–3168.
(22) Das, S.; Dixit, M.; Major, D. T. First Principles Model Calculations of the Biosynthetic Pathway in Selinadiene Synthase. *Bioorg Med Chem* **2016**, *24*, 4867–4870.
(23) Wang, Y. H.; Xu, H.; Zou, J.; Chen, X. B.; Zhuang, Y. Q.; Liu, W. L.; Celik, E.; Chen, G. D.; Hu, D.; Gao, H.; Wu, R.; Sun, P. H.; Dickschat, J. S. Catalytic Role of Carbonyl Oxygens and Water in Selinadiene Synthase. *Nat Catal* **2022**, *5*, 128–135.
(24) Dachriyanus; Bakhtiar, A.; Sargent, M. V.; Skelton, B. W.; White, A. H. Rac-Eudesm-7(11)-En-4-Ol. *Acta Crystallogr C* **2004**, *60*, 2000–2001.
(25) Bohlmann, F.; Zdero, C.; King, R. M.; Robinson, H. Humulene Derivatives from *Acritopappus prunifolius*. *Phytochemistry* **1982**, *21*, 147–150.
(26) Yu Zhao; Yue, J.; Llin, Z.; Ding, J.; Sun, H. Eudesmane Sesquiterpenes from *Laggera Pterodonta*. *Phytochemistry* **1997**, *44*, 459–464.
