# Supplementary material for: Simulation-Guided Engineering Enables a Functional Switch in Selinadiene Synthase toward Hydroxylation
Source: ACS Catal. 2024 Jul 9;14(14):11034–43. doi: 10.1021/acscatal.4c02032 (PMC11264211; doi:10.1021/acscatal.4c02032)
Supplement: Supplementary file 2 — cs4c02032_si_002.zip [file cs4c02032_si_002.zip › sds_md/README.docx]

**Files related to MD simulations in the following publication:**

**Simulation-guided engineering enables a functional switch in selinadiene synthase towards hydroxylation**

*Prabhakar L. Srivastava, ^†^ Sam T. Johns,^‡^ Angus Voice, ^‡^ Katharine Morley,^‡^ Andrés M. Escorcia,^‡^ David J. Miller,^†^ Rudolf K. Allemann*^,†^ and Marc W. van der Kamp*^,‡^*

^†^School of Chemistry, Cardiff University, Main Building, Park Place, Cardiff CF10 3AT, United Kingdom.

^‡^School of Biochemistry, University of Bristol, University Walk, Bristol BS8 1TD, United Kingdom.

***Files related to MD simulation of SdS•FDP complexes* (sds_md/fdp/)**

*md_input_files/* – minimisation, heating, equilibration, and production MD amber input files, as well as restraints for the magnesium ion – FDP oxygen distances (mgores.rest).

Order in which the files are run is as follows: minh, minwat, min_all, heating, equi_npt, pressure_p1, pressure_p2, pressure_p3, pressure_p4, pressure_p5, production_30

*starting_structures_param/* - starting structures (solvated) for the simulations (pdb files) and amber topology file with parameters (.parm)

***Files related to MD simulation of SdS•FDP complexes*** **(sds_md/ccd/)**

*md_input_files/* – minimisation, heating, equilibration, and production MD amber input files

Order in which the files are run is as follows: minh, minwat, min_all, heat, equi_npt, equi_p1, equi_p2, equi_p3, equi_p4, equi_p5, equi_p6, md1_1ns

*params/* - CGenFF parameter and topology files for carbacation B (ccd.prm, ccd.rtf) and for PPi (opp.prm, opp.rtf)

*wt/* - starting structure (pdb, rst7) and topology file (parm7) for simulation of SDS_WT_

*g305e/* - starting structure (pdb, rst7) and topology file (parm7) for simulation of SDS G305E
